# Exploring Subsite Selectivity within *Plasmodium vivaxN*-Myristoyltransferase Using Pyrazole-Derived Inhibitors

**DOI:** 10.1021/acs.jmedchem.4c00168

**Published:** 2024-04-29

**Authors:** Diego Rodríguez-Hernández, Michael K. Fenwick, Rachael Zigweid, Banumathi Sankaran, Peter J. Myler, Per Sunnerhagen, Alexis Kaushansky, Bart L. Staker, Morten Grøtli

**Affiliations:** † Department of Chemistry and Molecular Biology, University of Gothenburg, S-405 30 Gothenburg, Sweden; ‡ Department of Structural and Functional Biology, Synthetic Biology Laboratory, Institute of Biology, University of Campinas, Campinas, SP 13083-862, Brazil; § Seattle Structural Genomics Center for Infectious Disease, Seattle, Washington 98109, United States; ∥ Center for Global Infectious Disease Research, Seattle Children’s Research Institute, Seattle, Washington 98109, United States; ⊥ Molecular Biophysics and Integrated Bioimaging, Berkeley Center for Structural Biology, Advanced Light Source, Berkeley National Laboratory, Berkeley, California 94720, United States; # Department of Pediatrics, University of Washington, Seattle, Washington 98195, United States

## Abstract

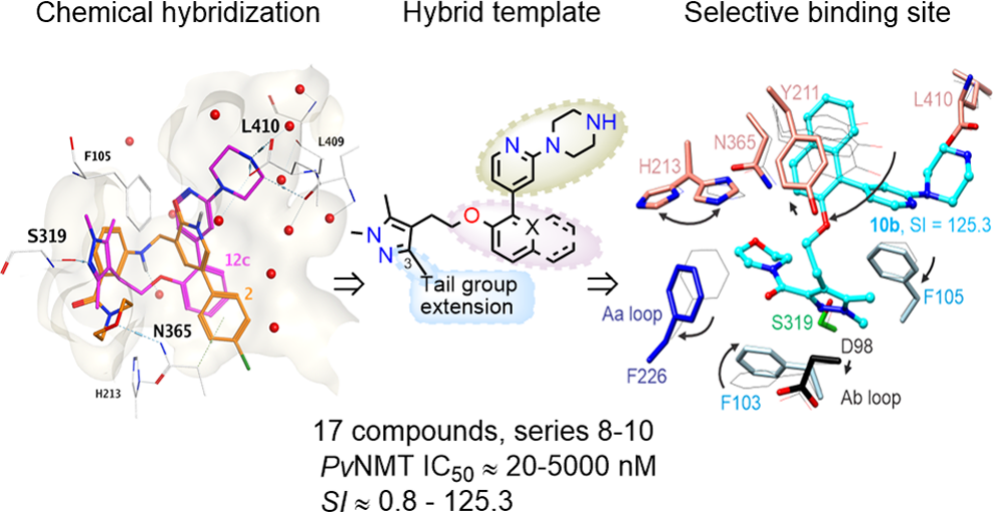

*N*-myristoyltransferase (NMT) is a promising antimalarial drug target. Despite biochemical similarities between *Plasmodium vivax* and human NMTs, our recent research demonstrated that high selectivity is achievable. Herein, we report *Pv*NMT-inhibiting compounds aimed at identifying novel mechanisms of selectivity. Various functional groups are appended to a pyrazole moiety in the inhibitor to target a pocket formed beneath the peptide binding cleft. The inhibitor core group polarity, lipophilicity, and size are also varied to probe the water structure near a channel. Selectivity index values range from 0.8 to 125.3. Cocrystal structures of two selective compounds, determined at 1.97 and 2.43 Å, show that extensions bind the targeted pocket but with different stabilities. A bulky naphthalene moiety introduced into the core binds next to instead of displacing protein-bound waters, causing a shift in the inhibitor position and expanding the binding site. Our structure–activity data provide a conceptual foundation for guiding future inhibitor optimizations.

## Introduction

Malaria, a severe and potentially fatal illness, is caused by particular parasites belonging to the *Plasmodium* genus. The primary mode of transmission is through the bite of female Anopheles mosquitoes carrying the parasite. Six *Plasmodium* species—*Plasmodium malariae*, *Plasmodium falciparum*, *Plasmodium vivax*, *Plasmodium ovale curtisi*, *Plasmodium ovale wallikeri*, and *Plasmodium knowlesi*— are known to infect humans.^1,2^ In 2022, there were an estimated 249 million cases of malaria worldwide in 85 malaria-endemic countries, resulting in approximately 608,000 deaths. The vulnerability of children, who account for a significant proportion of these reported deaths, is of particular concern. *P. falciparum* and *P. vivax* are responsible for nearly all malaria-related deaths. *P. falciparum* is the primary cause of malaria in Africa and is also prevalent in the Eastern Mediterranean and Western Pacific regions.^1^ Conversely, *P. vivax* is more widespread in Southeast Asia and the Americas, and around 73% of reported cases in the Americas are concentrated in Venezuela, Brazil, and Colombia.^1^

While a wide variety of successful antimalarial drugs are currently on the market (e.g., Artemisinin-based combination therapy for blood-stage parasites), there exist major gaps in drug coverage. These gaps primarily have two origins: first, the propensity of *P. vivax* to differentiate into largely quiescent forms in the liver, termed hypnozoites, is associated with increased recalcitrance to existing drugs. Hypnozoite-induced relapses account for 80% of *P. vivax* malaria cases^3^ and can be triggered weeks to months after the initial infection.^4^ Second, many antimalarial drugs target parasites through related mechanisms of action and thus are not effective in drug-resistant parasites.^5^ Primaquine and tafenoquine are the only licensed drugs available that are parasiticidal against all liver life cycle stages, thus preventing relapse. However, both drugs can cause severe hemolysis in persons with glucose-6-phosphate dehydrogenase (G6PD) deficiency,^6^ which affects up to 30% of the population in regions where malaria is present.^7^ By exploring *PlasmodiumN*-myristoyltransferase (NMT) as a target, we hope to overcome both roadblocks.

The genomes of *P. falciparum* and *P. vivax* contain approximately 5400 genes.^8,9^ Within this genetic framework, identifying indispensable drug targets such as NMT that directly or indirectly span various phases of the *Plasmodium* life cycle through their biochemical activity, would constitute significant progress. NMT is now an established drug target for combating malaria and other infectious diseases caused by protozoan parasites,^10−12^ with crystal structure platforms for structure-aided inhibitor development against *Cryptosporidium parvum*,^13^*Leishmania donovani* and *major*,^14,15^ and *P. vivax*,^16^ already available.

NMT cotranslationally transfers the myristate moiety of myristoyl-coenzyme A (MyrCoA) to Gly2 of protein substrates after removal of the *N*-terminal methionine.^17−19^*P. vivax* and *P. falciparum* express a single NMT protein, whereas human cells express two closely related isotypes, *Hs*NMT1 and *Hs*NMT2.^20^*Pv*NMT and *Pf*NMT have more than 80% shared identity, while the human orthologs are only about 40% identical in sequence to *Pv*NMT and *Pf*NMT.^21^ Chemical proteomics studies have identified a multitude of potential substrates of *Plasmodium* and human NMTs, suggesting involvement in regulating many cellular processes.^22,23^ Small-molecule inhibitors bind both *Pv*NMT and *Hs*NMT1 in the peptide binding cleft, where residues contacting the ligands via side chain atoms share 100% sequence identity. This presents a major challenge for the development of compounds that bind *Plasmodium* NMTs selectively, especially *Pv*NMT (the binding site of *Pf*NMT contains one amino acid difference).

**Figure 1:**
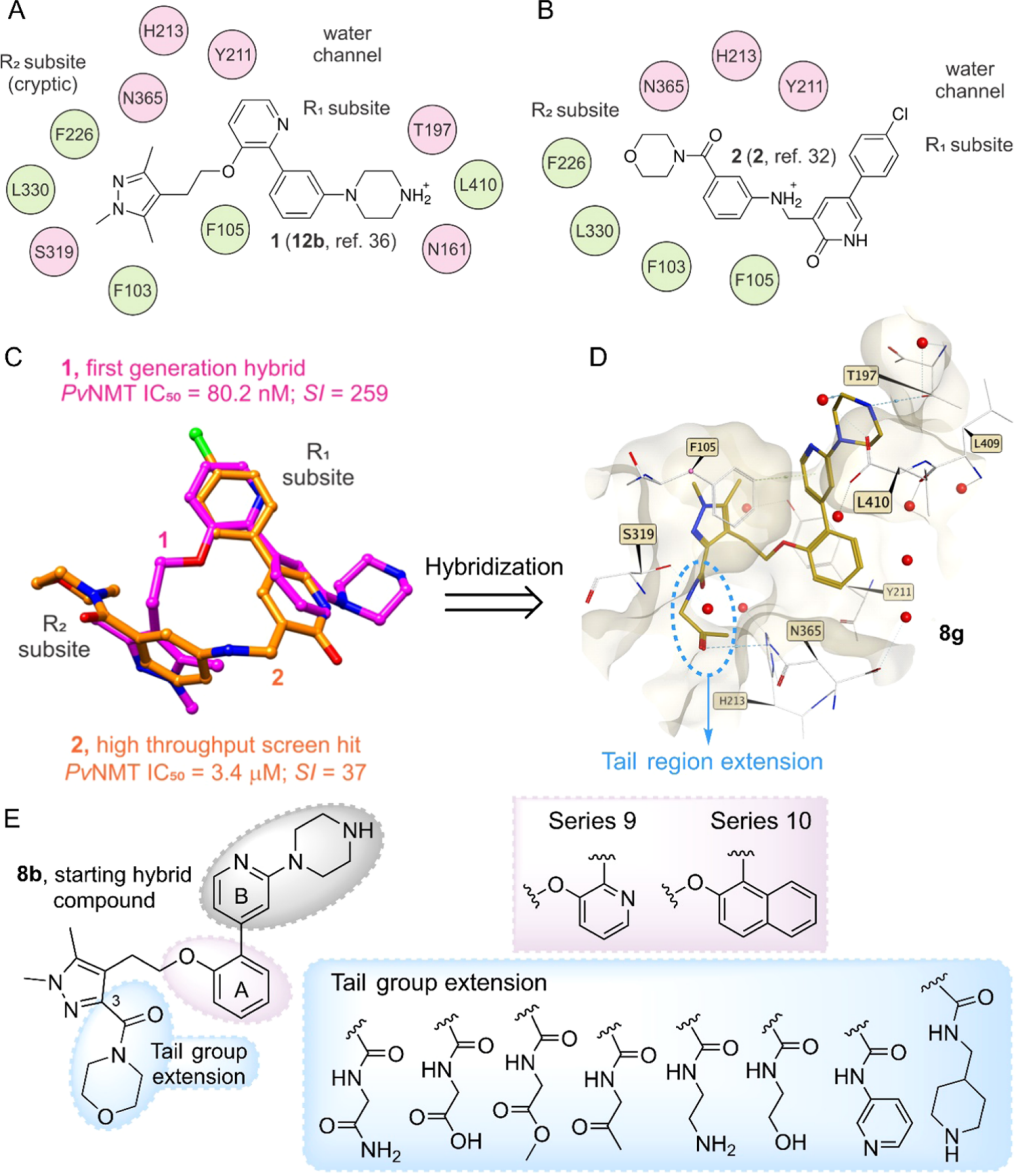
Hybridization approach for exploring selective subsites. (A) Target subsites, based on complex with **1**. (B) Target subsites, based on complex with **2**. (C) Distinct but overlapping binding modes of **1**(36) and **2**.^32^ (D) Designed compound (**8g**) docked into *Pv*NMT crystal structure (PDB code 6MB1). Surface representations in front of the binding site are removed for clarity. Interactions involving atoms of compound **8g** are drawn using dashed lines, and red spheres represent water molecules. (E) Design of hybrid compounds. On the left is **8b**, a starting hybrid compound, having its Core A and tail group extension shaded purple and blue, respectively. This is juxtaposed with the corresponding sets of functional group substitutions (boxed). The Core B and head groups (gray) were not varied.

In the past decade, a variety of potent *Plasmodium* NMT inhibitors capable of killing the parasite have been designed and synthesized. However, although potencies have risen dramatically, the selectivities over the human enzymes have increased more slowly.^16,24−30^ In 2018, guided by crystal structure data, a fragment merging approach led to the design of IMP-1088, a now commercially available inhibitor of *Hs*NMT1/2 with picomolar IC_50_ values.^31^ A similar approach led to the development of IMP-1002, a potent *Pv*NMT inhibitor having an IC_50_ of 3 nM.^30^ Unfortunately, IMP-1002 exhibits low selectivity for *Pv*NMT (selectivity indeces (SIs) of 2.3 and 3.8 over *Hs*NMT1 and *Hs*NMT2, respectively). To identify orthogonal compounds having improved properties, Harupa et al. in 2021 reported inhibition data for a high-throughput screening using the GlaxoSmithKline collection and the Tres Cantos antimalarial kit.^32^ This effort identified 23 new chemical scaffolds showing promising activity and selectivity against *Pf*NMT and *Pv*NMT, including compound **2** (Figure 1B) discussed below in the context of chemical hybridization.

Chemical hybridization and fragment merging techniques, which generate novel small-molecule scaffolds by combining functional groups from different inhibitors and joining separately binding fragments, respectively, have yielded several novel inhibitor series with improved properties.^33−35^ Using the former approach, we recently reported two series of inhibitors of *P. vivax* NMTs (**12** and **30** series) with SIs reaching greater than 250 while maintaining IC_50_ values less than 100 nM.^36^ Excitingly, these compounds also exhibit parasiticidal activity against blood-stage *P. falciparum* at submicromolar concentrations and liver stage *P. vivax* schizonts and hypnozoites at micromolar concentrations.^36^ Cocrystal structures of these compounds with *Hs*NMTs are currently unavailable. However, a high-resolution cocrystal structure with **1** (compound **12b** in ref (36); Figure 1A) revealed novel conformational changes and stable intermolecular contacts that are likely structural determinants of the high selectivity.

In this study, we tested two chemical approaches to investigate hypothesized selective subsites within the inhibitor binding pocket. One approach probes the conformational plasticity and selectivity of a cryptic pocket under Phe226 through a variety of functional group substitutions of the 3-methyl of the trimethylpyrazole tail groups of the series **12** and **30** compounds. A second approach evaluates the effects of polarity and volume variations within the inhibitor core on the local environment, including the conformation of the selective Tyr211 and the arrangement of nearby waters proximal to a water channel.^37,38^ Conformational changes in other structural elements forming the peptide cleft, such as the Ab loop, are studied as well. Inhibition assays demonstrate that chemical alterations of both regions of the scaffold yield a broad range of selectivities, substantiating the associated sites undergoing conformational changes as target regions for inhibitor development efforts. Crystal structures containing two of the highly selective compounds provide a molecular basis for *Pv*NMT inhibition and reveal the underlying binding site architectures that can be further targeted in future inhibitor optimizations.

## Results

### Structure-Guided Design of *Pv*NMT Inhibitors

The structure of the highly selective compound **1** bound to *Pv*NMT served as a template for hybridizations used to explore selective binding at two subsites: the protein-bound waters near the channel and Tyr211 (subsite 1), and the cryptic pocket exposed by the open conformation of Phe226 (subsite 2) (Figure 1A,C). For hybridization probing subsite 2, we sought a second compound having a large tail group that makes specific interactions with NMT and that can be chemically varied. The crystal structure of *Pv*NMT bound to inhibitor **2** (Figure 1B,C), a selective compound from a prior high-throughput screening, illustrates that a morpholinomethanone group can anchor through hydrogen bonding at the Asn365 site with Phe226 in the open conformation.^32^ Both compounds stabilize the selective conformation of Tyr211.^29,32,36^

Docking (see the Experimental Section for details) of a hypothetical compound having a tail group extension (**8g**, described below) suggested binding feasibility via an active site pose that interacts with Ser319, Asn365, and Leu410 (Figure 1D). However, it was unclear if different extensions would be able to bind in the cryptic pocket and form hydrogen bonds with Asn365. It was also unclear whether other inhibitors with a pyridine core A (Figure 1E) would induce the same water structure observed in the complex with **1**, and if a naphthalene group would displace protein-bound waters or instead cause a shift in inhibitor location. In the latter scenario, the peptide binding cleft would likely need to expand to accommodate the additional volume introduced by this moiety.

Physicochemical variations were applied to the core A and tail group extension across three inhibitor series, denoted as 8–10 (Figure 1E, purple and blue segments, respectively). All tested compounds contained a piperazine headgroup and a pyridine core B, (Figure 1E, gray portion). Series **8** compounds have distinct tail group extensions with a phenyl group in core A, with **8b** serving as the parent hybrid scaffold due to its use of a morpholinomethanone extension. For the tail group extension alterations, the ring is removed to provide a heteroatom into a five-atom spacer at position 3 of the pyrazole group. On the other hand, the compound **9** and **10** series replace the core A phenyl group with pyridine and naphthyl groups, respectively.

### Synthesis and Biochemical Studies of *Pv*NMT Inhibitors

**Scheme 1:**
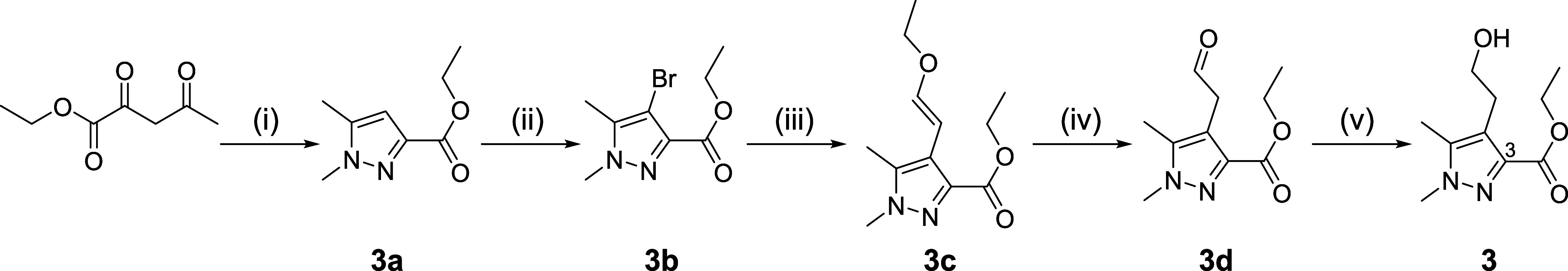
Synthesis of Five-Membered Heterocyclic Block Reagents and conditions: (i) CH_3_NHNH_2_, AcOH, 3 h, rt; (ii) *N*-bromosuccinimide (NBS), 80 °C, 1,2-dichloroethane (DCE), N_2_, 16 h; (iii) *trans*-2-ethoxyvinylboronic acid pinacol ester, K_3_PO_4_, Pd(PPh_3_)_4_, 1,4-dioxane/H_2_O, N_2_, 2 h, 100 °C; (iv) HCl/dioxane, rt, 2 h; (v) NaBH_4_, EtOH, 0 °C-rt, 2 h.

The synthesis of the distinct series involved a stepwise assembly of building blocks through a linear synthesis approach. First, the block of the pyrazole residue (**3**) was constructed, the starting point for introducing the other fragments located at position 3 of the pyrazole, called the tail region. This structural fragment (**3**) was prepared using a five-step synthesis strategy (Scheme 1), with a condensation reaction and Suzuki coupling as key steps.

**Scheme 2:**
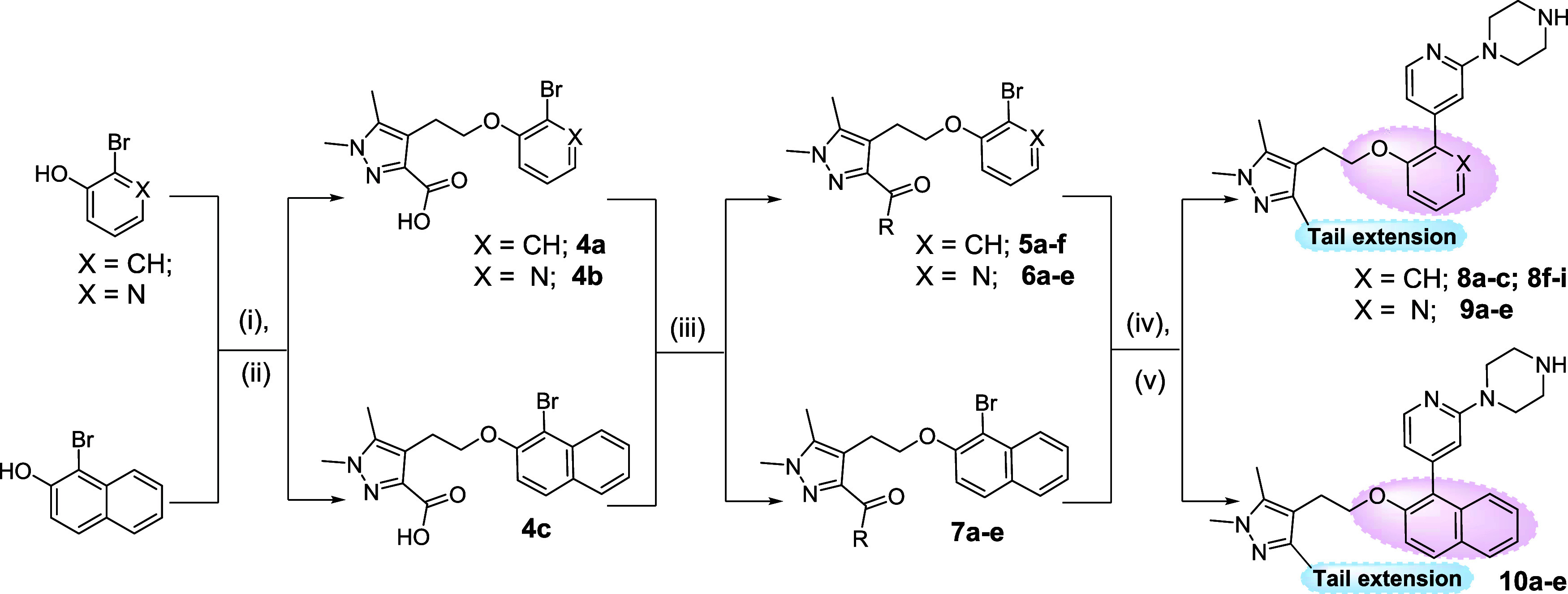
Synthesis of Hybrid Compounds of the Series **8**–**10** Reagents and conditions: (i) **3**, cyanomethyiene tributylphosphorane (CMBP), toluene, 100 °C, 16 h; (ii) KOH, MeOH, 80 °C, 3 h; (iii) HATU, *N,N*-diisopropylethylamine (DIPEA), RNH_2_ or RR′NH, tetrahydrofuran (THF), rt, 12 h; (iv) 2-(4-(*tert*-butoxycarbonyl)piperazin-1-yl)pyridine-4-boronic acid pinacol ester, K_3_PO_4_, Pd(PPh_3_)_4_, 1,4-dioxane/H_2_O, N_2_, 2 h, 100 °C; (v) HCl/dioxane, rt, 2 h.

Scheme 2 summarizes the synthetic route used to prepare the hybrid compounds. The synthesis of compound series **8**, **9**, and **10** commenced with the construction of distinct aryl ether blocks (**4a**–**c**) via a Mitsunobu reaction,^39^ wherein commercially available aryl-hydroxyl compounds were merged with the ethyl 4-(2-hydroxyethyl)-1,5-dimethyl-1*H*-pyrazole-3-carboxylate foundation (block **3**). A two-step procedure was undertaken to incorporate the tail groups starting from these aryl ether blocks (**4a**–**c**). Initially, a hydrolysis reaction was carried out, followed by an amide coupling reaction facilitated by HATU as the coupling reagent. This step led to the creation of intermediary blocks (**5a**–**f**, **6a**–**e**, and **7a**–**e**) using diverse primary or secondary amines. From these aryl ether blocks, hybrid synthesis was accomplished in two steps. A Suzuki cross-coupling reaction was used to couple core A and BOC-piperazine-pyridine moieties together (Figure 1E). Subsequently, Boc-deprotection was executed using 4 M HCl in dioxane, culminating in the successful generation of compound series **8**, **9**, and **10** in overall yields ranging from 60 to 91%. The assessment of NMT activity was performed indirectly, involving the detection of free CoA through the employment of the thiol-reactive probe 7-diethylamino-3-(4′-maleimidylphenyl)-4-methylcoumarin (CPM).^40^

The enzymatic activities of the hybrid compounds **8a**–**c**, **8f**–**i**, and **9a**–**e** are summarized in Table 1. Notably, within series **8**, four compounds displayed high affinity, boasting IC_50_ values below 40 nM. Interestingly, two compounds from this series, namely, those featuring the morpholinomethanone (**8b**) and *N*-2-oxopropylcarboxamide (**8g**) moieties in the tail region, exhibited a notable SI surpassing 31. Compound **9b**, the pyridine equivalent of the parent **8b**, displayed a comparable potency and selectivity. Strikingly, however, replacing the morpholine with acetate caused an increase in selectivity for a pyridine core (**9c**) and a sharp decrease for a phenyl core (**8c**). Inhibitor **9d**, which exhibited the highest selectivity within series **8** and **9**, has a tail group extension isosteric with that of **9c**. The differences in SI, 1.1 versus 50.4-fold change, between the (**8b**, **9b**) and (**8c**, **9c**) pairs, suggesed an interesting dependence of tail group binding on the inhibitor interaction with waters near the channel.

**Table 1:**
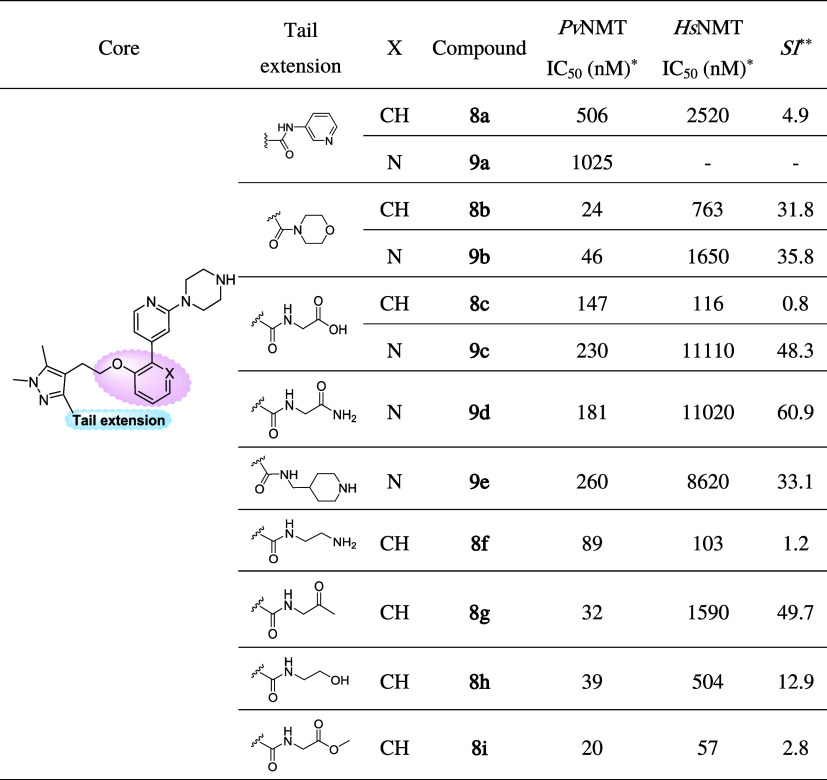
Biochemical Activity of Hybrid Compounds **8a**–**c**, **8f**–**i**, and **9a**–**e**

* *Pv*NMT and *Hs*NMT IC_50_ values are shown as mean values of two or more determinations.

** Enzyme selectivity calculated as *Hs*NMT IC_50_/*Pv*NMT IC_50_ (nM).

The introduction of a naphthol group into core A yielded no improvement in enzymatic inhibition. However, in the case of this series, the affinity enhancement attributed to the inclusion of the morpholinomethanone group in the tail region was accompanied by an appreciable enhancement in selectivity, as outlined in Table 2. Note, while compound **10b** exhibited a reduced affinity for *Pv*NMT when compared to compounds **8b** and **9b**—both of which featured the same moiety in the tail region—it displayed a significantly improved selectivity over *Hs*NMT (SI = 125.3), surpassing the selectivity of all other hybrid compounds detailed in both Tables 1 and 2. However, a noteworthy observation emerged when comparing these outcomes with the most selective compound (**30a**) from the initial generation of *Pv*NMT inhibitors, previously reported by our research group.^36^ The only structural distinction between these compounds lies in the replacement of a methyl group with the morpholinomethanone moiety in the tail region of compound **10b**. While the affinity remained consistent (IC_50_ of 89 vs 91 nM for **10b**), the selectivity was halved (SI = 270 vs 125 for **10b**), underscoring an intriguing interplay between structural modifications and enzymatic behavior.

**Table 2:**
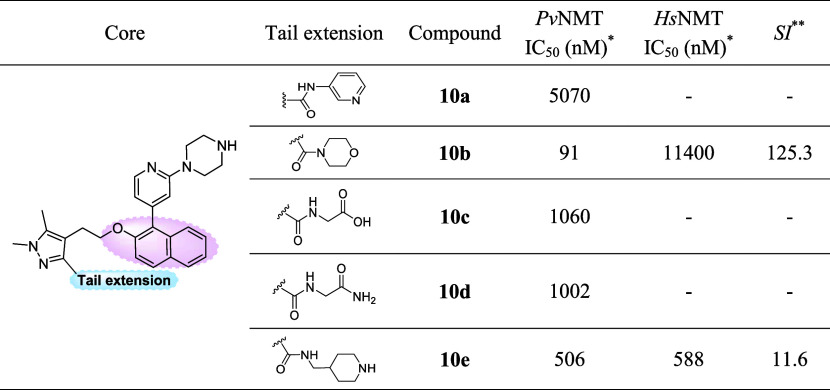
Biochemical activity of hybrid compounds **10a**–**e**

* *Pv*NMT and *Hs*NMT IC_50_ values are shown as mean values of two or more determinations.

** Enzyme selectivity calculated as *Hs*NMT IC_50_/*Pv*NMT IC_50_ (nM).

### Selective Architectures Exhibited by **9c**- and **10b**-Bound *Pv*NMT

The cocrystals of **9c** and **10b** with *Pv*NMT and MyrCoA both belong to the same space group and have similar unit cell constants to those of the majority of *Pv*NMT crystal structures in the PDB. The resolutions of the refined models are 1.97 and 2.43 Å, respectively. The asymmetric units contain three copies of the ternary complexes. Continuous chains were built into electron density, beginning at either Asp27, the first residue of the truncated ORF cloned, or the preceding proline. Additionally, all atoms of MyrCoA, **9c**, and **10b** were modeled. The electron density, however, is weak for the tail group extension of **9c** and indicates multiple conformations. Two copies of **9c** were built into each active site.

**Figure 2:**
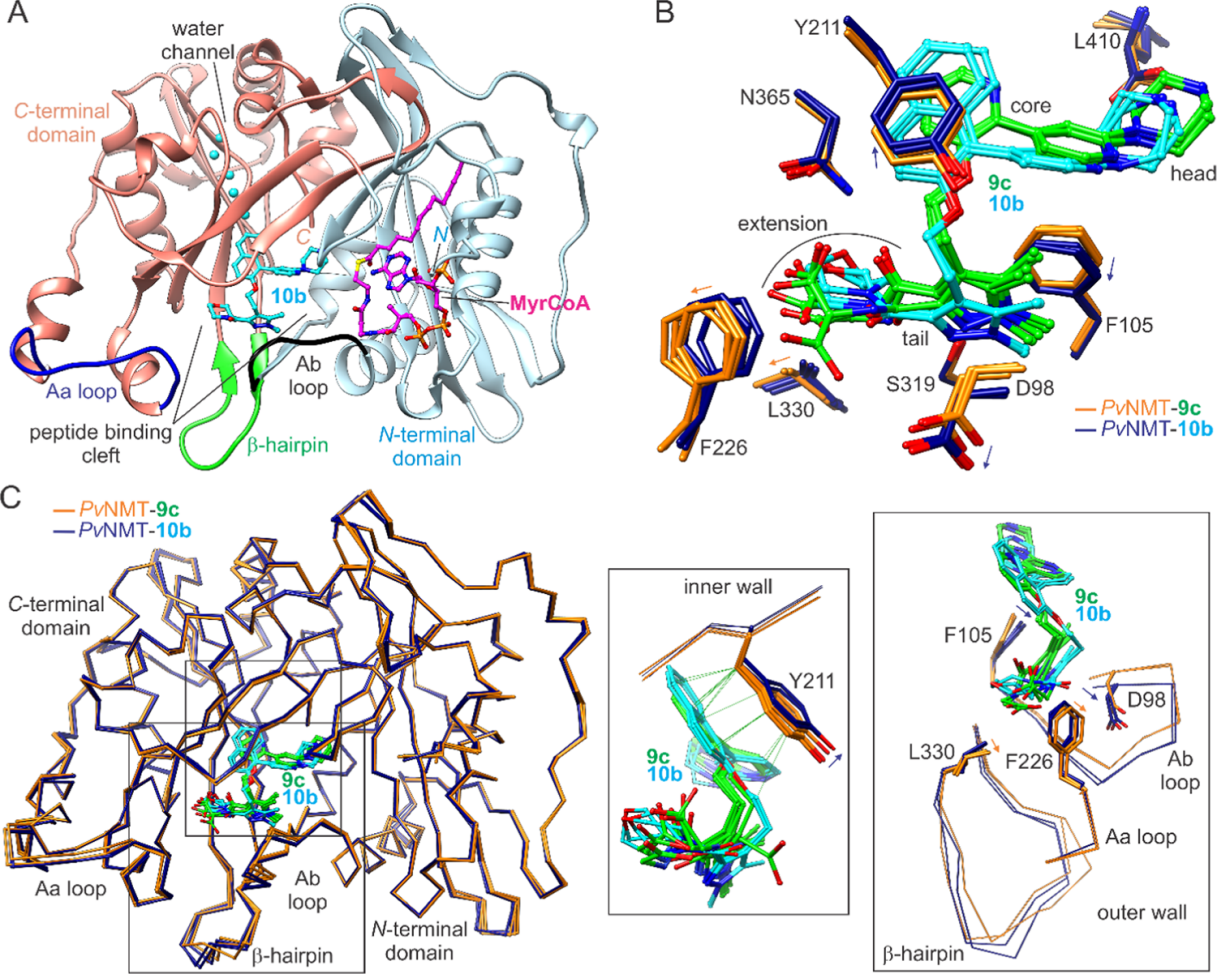
Global conformations and inhibitor binding sites of **9c**- and **10b**-bound complexes. (A) Compound **10b** binds in common *Pv*NMT/*Hs*NMT1 inhibitor binding site within peptide binding cleft. Outer cleft wall structural elements and water channel are highlighted. (B) Compounds **9c** and **10b** adopt canonical conformation but cause structural perturbations of differing extent at wall residues (indicated with arrows). (C) Cα trace superpositions reveal inner and outer wall mainchain conformational variability. Insets provide close-up views of wall sections. Close hypothetical van der Waals contacts (≤3.2 Å) are indicated using green lines.

The inhibitor binding site is located within the peptide binding cleft formed by the *N*- and *C*-terminal domains (Figure 2A). The inner wall of the cleft is formed near the interface of the composite β-sheet of the two domains, whereas the outer wall comprises the previously delineated “Ab loop” (residues 95–102), a β-hairpin extending out from the core β-sheet (residues 319–330), and the “Aa loop”, so named after the Ab loop on a similar basis of flanking secondary structural elements (residues 224–234). The binding site also includes several protein-bound waters next to the inner edge of a water channel within the *C*-terminal domain.

Both inhibitors adopt the canonical pose,^23,28−30,36^ i.e., they straddle the side chain of Phe105, with the headgroup situated near the Leu410 carboxylate and the tail group near the Ser319 hydroxyl group (Figure 2B). The binding sites are formed by similar sets of residues, although small but significant variations are observed between the structures that are reflected in deviations in mainchain conformations of the inner and outer walls (Figure 2C). Notably, chain C in both crystal structures inserts into the peptide binding cleft via a novel conformational change, detailed in Figure S1. Tyr211 adopts the selective conformation described previously.^28−30^ However, the bulkiness of **10b** causes an additional “vertical” displacement that relieves hypothetical clashes revealed through structural superpositions. There is an accompanying rotation of Phe105. The displacement of the pyrazole group of **10b** causes a more significant outward shift of the Ab loop (observed at Asp98) and weakens the hydrogen bonding with Ser319. In the complex with **9c**, this hydrogen bond is also weakened, but through a different mechanism related to the dynamics in the 3-methyl extension, which are associated with a slightly greater binding site expansion at the Aa loop and neighboring β-hairpin (observed at Phe226 and Leu330).

**Figure 3:**
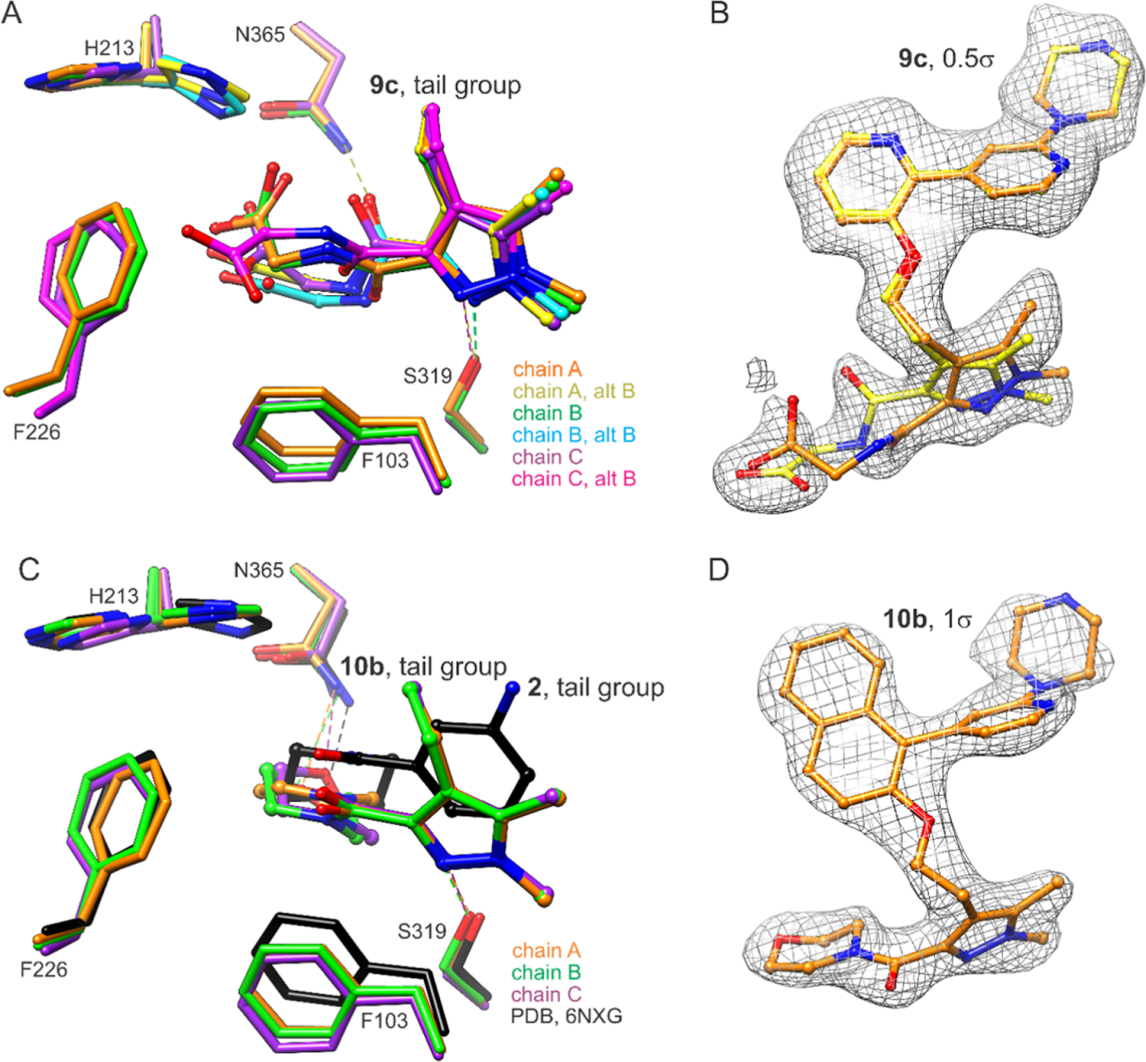
Multistate binding of tail group extensions. (A) Compound **9c** tail group binds unstably with 3-methyl extension able to insert into the pocket formed by the open conformation of Phe226. Two ligand conformations are modeled in each chain. Potential hydrogen bonds are indicated with dashed lines. (B) 2*Fo*-*Fc* map around **9c**, chain A, after refinement with ligand omitted, contoured at approximately 0.5σ to reveal structural features of the extension. (C) Compound **10b** tail group and morpholinomethanone extension bind with much higher order. The morpholine group exhibits varied conformations, but its O1 atom consistently engages the Asn365 side chain through hydrogen bonding. The corresponding hydrogen bond with **2** occurs via ring inversion. (D) 2*Fo*-*Fc* map around **10b**, chain B, after refinement with ligand omitted, contoured at 1σ.

Both extensions bind within the cryptic pocket formed when Phe226 is in the open conformation. However, the tail group and extension of **9c** are unstably bound, with six conformations modeled (Figure 3A,B). Of these, two consistent conformers (observed in more than one chain) have the amide carbonyl directed either toward the side chain of Asn365 or that of Ser319. The carboxylate does not appear to form hydrogen bonds with the protein. In contrast, the tail group and extension of **10b** are more stably bound. The morpholinomethanone group anchors to Asn365 via hydrogen bonding with the ether oxygen in all asymmetric unit chains, despite conformational variability observed within the morpholine ring itself (Figure 3C,D). By comparison, this hydrogen bond is formed by **2** via ring inversion.^32^ In the presence of the **9c** dynamics, the His213 side chain adopts both inwardly and outwardly pointing rotamers (A and B, respectively), but considering all three asymmetric unit copies, samples the B state with higher occupancy. The higher occupancy of the B rotamer is even more pronounced in the **10b**-bound structure.

**Figure 4:**
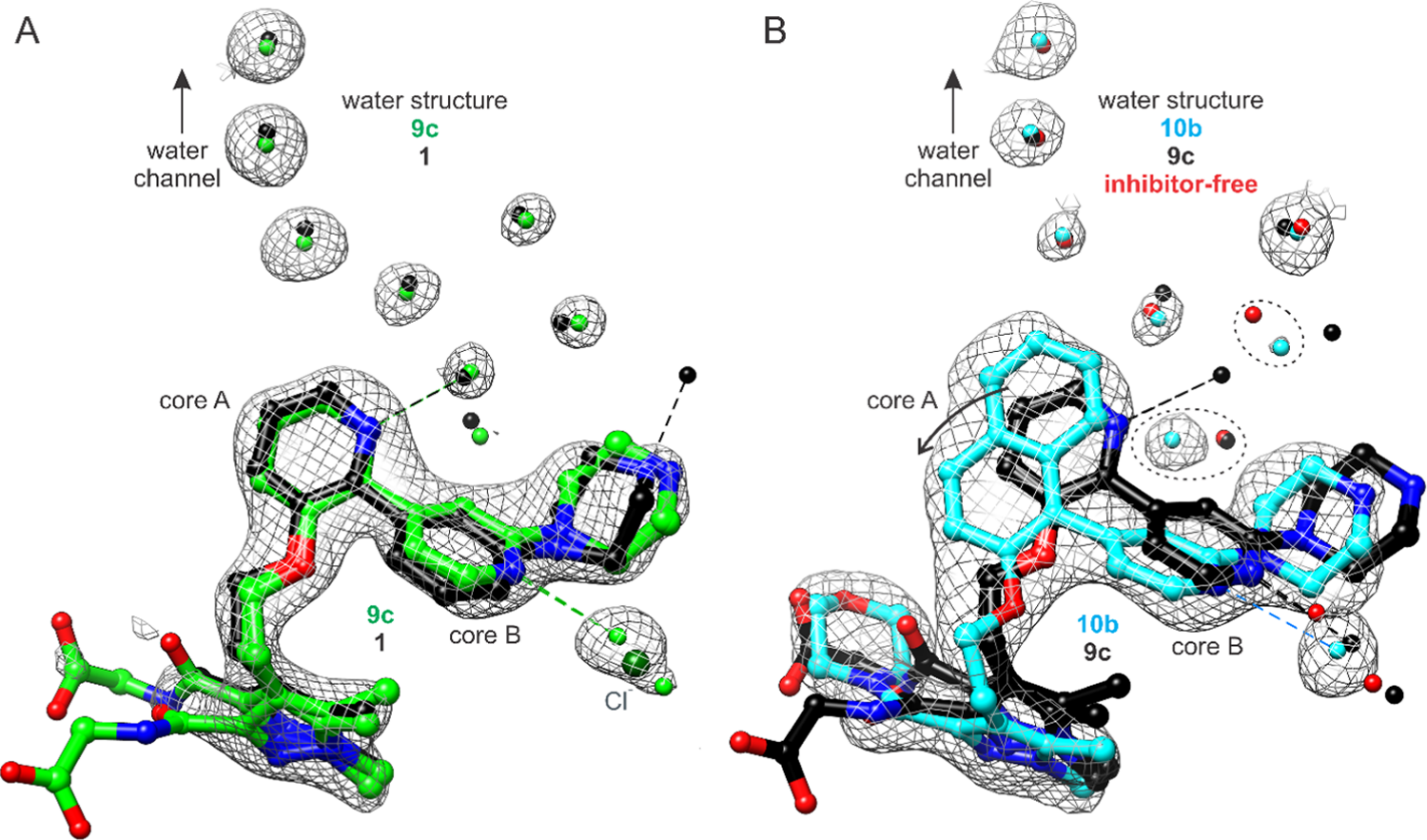
Water arrangements near core groups A and B. (A) Configuration observed in *Pv*NMT-**9c** complex resembles that in complex with **1** (arrangement in chain C is shown). (B) Configuration in *Pv*NMT-**10b** complex involves one less water, similar to that of inhibitor-free enzyme (PDB 4B10(29)) (arrangement in chain B is shown). In both panels, a water was assigned to the peak nearest the nitrogen atom of the core B pyridine (see main text discussion). 2*Fo*-*Fc* electron density maps are shown at a contour level of approximately 1σ.

In addition to the aforementioned conformational changes at Tyr211 and Phe105 caused in part by core group differences, the physicochemical changes introduced in the core A group perturb the local water structure at the leading edge of the water channel. The pyridine of **9c** draws a water molecule closer through hydrogen bonding thereby enabling the incorporation of an extra water (Figure 4A). The arrangement superimposes closely with that observed in the complex with **1** (nearer to the headgroup, however, one or two fewer waters can be modeled due to the closer proximity of the *N4* ammonium of **9c** to Asn161). Despite its volume, the naphthalene group does not displace the waters that extend the channel to the Tyr315 side chain and Leu409 carbonyl sites, although differences in the water structure are observed between the three chains and the occupancies differ (Figure 4B). The implication is that the core A phenyl group, in comparison to the core A pyridine of **9c**, glides beneath the side chain of Tyr211. For both complexes, the peak nearest the nitrogen of the core B pyridine was tentatively assigned as one water molecule or a dynamic water visiting two ordered sites, instead of chloride.

**Figure 5:**
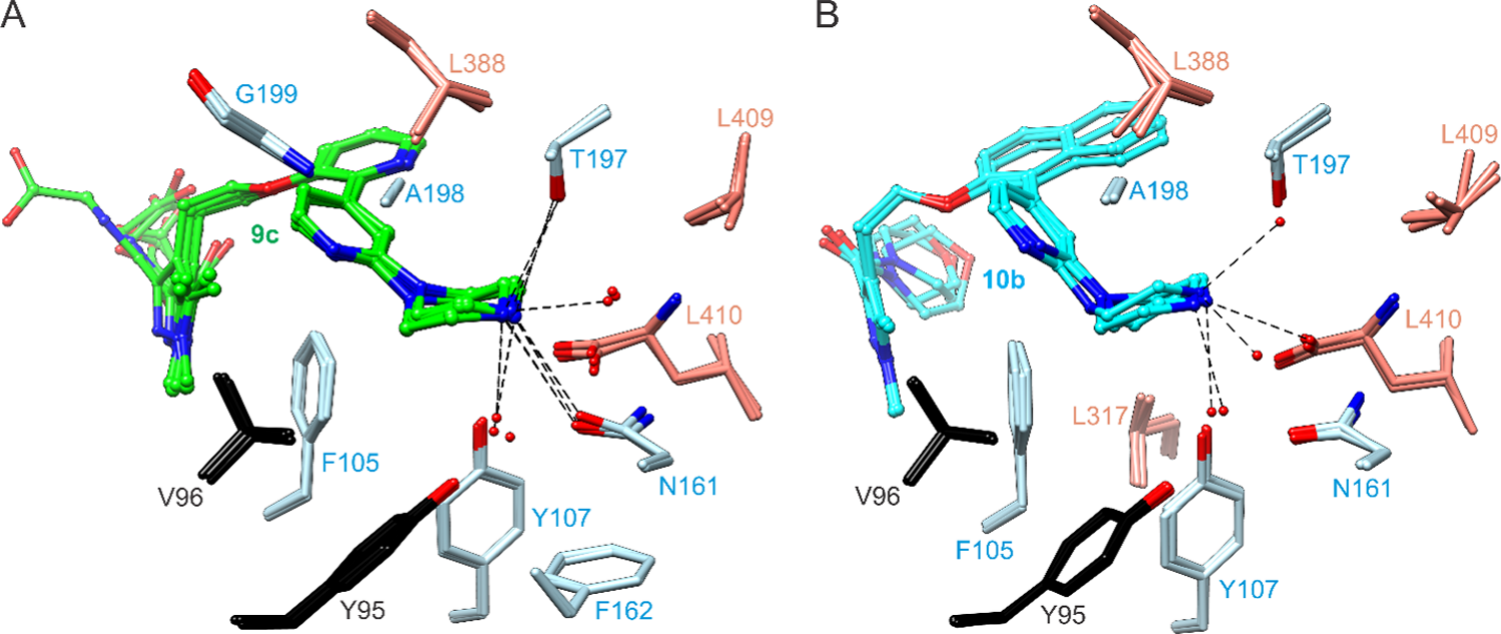
Headgroup (piperazine) binding site differences. (A) *N4* ammonium of **9c** is oriented more closely to polar sites in Asn161 and Thr197 side chains. (B) Compound **10b***N*4 ammonium interacts more closely with *C*-terminal carboxylate. Hydrogen and ionic bonds (dashed lines) are drawn based on a 3.5 Å cutoff.

Although the headgroup was not varied in this study, significant structural differences occurred in the piperazine binding site. Within the complex with **9c**, the *N*4 ammonium interacts electrostatically with the side chains of Asn161 and Thr197 and the carboxylate of Leu410 but is situated in closer proximity to the side chains (Figure 5A). In the complex with **10b**, it is displaced by 1–2 Å and instead aligns more closely with the carboxylate (Figure 5B). These differences are partly responsible for the conformational changes observed in Phe105, and the differential placements of the piperazine groups are associated with slight (sub-Angstrom) shifts in the location of the MyrCoA sulfur atom.

## Discussion and Conclusions

The design of inhibitors that bind selectively to *Pv*NMT over the *Hs*NMTs must leverage differences in conformational properties of these enzymes owing to the chemical identity of their inhibitor binding sites. Both **9c** and **10b** are bulky inhibitors that require *Pv*NMT to undergo major conformational changes to bind the observed poses. Both inhibitors include the piperazine headgroup containing a four-atom spacer to the ammonium ion and have sizeable extensions at the 3-position of their pyrazole tail groups. Compound **10b** adds additional volume in the core A group via the naphthalene moiety.

**Figure 6:**
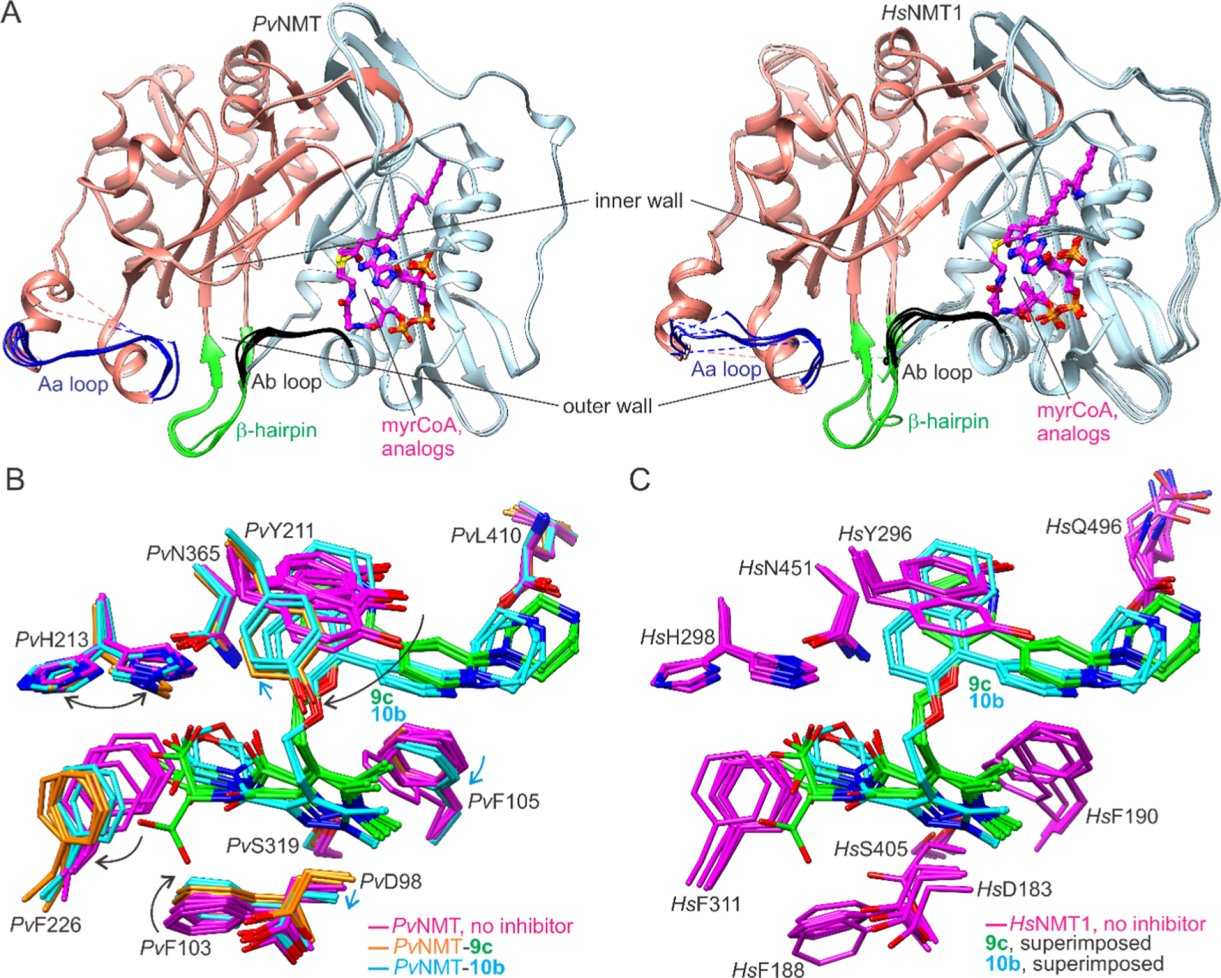
Conformational changes required for binding **9c** and **10b**. (A) Intrinsic flexibility of the peptide binding clefts of *Pv*NMT (left, PDB codes 2YNC(23) and 4B10(29)) and *Hs*NMT1 (right, PDB codes 3IU1, 5NPQ, and 5UUT(42)), in their inhibitor-free forms. (B) Changes observed in particular residues of *Pv*NMT upon inhibitor binding indicated schematically with arrows. (C) Corresponding residues in inhibitor-free *Hs*NMT1 adopt similar conformations to those in inhibitor-free *Pv*NMT shown in (B). Hypothetical steric clashes with **9c** and **10b** are thus inferred through close contacts and interpenetrating chains after structural superpositions.

The peptide binding clefts of *Pv*NMT and *Hs*NMT show considerable conformational variability in crystal structures (Figure 6A). However, the effect of conformational plasticity differences on selectivity is an open question because the relative conformational free energy landscapes are unknown, and similar ensembles of ground state conformations are observed in crystal structures (Figure 6B,C). Previous work demonstrated via mutagenesis, inhibition assays, and structural analyses for particular inhibitors how differences in plasticity in the inner wall can result in selective binding to *Pv*NMT.^29^ This occurs through the exaggerated rotation of the Tyr211 side chain beyond what is observed in unbound and peptide-bound states. Both **9c** and **10b** bind with Tyr211 in this rotameric state, which is expected to contribute to the observed selectivities. However, the magnitude of the contribution is unclear; some poorly selective *Pv*NMT inhibitors are known to stabilize this conformation,^30^ and the human enzyme was previously shown to bind particular inhibitors in this state with picomolar affinities.^31^

Our interest in probing additional subsites for selective binding was motivated by our recent work using the **12** and **30** series compounds, which revealed additional subsite plasticity in *Pv*NMT, both conformational and configurational. In the complex with **1** (previously **12b**), a novel conformation was observed in the Aa loop, and a new molecular arrangement of waters and a chloride site were observed near the core A and B groups, respectively.

Based on the conformational change in the Aa loop observed in the **1**-bound complex, we probed this region via appending various functional groups to the 3-methyl of the pyrazole tail group, thereby testing the effect of expanding the binding site in this region on selectivity. The tail group extension dependence of the selectivity revealed by the SI data in Tables 1 and 2 supports our hypothesis that this pocket can be selective. Although the crystal structures demonstrate that the extensions of **9c** and **10b** are able to bury beneath Phe226, it remains to be determined whether burial is feasible for all of the extensions studied. An inability to do so might explain some of the lower selectivity data. In comparison to the much less selective IMP-1002, which lacks a 3-methyl extension, the changes at the cryptic pocket residues His213, Phe226, and Leu330 are more pronounced. Furthermore, as shown in Table 1, probing the water channel via substitution of the core A moiety with a phenyl group, which also occurs in IMP-1002, yields lower selectivities, with the altered arrangement of waters observed in the **9c**-bound structure providing a possible molecular basis.

Structural differences between the complexes with **9c** and **10b** were examined to further dissect the binding site in light of the selectivity differences (SIs of 48.3 and 125.3, respectively). In the latter complex, the Ab loop is pushed farther outward due to the increased size and positioning of the core and tail groups. However, the conformational change in Phe226 in the Aa loop (and additionally Leu330 in the adjacent β-hairpin) in response to binding the dynamic extension of **9c** is slightly greater than in the complex with **10b**. Similar conformational changes in the Ab loop of *Hs*NMT1 were observed previously using two *Leishmania major* NMT inhibitors^41^ and in the Aa loop of *Pv*NMT using a quinoline inhibitor.^27^ In the former case, the inhibitors selectively bind *Lm*NMT, although the affinities for the human enzyme reach the nanomolar range; the selectivities for *Pv*NMT are unknown. The quinoline inhibitor is able to insert its side chain into the pocket beneath Phe226 and is also selective, but the side chain is flexible, and the SI reached only 4 for *Pv*NMT over *Hs*NMT1 (>21 over *Hs*NMT2).

One hypothesis to explain the selectivity deviations relates to the difference in binding stabilities of the tail groups and their extensions, i.e., that in the case of **9c**, the affinity derives more from interactions in other regions of the cleft where there are fewer differences in plasticity between the two enzymes. A second hypothesis is that the bulkiness of **10b** introduces a number of additional binding site perturbations that have a compounded effect on selectivity. These include the more extensive movements observed at the inner wall (rise of Tyr211), Ab loop (outward displacement of Asp98), and “pommel horse” residue (rotation of Phe105) (Figure 6B).

**Figure 7:**
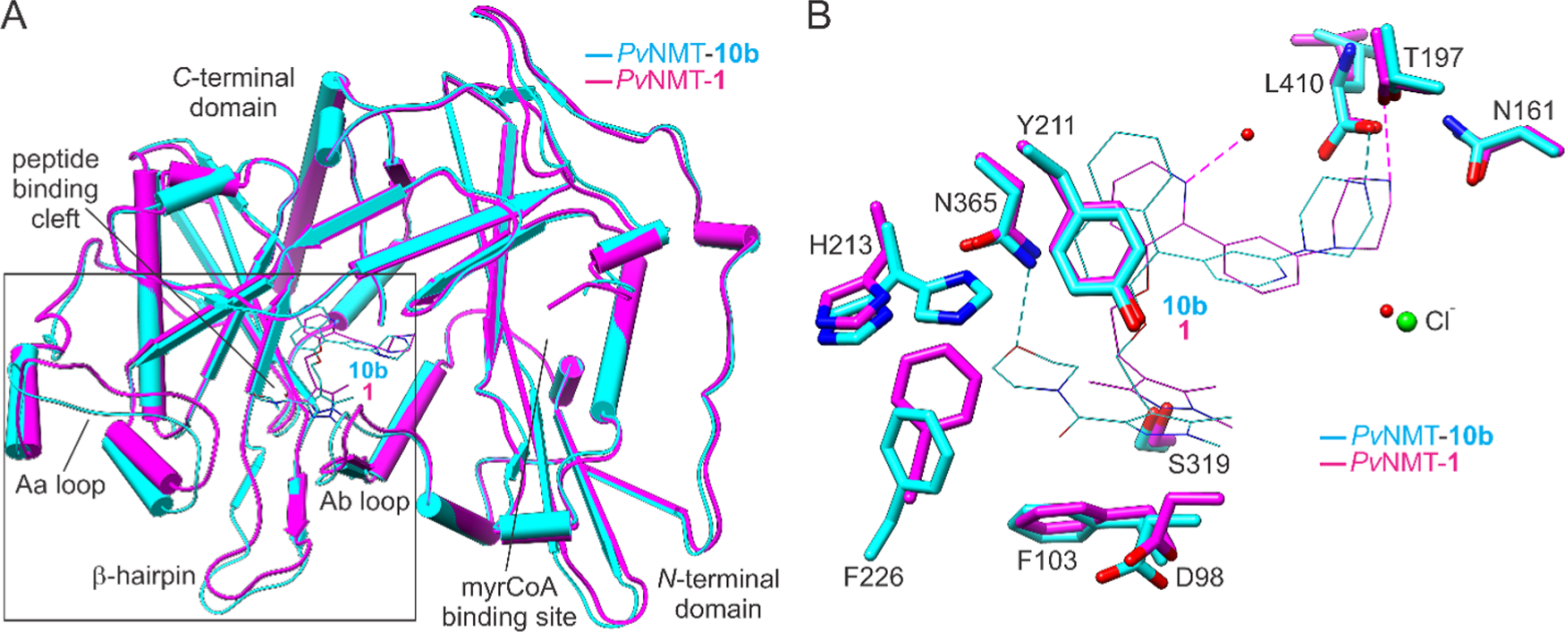
Conformational plasticity of *Pv*NMT: selective binding feasible through distinct architectures. (A) *Pv*NMT adopts different peptide binding cleft conformations (boxed) to tightly bind **10b** and **1**, which differ considerably physicochemically in their core and tail groups. (B) Close-up view of selected residues highlighting conformational differences and unique interactions.

Comparison of the **10b** and **1** binding sites demonstrates the remarkable versatility of the peptide binding cleft of *Pv*NMT to conform to the very distinct core and tail group structures, which is likely not readily recapitulated by the human enzyme on the basis of the large increases in IC_50_ values (Figure 7). Compound **1** also contains the bulky piperazine headgroup but lacks an extension at the pyrazole 3-methyl position. Its piperazine group is misaligned with the *C*-terminal carboxylate, in this case interacting most closely with Thr197, and a novel chloride site is formed with the core B phenyl group. Instead of being displaced, Phe226 is drawn inward toward the 3-methyl to an unprecedented degree (Leu330 following suit), which is accommodated by the B rotamer of His213.^36^ Thus, an important conceptual advance is that “jamming” the cryptic pocket with more atoms to expand the binding site as an approach to enhance selectivity, while effective, is thus far less efficacious than stabilizing the **1**-bound conformation. On this point, it is also noteworthy that the most selective compound known presently, **30a** from our previous study (SI 270),^36^ is an abridged derivative of **10b** lacking an extension.

In this study, we have expanded our understanding of how inhibitor selectivity can be achieved in the context of high sequence similarity of the inhibitor binding sites. The insights gained from the SI data and crystal structure comparisons help prioritize target subsites and conformational changes for future studies improving inhibitor selectivity.

## Experimental Section

### General Experimental Information for Synthesis and Compound Characterization

General reagents and solvents for synthesizing compounds were purchased from commercial sources and used as supplied unless otherwise stated.

Purification by flash column chromatography was performed on a Selekt (Biotage, U.K.) automated instrument with Sfär KP-amino D or Sfär silica D cartridges (Biotage, U.K.) (mobile phase consisting of pentane (solvent A) and ethyl acetate (solvent B)) or by reversed-phase flash column chromatography performed on an Isolera (Biotage, U.K.) automated instrument with Sfär C18 D cartridges (Biotage, U.K.) (mobile phase consisting of water (solvent A) and acetonitrile (solvent B)). The standard gradient consisted of *x*% solvent B for one column volume, *x*% to *y*% B for 10 column volumes, and then *y*% B for 2 column volumes. *x* and *y* are defined in the characterization section of the compound of interest.

All NMR spectra (^1^H and ^13^C) were recorded on a Varian 400 MHz spectrometer at 25 °C. Samples were dissolved (0.5 mL) in deuterated chloroform (CDCl_3_), methanol (CD_3_OD), or dimethyl sulfoxide (DMSO-*d*_6_). The residual solvent peaks specific to the deuterated solvent were used as an internal reference; CDCl_3_: 7.26 ppm (^1^H NMR) and 77.20 ppm (^13^C NMR); CD_3_OD: 3.31 ppm (^1^H NMR) and 49.00 ppm (^13^C NMR); DMSO-*d*_6_: 2.50 ppm (^1^H NMR) and 39.52 ppm (^13^C NMR). Data are presented as follows: chemical shift in ppm, multiplicity (br = broad, s = singlet, d = doublet, dd = doublet of doublets, ddd = doublet of doublets of doublets, t = triplet, q = quartet, m = multiplet), coupling constants in Hz and integration.

The purity of NMT inhibitors was in all cases equal to or greater than 95% and was performed on an analytical HPLC machine (Waters 2690 Separations Module; Atlantis T3, 5 μm column, 4.6 mm × 250 mm; H_2_O/ACN (0.1% TFA)). High-resolution mass spectra (HRMS) were recorded on an Agilent 1290 infinity LC system in tandem with an Agilent 6520 Accurate Mass Q-TOF spectrometer.

### Detailed Synthetic Procedure and Characterization of Compounds

#### General Procedure A—Mitsunobu Reaction

A solution of the alcohol (1 equiv) in toluene (10 mL) was reacted with selected aryl (1.25 equiv) and cyanomethyiene tributylphosphorane (CMBP) (1.5 equiv) at 100 °C for 16 h. The reaction mixture was then diluted with ethyl acetate (50 mL) and washed with water (1 × 25 mL) and brine (2 × 25 mL). The organic phase was separated, dried over Na_2_SO_4_, filtered, and concentrated under reduced pressure. The crude product was purified by flash column chromatography eluting with a gradient of pentane/EtOAc or by reversed-phase chromatography eluting with a gradient H_2_O/ACN.

#### General Procedure B—Hydrolysis

To a solution of selected ethyl-ester (1 equiv) in methanol (10 mL) at room temperature was added potassium hydroxide (2 equiv) in one portion. The reaction mixture was warmed to 100 °C and then stirred for 3 h. The resulting solution was cooled to room temperature after which the volatile solvent was evaporated. The residue was acidified with 10% hydrochloric acid and then filtered using water and hexane. The filtrated solid was dried under vacuum to obtain the title compound.

#### General Procedure C—Amide Coupling

A solution of respective carboxylic acid (1 equiv) in dry tetrahydrofuran (5 mL) was treated with 1-[Bis(dimethylamino)methylene]-1*H*-1,2,3-triazolo[4,5-*b*]pyridinium 3-oxid hexafluorophosphate (HATU) (1.5 equiv) and *N*,*N*-diisopropylethylamine (3 equiv) followed by the corresponding primary or secondary amine (1.5 equiv). The reaction mixture was stirred at room temperature overnight under N_2_. The reaction mixture was then diluted with ethyl acetate (25 mL) and washed with water (1 × 25 mL) and brine (2 × 25 mL). The organic phase was dried over Na_2_SO_4_ and concentrated under reduced pressure. The crude product was purified by flash column chromatography eluting with a gradient of pentane/EtOAc or by reversed-phase chromatography eluting with a gradient H_2_O/ACN to provide the desired amide.

#### General Procedure D—Suzuki Cross-Coupling Reaction

A solution of selected aryl-bromide (1 equiv) was dissolved in 1,4-dioxane (10 mL) in a microwave vial (10–20 mL) and treated with the corresponding aryl boronic acid pinacol ester (1.5 equiv) and tetrakis(triphenylphosphine) palladium(0) (0.05 equiv), followed by a solution of potassium phosphate (2.7 equiv) in water (3 mL) under N_2_. The reaction mixture was heated at 100 °C for 1–3 h. The solution was cooled to room temperature and evaporated under reduced pressure. The residue was partitioned between EtOAc (20 mL) and saturated sodium bicarbonate solution (20 mL). The organic phase was dried over Na_2_SO_4_, filtered, and concentrated under reduced pressure. The crude product was purified by flash column chromatography eluting with a gradient of pentane/EtOAc or by reversed-phase chromatography eluting with a gradient of H_2_O/ACN.

#### General Procedure E—Boc-Deprotection

The Boc-protected amine was dissolved in dioxane (1 mL) and treated with 4 M HCl in dioxane (2 equiv). The reaction mixture was stirred at room temperature for 2 h. All volatiles were removed under reduced pressure, and the product was triturated with ether and dichloromethane (DCM), redissolved in water, and freeze-dried to afford the desired compound.

##### Ethyl 4-(2-Hydroxyethyl)-1,5-dimethyl-1*H*-pyrazole-3-carboxylate (**3**)

Ethyl 1,5-dimethyl-1*H*-pyrazole-3-carboxylate (**3a**) was prepared as described previously.^43^ Ethyl 4-bromo-1,5-dimethyl-1*H*-pyrazole-3-carboxylate (**3b**), ethyl (*E*)-4-(2-ethoxyvinyl)-1,5-dimethyl-1*H*-pyrazole-3-carboxylate (**3c**), ethyl 1,5-dimethyl-4-(2-oxoethyl)-1*H*-pyrazole-3-carboxylate (**3d**), and ethyl 4-(2-hydroxyethyl)-1,5-dimethyl-1*H*-pyrazole-3-carboxylate (**3**) were prepared as described previously.^43−45^

##### 4-(2-(2-Bromophenoxy)ethyl)-1,5-dimethyl-1*H*-pyrazole-3-carboxylic Acid (**4a**)

Following General Procedure A, **3** (230 mg, 1.1 mmol) was reacted with 2-bromophenol (235 mg, 1.35 mmol) to afford 306 mg (77%) of ethyl 4-(2-(2-bromophenoxy)ethyl)-1,5-dimethyl-1*H*-pyrazole-3-carboxylate. ^1^H NMR (CDCl_3_, 400 MHz, δ, ppm) 7.49 (1H, dd, *J* = 7.9, 1.6 Hz), 7.21 (1H, ddd, *J* = 8.3, 7.4, 1.4 Hz), 6.89 (1H, dd, *J* = 8.3, 1.4 Hz), 6.78 (1H, td, *J* = 7.4, 1.4 Hz), 4.39 (2H, q, *J* = 7.1 Hz), 4.19 (2H, t, *J* = 6.1 Hz), 3.85 (3H, s), 3.19 (2H, t, *J* = 6.1 Hz), 2.35 (3H, s), 1.40 (6H, t, *J* = 7.1 Hz). Next, following General Procedure B, the intermediary compound (305 mg, 0.83 mmol) was reacted with KOH (91 mg, 1.66 mmol) to afford 273 mg (97%) of the title compound as a white solid. ^1^H NMR (CDCl_3_, 400 MHz, δ, ppm) 12.49 (1H, brs), 7.54 (1H, d, *J* = 7.9 Hz), 7.29 (1H, t, *J* = 7.8 Hz), 7.12 (1H, d, *J* = 7.3 Hz), 6.86 (1H, t, *J* = 7.6 Hz), 4.10 (2H, t, *J* = 6.7 Hz), 3.77 (3H, s), 3.06 (2H, t, *J* = 6.8 Hz), 2.27 (3H, s).

##### 4-(2-((2-Bromopyridin-3-yl)oxy)ethyl)-1,5-dimethyl-1*H*-pyrazole-3-carboxylic Acid (**4b**)

Following General Procedure A, **3** (400 mg, 1.88 mmol) was reacted with 2-bromo-3-hydroxypyridine (410 mg, 2.35 mmol) to afford 400 mg (57.6%) of ethyl 4-(2-((2-bromopyridin-3-yl)oxy)ethyl)-1,5-dimethyl-1*H*-pyrazole-3-carboxylate. ^1^H NMR (CDCl_3_, 400 MHz, δ, ppm) 7.49 (1H, dd, *J* = 7.9, 1.6 Hz), 7.21 (1H, ddd, *J* = 8.3, 7.4, 1.4 Hz), 6.89 (1H, dd, *J* = 8.3, 1.4 Hz), 6.78 (1H, td, *J* = 7.4, 1.4 Hz), 4.39 (2H, q, *J* = 7.1 Hz), 4.19 (2H, t, *J* = 6.1 Hz), 3.85 (3H, s), 3.19 (2H, t, *J* = 6.1 Hz), 2.35 (3H, s), 1.40 (6H, t, *J* = 7.1 Hz). Next, following General Procedure B, the intermediary compound (430 mg, 1.16 mmol) was reacted with KOH (129 mg, 2.33 mmol) to afford 250 mg (62%) of the title compound as a white solid. ^1^H NMR (CD_3_OD, 400 MHz, δ, ppm) 7.90 (1H, dd, *J* = 4.7, 1.5 Hz), 7.49 (1H, dd, *J* = 8.3, 1.5 Hz), 7.35 (1H, dd, *J* = 8.2, 4.7 Hz), 4.25 (2H, t, *J* = 6.4 Hz), 3.83 (3H, s), 3.20 (2H, t, *J* = 6.4 Hz), 2.36 (3H, s).

##### 4-(2-((1-Bromonaphthalen-2-yl)oxy)ethyl)-1,5-dimethyl-1*H*-pyrazole-3-carboxylic Acid (**4c**)

Following General Procedure A, **3** (210 mg, 0.98 mmol) was reacted with 1-bromo-2-naphthol (358 mg, 1.48 mmol) to afford 290 mg (70%) of ethyl 4-(2-((1-bromonaphthalen-2-yl)oxy)ethyl)-1,5-dimethyl-1*H*-pyrazole-3-carboxylate. ^1^H NMR (DMSO-*d*_6_, 400 MHz, δ, ppm) 8.06 (1H, d, *J* = 8.4 Hz), 7.97 (1H, d, *J* = 9.0 Hz), 7.92 (1H, d, *J* = 8.2 Hz), 7.62 (1H, ddd, *J* = 8.4, 6.8, 1.2 Hz), 7.52 (1H, d, *J* = 9.1 Hz), 7.43 (1H, ddd, *J* = 8.4, 6.9, 1.2 Hz), 4.29 (2H, t, *J* = 6.6 Hz), 4.23 (2H, q, *J* = 7.1 Hz), 3.80 (3H, s), 3.13 (2H, t, *J* = 6.6 Hz), 2.29 (3H, s), 1.26 (3H, t, *J* = 7.1 Hz). Next, following General Procedure B, the intermediary compound (295 mg, 0.70 mmol) was reacted with KOH (78 mg, 1.41 mmol) to afford 180 mg (65%) of the title compound as a white solid. ^1^H NMR (DMSO-*d*_6_, 400 MHz, δ, ppm) 12.51 (1H, brs), 8.07 (1H, d, *J* = 8.6 Hz), 7.96 (1H, d, *J* = 9.2 Hz), 7.92 (1H, d, *J* = 8.2 Hz), 7.62 (1H, brt, *J* = 7.5 Hz), 7.53 (1H, d, *J* = 9.1 Hz), 7.44 (1H, brt, *J* = 7.4 Hz), 4.28 (2H, t, *J* = 6.7 Hz), 3.78 (3H, s), 3.12 (2H, t, *J* = 6.6 Hz), 2.28 (3H, s).

##### 4-(2-(2-Bromophenoxy)ethyl)-1,5-dimethyl-*N*-(pyridin-3-yl)-1*H*-pyrazole-3-carboxamide (**5a**)

Following General Procedure C, **4a** (70 mg, 0.20 mmol) was reacted with 3-aminopyridine (29 mg, 0.31 mmol). The crude was purified by flash column chromatography (pentane/EtOAc in gradient) to afford 70 mg (81%) of the title compound as a pale yellow solid. ^1^H NMR (400 MHz, DMSO-*d*_6_, δ, ppm) 8.97 (1H, d, *J* = 2.7 Hz), 8.26 (1H, dd, *J* = 4.7, 1.5 Hz), 8.23 (1H, ddd, *J* = 8.4, 2.7, 1.5 Hz), 7.54 (1H, dd, *J* = 7.9, 1.5 Hz), 7.33 (1H, ddd, *J* = 8.4, 4.8, 0.7 Hz), 7.32–7.27 (1H, m), 7.13 (1H, dd, *J* = 8.3, 1.3 Hz), 6.87–6.82 (1H, m), 5.27 (1H, brs), 4.17 (2H, t, *J* = 6.6 Hz), 3.86 (3H, s), 3.14 (2H, t, *J* = 6.6 Hz), 2.32 (3H, s).

##### (4-(2-(2-Bromophenoxy)ethyl)-1,5-dimethyl-1*H*-pyrazol-3-yl)(morpholino)methanone (**5b**)

Following General Procedure C, **4a** (65 mg, 0.19 mmol) was reacted with morpholine (25 mg, 0.28 mmol). The crude was purified by flash column chromatography (pentane/EtOAc in gradient) to afford 20 mg (46%) of the title compound as a pale yellow solid. ^1^H NMR (CDCl_3_, 400 MHz, δ, ppm) 7.47 (1H, dd, *J* = 7.9, 1.6 Hz), 7.19 (1H, ddd, *J* = 8.3, 7.4, 1.6 Hz), 6.90 (1H, d, *J* = 8.2 Hz), 6.80–6.74 (1H, m), 4.21 (2H, t, *J* = 6.0 Hz), 3.92 (2H, brs), 3.75 (3H, s), 3.73 (4H, brs), 3.67–3.61 (2H, m), 3.05 (2H, t, *J* = 6.1 Hz), 2.32 (3H, s).

##### Methyl (4-(2-(2-Bromophenoxy)ethyl)-1,5-dimethyl-1*H*-pyrazole-3-carbonyl)glycinate (**5c**)

Following General Procedure C, **4a** (60 mg, 0.17 mmol) was reacted with glycine methyl ester hydrochloride (33 mg, 0.26 mmol). The crude was purified by flash column chromatography (pentane/EtOAc in gradient) to afford 45 mg (62%) of the title compound as a pale yellow solid. ^1^H NMR (CDCl_3_, 400 MHz, δ, ppm) 7.46 (1H, d, *J* = 7.8 Hz), 7.38–7.33 (1H, m), 7.23–7.15 (1H, m), 6.91 (1H, brd, *J* = 8.3 Hz), 6.75 (1H, brt, *J* = 7.6 Hz), 4.22 (2H, t, *J* = 5.1 Hz), 4.17 (2H, d, *J* = 5.6 Hz), 3.76 (6H, s), 3.18 (2H, t, *J* = 5.1 Hz), 2.33 (3H, s).

##### *tert*-Butyl-(2-(4-(2-(2-bromophenoxy)ethyl)-1,5-dimethyl-1*H*-pyrazole-3-carboxamido)ethyl)carbamate (**5d**)

Following general procedure C, **4a** (60 mg, 0.17 mmol) was reacted with *N*-boc-ethylenediamine (42 mg, 0.26 mmol). The crude was purified by flash column chromatography (pentane/EtOAc in gradient) to afford 65 mg (76%) of the title compound as a pale yellow solid. ^1^H NMR (CDCl_3_, 400 MHz, δ, ppm) 7.45 (1H, dd, *J* = 7.8, 1.6 Hz), 7.23–7.12 (2H, m), 6.92 (1H, dd, *J* = 8.3, 1.2 Hz), 6.75 (1H, td, *J* = 7.7, 1.4 Hz), 5.03 (1H, brs), 4.23 (2H, t, *J* = 5.9 Hz), 3.74 (3H, s), 3.48 (2H, t, *J* = 5.8 Hz), 3.37–3.26 (2H, m), 3.18 (2H, t, *J* = 5.9 Hz), 2.32 (3H, s), 1.40 (9H, s).

##### 4-(2-(2-Bromophenoxy)ethyl)-1,5-dimethyl-*N*-(2-oxopropyl)-1*H*-pyrazole-3-carboxamide (**5e**)

Following General Procedure C, **4a** (65 mg, 0.19 mmol) was reacted with aminoacetone hydrochloride (29 mg, 0.28 mmol). The crude was purified by flash column chromatography (pentane/EtOAc in gradient) to afford 54 mg (71%) of the title compound as a pale yellow solid. ^1^H NMR (CD_3_OD, 400 MHz, δ, ppm) 7.43 (1H, dd, *J* = 7.9, 1.6 Hz), 7.20 (1H, ddd, *J* = 8.3, 7.5, 1.6 Hz), 6.95 (1H, dd, *J* = 8.3, 1.3 Hz), 6.76 (1H, td, *J* = 7.7, 1.4 Hz), 4.18–4.12 (4H, m), 3.76 (3H, s), 3.12 (2H, t, *J* = 6.4 Hz), 2.27 (3H, s), 2.17 (3H, s).

##### 4-(2-(2-Bromophenoxy)ethyl)-*N*-(2-hydroxyethyl)-1,5-dimethyl-1*H*-pyrazole-3-carboxamide (**5f**)

Following General Procedure C, **4a** (65 mg, 0.19 mmol) was reacted with ethanol amine (18 mg, 0.28 mmol). The crude was purified by flash column chromatography (pentane/EtOAc in gradient) to afford 40 mg (54%) of the title compound as a pale yellow solid. ^1^H NMR (CD_3_OD, 400 MHz, δ, ppm) 7.44 (1H, dd, *J* = 7.9, 1.6 Hz), 7.22 (1H, ddd, *J* = 8.3, 7.4, 1.6 Hz), 6.97 (1H, dd, *J* = 8.3, 1.3 Hz), 6.77 (1H, td, *J* = 7.8, 1.4 Hz), 4.16 (2H, t, *J* = 6.4 Hz), 3.77 (3H, s), 3.65 (2H, t, *J* = 5.7 Hz), 3.43 (2H, t, *J* = 5.7 Hz), 3.14 (2H, t, *J* = 6.4 Hz), 2.29 (3H, s).

##### 4-(2-((2-Bromopyridin-3-yl)oxy)ethyl)-1,5-dimethyl-N-(pyridin-3-yl)-1*H*-pyrazole-3-carboxamide (**6a**)

Following General Procedure C, **4b** (50 mg, 0.14 mmol) was reacted with 3-aminopyridine (28 mg, 0.30 mmol). The crude was purified by flash column chromatography (pentane/EtOAc in gradient) to afford 40 mg (65%) of the title compound as a pale green solid. ^1^H NMR (400 MHz, CDCl_3_, δ, ppm) 8.82 (1H, s), 8.73 (1H, d, *J* = 2.4 Hz), 8.33 (1H, dd, *J* = 4.7, 1.4 Hz), 8.08 (1H, d, *J* = 2.7 Hz), 7.91 (1H, dd, *J* = 4.5, 1.5 Hz), 7.30–7.26 (1H, m), 7.22 (1H, dd, *J* = 8.1, 1.5 Hz), 7.16 (1H, dd, *J* = 8.1, 4.6 Hz), 4.28 (2H, t, *J* = 5.9 Hz), 3.82 (3H, s), 3.24 (2H, t, *J* = 5.9 Hz), 2.38 (3H, s).

##### (4-(2-((2-Bromopyridin-3-yl)oxy)ethyl)-1,5-dimethyl-1*H*-pyrazol-3-yl)(morpholino)-methanone (**6b**)

Following General Procedure C, **4b** (58 mg, 0.17 mmol) was reacted with morpholine (30 mg, 0.34 mmol). The crude was purified by flash column chromatography (pentane/EtOAc in gradient) to afford 45 mg (64%) of the title compound as a pale green solid. ^1^H NMR (CD_3_OD, 400 MHz, δ, ppm) 7.92 (1H, dd, *J* = 4.6, 1.3 Hz), 7.46 (1H, dd, *J* = 8.2, 1.3 Hz), 7.35 (1H, dd, *J* = 8.2, 4.7 Hz), 4.26 (2H, t, *J* = 6.5 Hz), 3.82 (3H, s), 3.76–3.72 (4H, m), 3.65 (3H, brs), 3.27–3.21 (2H, m), 3.06 (2H, t, *J* = 6.5 Hz), 2.36 (3H, s).

##### Methyl (4-(2-((2-Bromopyridin-3-yl)oxy)ethyl)-1,5-dimethyl-1*H*-pyrazole-3-carbonyl)-glycinate (**6c**)

Following General Procedure C, **4b** (65 mg, 0.19 mmol) was reacted with glycine methyl ester hydrochloride (36 mg, 0.29 mmol). The crude was purified by flash column chromatography (pentane/EtOAc in gradient) to afford 50 mg (63%) of the title compound as a pale green solid. ^1^H NMR (CDCl_3_, 400 MHz, δ, ppm) 7.87 (1H, dd, *J* = 4.5, 1.6 Hz), 7.34 (1H, t, *J* = 5.5 Hz), 7.20–7.16 (1H, m), 7.12 (1H, dd, *J* = 8.1, 4.6 Hz), 4.21 (2H, t, *J* = 6.0 Hz), 4.14 (2H, d, *J* = 5.7 Hz), 3.73 (3H, s), 3.72 (3H, s), 3.15 (2H, t, *J* = 6.0 Hz), 2.31 (3H, s).

##### *N-*(2-Amino-2-oxoethyl)-4-(2-((2-bromopyridin-3-yl)oxy)ethyl)-1,5-dimethyl-1*H*-pyrazole-3-carboxamide (**6d**)

Following General Procedure C, **4b** (63 mg, 0.18 mmol) was reacted with glycinamide hydrochloride (40 mg, 0.37 mmol). The crude was purified by flash column chromatography (pentane/EtOAc in gradient) to afford 51 mg (70%) of the title compound as a pale green solid. ^1^H NMR (400 MHz, DMSO-*d*_6_, δ, ppm) 7.93–7.91 (1H, m), 7.90 (1H, m), 7.56 (1H, dd, *J* = 8.1, 1.1 Hz), 7.37–7.34 (1H, m), 4.18 (2H, t, *J* = 6.7 Hz), 3.79–3.77 (5H, m), 3.08 (2H, t, *J* = 6.7 Hz), 2.27 (3H, s).

##### *tert*-Butyl 4-((4-(2-((2-Bromopyridin-3-yl)oxy)ethyl)-1,5-dimethyl-1*H*-pyrazole-3-carbox-amido)methyl)piperidine-1-carboxylate (**6e**)

Following General Procedure C, **4b** (56 mg, 0.17 mmol) was reacted with 1-boc-4-(aminomethyl)piperidine (53 mg, 0.24 mmol). The crude was purified by flash column chromatography (pentane/EtOAc in gradient) to afford 50 mg (56%) of the title compound as a pale green solid. ^1^H NMR (CD_3_OD, 400 MHz, δ, ppm) 7.86 (1H, dd, *J* = 4.7, 1.4 Hz), 7.43 (1H, dd, *J* = 8.2, 1.3 Hz), 7.30 (1H, dd, *J* = 8.2, 4.7 Hz), 4.24 (2H, t, *J* = 6.4 Hz), 4.07–4.03 (2H, m), 3.79 (3H, s), 3.23–3.20 (2H, m), 3.17 (2H, t, *J* = 6.4 Hz), 2.71 (2H, brs), 1.80–1.69 (3H, m), 2.31 (3H, s), 1.43 (9H, s), 1.12 (2H, qd, *J* = 12.5, 4.1 Hz).

##### 4-(2-((1-Bromonaphthalen-2-yl)oxy)ethyl)-1,5-dimethyl-*N*-(pyridin-3-yl)-1*H*-pyrazole-3-carboxamide (**7a**)

Following General Procedure C, **4c** (50 mg, 0.13 mmol) was reacted with 3-aminopyridine (24 mg, 0.25 mmol). The crude was purified by flash column chromatography (pentane/EtOAc in gradient) to afford 37 mg (62%) of the title compound as a pale brown solid. ^1^H NMR (400 MHz, CDCl_3_, δ, ppm) 8.82 (1H, brs), 8.71 (1H, brs), 8.34–8.29 (1H, m), 8.15 (1H, d, *J* = 8.6 Hz), 8.06 (brs, 1H), 7.73 (2H, t, *J* = 7.4 Hz), 7.54–7.47 (1H, m), 7.36–7.23 (3H, m), 4.42 (2H, t, *J* = 5.9 Hz), 3.79 (3H, s), 3.27 (2H, t, *J* = 5.8 Hz), 2.36 (3H, s).

##### (4-(2-((1-Bromonaphthalen-2-yl)oxy)ethyl)-1,5-dimethyl-1*H*-pyrazol-3-yl)(morpholino)-methanone (**7b**)

Following General Procedure C, **4c** (62 mg, 0.16 mmol) was reacted with morpholine (27 mg, 0.31 mmol). The crude was purified by flash column chromatography (pentane/EtOAc in gradient) to afford 45 mg (61%) of the title compound as a pale green solid. ^1^H NMR (CD_3_OD, 400 MHz, δ, ppm) 8.11 (1H, d, *J* = 8.6 Hz), 7.80 (2H, t, *J* = 8.8 Hz), 7.55–7.49 (1H, m), 7.40–7.34 (1H, m), 7.32 (1H, d, *J* = 9.0 Hz), 4.28 (2H, t, *J* = 6.5 Hz), 3.75 (3H, s), 3.67 (4H, brs), 3.51 (2H, brs), 3.20–3.12 (2H, m), 3.02 (2H, t, *J* = 6.5 Hz), 2.27 (3H, s).

##### Methyl (4-(2-((1-Bromonaphthalen-2-yl)oxy)ethyl)-1,5-dimethyl-1*H*-pyrazole-3-carbonyl)-glycinate (**7c**)

Following general procedure C, **4c** (60 mg, 0.15 mmol) was reacted with glycine methyl ester hydrochloride (38 mg, 0.31 mmol). The crude was purified by flash column chromatography (pentane/EtOAc in gradient) to afford 50 mg (70%) of the title compound as a pale brown solid. ^1^H NMR (CDCl_3_, 400 MHz, δ, ppm) 8.15 (1H, d, *J* = 8.5 Hz), 7.73 (1H, d, *J* = 5.0 Hz), 7.71 (1H, d, *J* = 3.7 Hz), 7.54–7.47 (1H, m), 7.38–7.31 (2H, m), 7.28 (1H, d, *J* = 9.0 Hz), 4.38 (2H, t, *J* = 6.0 Hz), 4.18 (2H, d, *J* = 5.7 Hz), 3.74 (3H, s), 3.73 (3H, s), 3.22 (2H, t, *J* = 6.0 Hz), 2.31 (3H, s).

##### *N*-(2-Amino-2-oxoethyl)-4-(2-((1-bromonaphthalen-2-yl)oxy)ethyl)-1,5-dimethyl-1*H*-pyrazole-3-carboxamide (**7d**)

Following General Procedure C, **4c** (65 mg, 0.16 mmol) was reacted with glycinamide hydrochloride (37 mg, 0.33 mmol). The crude was purified by flash column chromatography (pentane/EtOAc in gradient) to afford 51 mg (68%) of the title compound as a pale brown solid. ^1^H NMR (400 MHz, DMSO-*d*_6_, δ, ppm) 8.06 (1H, d, *J* = 8.2 Hz), 7.96 (1H, d, *J* = 8.9 Hz), 7.94–7.88 (2H, m), 7.61 (1H, ddd, *J* = 8.4, 6.8, 1.2 Hz), 7.56 (1H, d, *J* = 9.1 Hz), 7.46–7.41 (1H, m), 7.39 (1H, brs), 7.08 (1H, brs), 4.31 (2H, t, *J* = 7.6 Hz), 3.79 (2H, d, *J* = 5.7 Hz), 3.78 (3H, s), 3.12 (2H, t, *J* = 7.6 Hz), 2.28 (3H, s).

##### *tert-*Butyl 4-((4-(2-((1-Bromonaphthalen-2-yl)oxy)ethyl)-1,5-dimethyl-1*H*-pyrazole-3-carboxamido)methyl)piperidine-1-carboxylate (**7e**)

Following General Procedure C, **4c** (60 mg, 0.15 mmol) was reacted with 1-boc-4-(aminomethyl)piperidine (49 mg, 0.23 mmol). The crude was purified by flash column chromatography (pentane/EtOAc in gradient) to afford 59 mg (65%) of the title compound as a pale brown solid. ^1^H NMR (CD_3_OD, 400 MHz, δ, ppm) 8.14 (1H, d, *J* = 8.6 Hz), 7.73 (1H, d, *J* = 6.3 Hz), 7.71 (1H, d, *J* = 5.2 Hz), 7.54–7.46 (1H, m), 7.36–7.23 (2H, m), 7.00 (1H, t, *J* = 6.2 Hz), 4.39 (2H, t, *J* = 5.8 Hz), 4.09 (2H, q, *J* = 7.1 Hz), 3.73 (3H, s), 3.24–3.21 (4H, m), 2.68–2.62 (2H, m), 2.33 (3H, s), 1.72–1.69 (3H, m), 1.42 (9H, s), 1.20–1.10 (2H, m).

##### 1,5-Dimethyl-4-(2-(2-(2-(piperazin-1-yl)pyridin-4-yl)phenoxy)ethyl)-*N*-(pyridin-3-yl)-1*H*-pyrazole-3-carboxamide (**8a**)

Following General Procedure D, **5a** (80 mg, 0.19 mmol) was reacted with 2-(4-*tert*-butoxycarbonylpiperazin-1-yl)pyridine-4-boronic acid pinacol ester (93 mg, 0.24 mmol), the crude was purified by reversed-phase chromatography (6 g C18 column; ACN in water 0–100%). The resulting residue was reacted according to General Procedure E to afford the title compound as a fluffy off-white solid (40 mg, 70%). ^1^H NMR (CD_3_OD, 400 MHz, δ, ppm) 8.91 (1H, brs), 8.28 (1H, brs), 8.21–8.17 (1H, m), 8.13 (1H, dd, *J* = 5.3, 0.6 Hz), 7.43 (1H, dd, *J* = 8.2, 4.7 Hz), 7.35 (1H, ddd, *J* = 8.3, 7.4, 1.7 Hz), 7.29 (1H, dd, *J* = 7.6, 1.7 Hz), 7.14–7.10 (1H, m), 7.02 (1H, dd, *J* = 7.5, 1.0 Hz), 6.99 (1H, brs), 6.82 (1H, dd, *J* = 5.2, 1.3 Hz), 4.23 (2H, t, *J* = 6.7 Hz), 3.83 (3H, s), 3.81–3.76 (4H, m), 3.33–3.29 (4H, m), 3.14 (2H, t, *J* = 6.6 Hz), 2.04 (3H, s). ^13^C NMR (CD_3_OD, 100 MHz, δ, ppm) 162.08, 158.49, 155.73, 149.25, 146.70, 146.69, 143.50, 140.90, 140.86, 139.77, 129.89, 129.72, 128.40, 127.94, 120.61, 116.81, 116.04, 116.02, 112.63, 108.73, 68.09, 43.00, 42.66, 35.88, 23.50, 7.81. HRMS (ESI), found 498.2617 (C_28_H_31_N_7_O_2_), [M + H]^+^, requires 498.2617.

##### (1,5-Dimethyl-4-(2-(2-(2-(piperazin-1-yl)pyridin-4-yl)phenoxy)ethyl)-1*H*-pyrazol-3-yl)(morpholino)methanone (**8b**)

Following General Procedure D, **5b** (10 mg, 0.02 mmol) was reacted with 2-(4-*tert*-butoxycarbonylpiperazin-1-yl)pyridine-4-boronic acid pinacol ester (19 mg, 0.05 mmol), the crude was purified by reversed-phase chromatography (6 g C18 column; ACN in water 0–100%). The resulting residue was reacted according to General Procedure E to afford the title compound as a fluffy colorless solid (8 mg, 76%). ^1^H NMR (CD_3_OD, 400 MHz, δ, ppm) 8.13 (1H, dd, *J* = 5.3, 0.7 Hz), 7.36 (1H, ddd, *J* = 8.3, 7.4, 1.8 Hz), 7.31 (1H, dd, *J* = 7.6, 1.7 Hz), 7.10–7.07 (1H, m), 7.03 (1H, td, *J* = 7.5, 1.0 Hz), 6.97–6.96 (1H, m), 6.81 (1H, dd, *J* = 5.2, 1.3 Hz), 4.14 (2H, t, *J* = 6.6 Hz), 3.80–3.75 (4H, m), 3.74 (3H, s), 3.66 (4H, brs), 3.58 (2H, brs), 3.51 (2H, brs), 3.32–3.30 (4H, m), 2.91 (2H, t, *J* = 6.6 Hz), 2.03 (3H, s). ^13^C NMR (CD_3_OD, 100 MHz, δ, ppm) 166.09, 159.94, 157.15, 150.56, 148.02, 143.67, 140.23, 131.40, 131.19, 129.98, 122.30, 117.40, 116.84, 114.43, 110.07, 69.75, 68.02, 67.71, 44.81, 44.44, 44.24, 44.01, 36.85, 24.70, 9.30. HRMS (ESI), found 491.2772 (C_27_H_34_N_6_O_3_), [M + H]^+^, requires 491.2771.

##### (1,5-Dimethyl-4-(2-(2-(2-(piperazin-1-yl)pyridin-4-yl)phenoxy)ethyl)-1*H*-pyrazole-3-carbonyl)glycine (**8c**)

Following General Procedure D, **5c** (56 mg, 0.13 mmol) was reacted with 2-(4-*tert*-butoxycarbonylpiperazin-1-yl)pyridine-4-boronic acid pinacol ester (67 mg, 0.17 mmol), the crude was purified by reversed-phase chromatography (6 g C18 column; ACN in water 0–100%). The resulting residue was reacted according to General Procedure E to afford the title compound as a fluffy colorless solid (11 mg, 32%). ^1^H NMR (CD_3_OD, 400 MHz, δ, ppm) 8.09 (1H, dd, *J* = 5.4, 0.5 Hz), 7.43–7.38 (1H, m), 7.34 (1H, dd, *J* = 7.7, 1.7 Hz), 7.17–7.14 (1H, m), 7.05 (2H, td, *J* = 7.5, 1.0 Hz), 6.92 (1H, dd, *J* = 5.4, 0.5 Hz), 4.20 (2H, t, *J* = 6.5 Hz), 3.86–3.82 (4H, m), 3.80 (3H, s), 3.78 (2H, s), 3.42–3.38 (4H, m), 3.10 (2H, t, *J* = 6.5 Hz), 2.04 (3H, s). ^13^C NMR (CD_3_OD, 100 MHz, δ, ppm) 163.56, 157.42, 157.26, 153.50, 136.60, 133.86, 133.83, 133.45, 131.72, 130.07, 129.95, 126.90, 122.55, 117.33, 114.31, 113.71, 69.18, 52.40, 44.72, 43.71, 37.31, 24.59, 9.52. HRMS (ESI), found 479.2418 (C_25_H_30_N_6_O_4_), [M + H]^+^, requires 479.2407.

##### *N*-(2-Aminoethyl)-1,5-dimethyl-4-(2-(2-(2-(piperazin-1-yl)pyridin-4-yl)phenoxy)ethyl)-1*H*-pyrazole-3-carboxamide (**8f**)

Following General Procedure D, **5d** (50 mg, 0.10 mmol) was reacted with 2-(4-*tert*-butoxycarbonylpiperazin-1-yl)pyridine-4-boronic acid pinacol ester (51 mg, 0.13 mmol), the crude was purified by reversed-phase chromatography (6 g C18 column; ACN in water 0–100%). The resulting residue was reacted according to General Procedure E to afford the title compound as a fluffy off-white solid (22 mg, 70%). ^1^H NMR (CD_3_OD, 400 MHz, δ, ppm) 8.04 (1H, d, *J* = 6.6 Hz), 7.56 (1H, brs), 7.55–7.51 (1H, m), 7.48 (1H, dd, *J* = 7.4, 1.5 Hz), 7.31 (1H, dd, *J* = 6.6, 1.3 Hz), 7.23 (1H, d, *J* = 8.1 Hz), 7.14–7.09 (1H, m), 4.28–4.23 (2H, m), 4.10–4.05 (4H, m), 3.82 (3H s), 3.61 (2H, t, *J* = 5.9 Hz), 3.54–3.48 (4H, m), 3.16–3.12 (4H, m), 2.16 (3H, s). ^13^C NMR (CD_3_OD, 100 MHz, δ, ppm) 166.13, 157.53, 157.50, 153.57, 142.46, 140.77, 136.57, 133.55, 131.72, 126.65, 122.44, 117.43, 116.99, 114.29, 113.68, 69.58, 44.73, 43.74, 41.00, 37.79, 37.30, 24.72, 9.34. HRMS (ESI), found 464.2774 (C_25_H_33_N_7_O_2_), [M + H]^+^, requires 464.2774.

##### 1,5-Dimethyl-*N*-(2-oxopropyl)-4-(2-(2-(2-(piperazin-1-yl)pyridin-4-yl)phenoxy)ethyl)-1*H*-pyrazole-3-carboxamide (**8g**)

Following General Procedure D, **5e** (50 mg, 0.12 mmol) was reacted with 2-(4-*tert*-butoxycarbonylpiperazin-1-yl)pyridine-4-boronic acid pinacol ester (64 mg, 0.16 mmol), the crude was purified by reversed-phase chromatography (6 g C18 column; ACN in water 0–100%). The resulting residue was reacted according to General Procedure E to afford the title compound as a fluffy off-white solid (12 mg, 66%). ^1^H NMR (CD_3_OD, 400 MHz, δ, ppm) 8.12 (1H, d, *J* = 5.3 Hz), 7.36–7.31 (1H, m), 7.29 (1H, dd, *J* = 7.6, 1.5 Hz), 7.09 (1H, d, *J* = 8.1 Hz), 7.00 (1H, t, *J* = 7.4 Hz), 6.96 (1H, brs), 6.84–6.81 (1H, m), 4.23–4.12 (4H, m), 3.82–3.77 (4H, m), 3.75 (3H, s), 3.38–3.34 (4H, m), 3.06 (2H, t, *J* = 6.5 Hz), 2.18 (3H, s), 1.95 (3H, s). ^13^C NMR (CD_3_OD, 100 MHz, δ, ppm) 205.98, 165.29, 159.41, 157.08, 150.98, 147.39, 142.13, 140.82, 131.31, 131.25, 129.56, 122.00, 117.50, 117.29, 114.02, 110.33, 69.45, 49.95, 44.36, 44.02, 37.21, 27.09, 24.81, 9.21. HRMS (ESI), found 477.2615 (C_26_H_32_N_6_O_3_), [M + H]^+^, requires 477.2614.

##### *N*-(2-Hydroxyethyl)-1,5-dimethyl-4-(2-(2-(2-(piperazin-1-yl)pyridin-4-yl)phenoxy)ethyl)-1*H*-pyrazole-3-carboxamide (**8h**)

Following General Procedure D, **5f** (45 mg, 0.12 mmol) was reacted with 2-(4-*tert*-butoxycarbonylpiperazin-1-yl)pyridine-4-boronic acid pinacol ester (59 mg, 0.15 mmol), the crude was purified by reversed-phase chromatography (6 g C18 column; ACN in water 0–100%). The resulting residue was reacted according to General Procedure E to afford the title compound as a fluffy colorless solid (15 mg, 60%). ^1^H NMR (CD_3_OD, 400 MHz, δ, ppm) 7.37–7.32 (1H, m), 7.29 (1H, dd, *J* = 7.5, 1.6 Hz), 7.10 (1H, d, *J* = 8.2 Hz), 7.04–6.99 (1H, m), 6.97 (1H, s), 6.83 (1H, d, *J* = 5.2 Hz), 4.19 (2H, t, *J* = 6.7 Hz), 3.77–3–73 (7H, d, *J* = 12.1 Hz), 3.67 (2H, t, *J* = 5.6 Hz), 3.43 (2H, t, *J* = 5.5 Hz), 3.33–3.31 (s, 4H), 3.08 (2H, t, *J* = 6.6 Hz), 1.97 (3H, s). ^13^C NMR (CD_3_OD, 100 MHz, δ, ppm) 159.93, 157.15, 150.70, 148.15, 131.29, 131.15, 129.81, 122.00, 117.46, 114.31, 114.02, 111.43, 110.21, 69.57, 61.77, 44.48, 44.17, 42.38, 37.16, 24.97, 9.28. HRMS (ESI), found 465.2615 (C_25_H_32_N_6_O_3_), [M + H]^+^, requires 465.2614.

##### Methyl (1,5-Dimethyl-4-(2-(2-(2-(piperazin-1-yl)pyridin-4-yl)phenoxy)ethyl)-1*H*-pyrazole-3-carbonyl)glycinate (**8i**)

Following General Procedure D, **5c** (35 mg, 0.08 mmol) was reacted with 2-(4-*tert*-butoxycarbonylpiperazin-1-yl)pyridine-4-boronic acid pinacol ester (42 mg, 0.11 mmol), the crude was purified by reversed-phase chromatography (6 g C18 column; ACN in water 0–100%). The resulting residue was reacted according to General Procedure E to afford the title compound as a fluffy off-white solid (10 mg, 36%). ^1^H NMR (CD_3_OD, 400 MHz, δ, ppm) 8.03 (1H, d, *J* = 6.6 Hz), 7.56 (1H, brs), 7.53–7.47 (2H, m), 7.29 (1H, brd, *J* = 6.7 Hz), 7.22 (1H, d, *J* = 8.3 Hz), 7.12 (1H, t, *J* = 7.5 Hz), 4.25 (2H, t, *J* = 7.1 Hz), 4.07 (2H, s), 4.05–4.00 (4H, m), 3.81 (3H, s), 3.74 (3H, s), 3.53–3.49 (4H, m), 3.15 (2H, t, *J* = 7.1 Hz), 2.15 (3H, s). ^13^C NMR (CD_3_OD, 100 MHz, δ, ppm) 172.14, 165.55, 157.51, 157.42, 153.53, 142.36, 136.78, 133.62, 131.60, 126.64, 122.49, 117.37, 116.97, 114.56, 113.79, 69.53, 52.69, 44.72, 43.76, 41.50, 37.26, 24.66, 9.30. HRMS (ESI), found 493.2617 (C_26_H_32_N_6_O_4_), [M + H]^+^, requires 493.2617.

##### 1,5-Dimethyl-4-(2-((2′-(piperazin-1-yl)-[2,4′-bipyridin]-3-yl)oxy)ethyl)-*N*-(pyridin-3-yl)-1*H*-pyrazole-3-carboxamide (**9a**)

Following General Procedure D, **6a** (60 mg, 0.14 mmol) was reacted with 2-(4-*tert*-butoxycarbonylpiperazin-1-yl)pyridine-4-boronic acid pinacol ester (84 mg, 0.21 mmol), the crude was purified by reversed-phase chromatography (6 g C18 column; ACN in water 0–100%). The resulting residue was reacted according to General Procedure E to afford the title compound as a fluffy pale yellow solid (18 mg, 71%). ^1^H NMR (CD_3_OD, 400 MHz, δ, ppm) 9.57 (1H, d, *J* = 2.0 Hz), 8.87–8.82 (1H, m), 8.62 (1H, d, *J* = 5.5 Hz), 8.58 (1H, d, *J* = 5.1 Hz), 8.48 (1H, d, *J* = 8.7 Hz), 8.23 (1H, d, *J* = 6.4 Hz), 8.14–8.06 (2H, m), 8.02 (1H, brs), 7.50 (1H, d, *J* = 6.4 Hz), 4.56 (2H, t, *J* = 6.8 Hz), 4.25–4.17 (4H, m), 3.92 (3H, s), 3.59–3.55 (4H, m), 3.29 (2H, d, *J* = 6.7 Hz), 2.26 (3H, s). ^13^C NMR (CD_3_OD, 100 MHz, δ, ppm) 163.55, 156.82, 153.70, 147.13, 141.58, 141.55, 140.61, 139.20, 138.50, 137.15, 137.03, 136.80, 133.20, 130.18, 130.03, 128.87, 117.34, 115.60, 115.48, 71.12, 44.90, 43.65, 37.68, 24.32, 9.53. HRMS (ESI), found 499.2570 (C_27_H_30_N_8_O_2_), [M + H]^+^, requires 499.2570.

##### (1,5-Dimethyl-4-(2-((2′-(piperazin-1-yl)-[2,4′-bipyridin]-3-yl)oxy)ethyl)-1*H*-pyrazol-3-yl)(morpholino)methanone (**9b**)

Following General Procedure D, **6b** (60 mg, 0.14 mmol) was reacted with 2-(4-*tert*-butoxycarbonylpiperazin-1-yl)pyridine-4-boronic acid pinacol ester (74 mg, 0.19 mmol), the crude was purified by reversed-phase chromatography (6 g C18 column; ACN in water 0–100%). The resulting residue was reacted according to General Procedure E to afford the title compound as a fluffy yellow solid (15 mg, 52%). ^1^H NMR (CD_3_OD, 400 MHz, δ, ppm) 8.64 (1H, d, *J* = 5.3 Hz), 8.58 (1H, d, *J* = 8.8 Hz), 8.26 (1H, d, *J* = 6.4 Hz), 8.21 (1H, dd, *J* = 8.6, 5.6 Hz), 8.06 (1H, brs), 7.48 (1H, d, *J* = 6.4 Hz), 4.53 (2H, t, *J* = 6.2 Hz), 4.23–4.23 (4H, m), 3.89 (3H, s), 3.68 (8H, brs), 3.61–3.53 (4H, m), 3.07 (2H, t, *J* = 6.1 Hz), 2.31 (3H, s). ^13^C NMR (CD_3_OD, 100 MHz, δ, ppm) 163.56, 156.84, 153.35, 145.38, 142.46, 142.28, 138.42, 138.14, 135.54, 132.00, 130.67, 116.73, 115.87, 115.37, 71.40, 67.85, 45.06, 44.77, 43.58, 37.04, 24.07, 9.77. HRMS (ESI), found 492.2723 (C_26_H_33_N_7_O_3_), [M + H]^+^, requires 492.2723.

##### (1,5-Dimethyl-4-(2-((2′-(piperazin-1-yl)-[2,4′-bipyridin]-3-yl)oxy)ethyl)-1*H*-pyrazole-3-carbonyl)glycine (**9c**)

Following General Procedure D, **6c** (50 mg, 0.12 mmol) was reacted with 2-(4-*tert*-butoxycarbonylpiperazin-1-yl)pyridine-4-boronic acid pinacol ester (62 mg, 0.15 mmol), the crude was purified by reversed-phase chromatography (6 g C18 column; ACN in water 0–100%). The resulting residue was reacted according to General Procedure E to afford the title compound as a fluffy pale yellow solid (14 mg, 53%). ^1^H NMR (CD_3_OD, 400 MHz, δ, ppm) 8.56 (1H, d, *J* = 5.1 Hz), 8.46 (1H, d, *J* = 8.8 Hz), 8.22 (1H, d, *J* = 6.3 Hz), 8.07 (1H, dd, *J* = 8.7, 5.3 Hz), 7.94 (1H, brs), 7.43 (1H, d, *J* = 6.3 Hz), 4.52 (2H, t, *J* = 6.5 Hz), 4.17 (4H, brs), 4.05 (2H, s), 3.83 (3H, s), 3.56 (4H, brs), 3.22 (2H, t, *J* = 6.4 Hz), 2.21 (3H, s). ^13^C NMR (CD_3_OD, 100 MHz, δ, ppm) 173.08, 172.12, 165.39, 156.90, 153.68, 146.81, 142.33, 140.80, 139.09, 138.53, 136.63, 130.56, 130.22, 116.12, 115.75, 115.46, 71.24, 44.89, 43.67, 41.46, 37.38, 24.10. HRMS (ESI), found 480.2359 (C_24_H_29_N_7_O_4_), [M + H]^+^, requires 480.2359.

##### *N*-(2-Amino-2-oxoethyl)-1,5-dimethyl-4-(2-((2′-(piperazin-1-yl)-[2,4′-bipyridin]-3-yl)oxy)ethyl)-1*H*-pyrazole-3-carboxamide (**9d**)

Following General Procedure D, **6d** (50 mg, 0.12 mmol) was reacted with 2-(4-*tert*-butoxycarbonylpiperazin-1-yl)pyridine-4-boronic acid pinacol ester (64 mg, 0.16 mmol), the crude was purified by reversed-phase chromatography (6 g C18 column; ACN in water 0–100%). The resulting residue was reacted according to General Procedure E to afford the title compound as a fluffy pale yellow solid (15 mg, 56%). ^1^H NMR (CD_3_OD, 400 MHz, δ, ppm) 8.62 (2H, brs), 8.24–8.18 (2H, m), 8.03 (1H, brs), 7.42 (1H, d, *J* = 5.8 Hz), 4.54 (2H, brs), 4.25 (4H, brs), 4.02 (2H, d, *J* = 6.3 Hz), 3.85 (3H, s), 3.59 (4H, brs), 3.23 (2H, brs), 2.24 (3H, s). ^13^C NMR (CD_3_OD, 100 MHz, δ, ppm) 175.20, 164.86, 157.01, 153.24, 145.30, 142.09, 141.30, 138.13, 135.45, 132.15, 130.72, 116.63, 116.22, 115.30, 71.62, 45.06, 43.62, 42.52, 37.44, 24.04, 9.61. HRMS (ESI), found 479.2553 (C_24_H_30_N_8_O_3_), [M + H]^+^, requires 479.2519.

##### *N*-(2-Aminoethyl)-1,5-dimethyl-4-(2-((2′-(piperazin-1-yl)-[2,4′-bipyridin]-3-yl)oxy)ethyl)-1*H*-pyrazole-3-carboxamide (**9e**)

Following General Procedure D, **6e** (50 mg, 0.09 mmol) was reacted with 2-(4-*tert*-butoxycarbonylpiperazin-1-yl)pyridine-4-boronic acid pinacol ester (52 mg, 0.13 mmol), the crude was purified by reversed-phase chromatography (6 g C18 column; ACN in water 0–100%). The resulting residue was reacted according to General Procedure E to afford the title compound as a fluffy pale yellow solid (12 mg, 61%). ^1^H NMR (CD_3_OD, 400 MHz, δ, ppm) 8.64 (1H, s), 8.63 (1H, s), 8.26 (1H, d, *J* = 6.4 Hz), 8.21 (1H, dd, *J* = 8.5, 5.7 Hz), 8.06 (1H, brs), 7.47 (1H, d, *J* = 6.4 Hz), 4.53 (2H, t, *J* = 6.6 Hz), 4.25 (4H, brs), 3.84 (3H, s), 3.59 (4H, brs), 3.42 (2H, d, *J* = 12.5 Hz), 3.28 (2H, d, *J* = 6.2 Hz), 3.22 (2H, t, *J* = 6.5 Hz), 3.00 (2H, t, *J* = 12.0 Hz), 2.23 (3H, s), 1.98–1.90 (3H, m), 1.58–1.47 (m, 2H). ^13^C NMR (CD_3_OD, 100 MHz, δ, ppm) 164.95, 157.04, 153.38, 145.57, 142.55, 141.11, 138.23, 138.19, 135.59, 131.83, 130.72, 116.56, 115.75, 115.34, 71.68, 45.04, 44.89, 44.41, 43.62, 37.27, 35.52, 27.67, 24.15, 9.48. HRMS (ESI), found 519.3196 (C_28_H_38_N_8_O_2_), [M + H]^+^, requires 519.3196.

##### 1,5-Dimethyl-4-(2-((1-(2-(piperazin-1-yl)pyridin-4-yl)naphthalen-2-yl)oxy)ethyl)-*N*-(pyridin-3-yl)-1*H*-pyrazole-3-carboxamide (**10a**)

Following General Procedure D, **7a** (60 mg, 0.13 mmol) was reacted with 2-(4-*tert*-butoxycarbonylpiperazin-1-yl)pyridine-4-boronic acid pinacol ester (75 mg, 0.19 mmol), the crude was purified by reversed-phase chromatography (6 g C18 column; ACN in water 0–100%). The resulting residue was reacted according to General Procedure E to afford the title compound as a fluffy pale brown solid (18 mg, 64%). ^1^H NMR (CD_3_OD, 400 MHz, δ, ppm) 9.25 (2H, brs), 8.42 (1H, d, *J* = 8.2 Hz), 8.22 (1H, d, *J* = 5.1 Hz), 7.89 (1H, d, *J* = 9.0 Hz), 7.80 (1H, d, *J* = 4.8 Hz), 7.74–7.63 (1H, m), 7.45 (1H, d, *J* = 9.0 Hz), 7.32–7.29 (3H, m), 6.87 (1H, brs), 6.67 (1H, d, *J* = 5.0 Hz), 4.27 (2H, t, *J* = 6.0 Hz), 3.84 (4H, brs), 3.82 (3H, s), 3.35 (4H, brs), 3.08–3.01 (2H, m), 1.93 (3H, s). ^13^C NMR (CD_3_OD, 100 MHz, δ, ppm) 163.50, 159.28, 154.22, 150.15, 147.15, 141.78, 141.52, 141.42, 140.44, 138.62, 133.77, 132.27, 131.24, 130.42, 129.14, 127.77, 125.24, 124.83, 124.17, 118.97, 118.61, 116.05, 112.25, 70.80, 44.34, 43.92, 37.45, 25.24, 9.16. HRMS (ESI), found 548.2775 (C_32_H_33_N_7_O_2_), [M + H]^+^, requires 548.2774.

##### (1,5-Dimethyl-4-(2-((1-(2-(piperazin-1-yl)pyridin-4-yl)naphthalen-2-yl)oxy)ethyl)-1*H*-pyrazol-3-yl)(morpholino)methanone (**10b**)

Following General Procedure D, **7b** (60 mg, 0.13 mmol) was reacted with 2-(4-*tert*-butoxycarbonylpiperazin-1-yl)pyridine-4-boronic acid pinacol ester (66 mg, 0.17 mmol), the crude was purified by reversed-phase chromatography (6 g C18 column; ACN in water 0–100%). The resulting residue was reacted according to General Procedure E to afford the title compound as a fluffy off-white solid (20 mg, 55%). ^1^H NMR (CD_3_OD, 400 MHz, δ, ppm) 8.16 (1H, d, *J* = 6.4 Hz), 8.01 (1H, d, *J* = 9.1 Hz), 7.89 (1H, d, *J* = 7.9 Hz), 7.52–7.48 (2H, m), 7.48–7.38 (3H, m), 7.04 (1H, d, *J* = 6.2 Hz), 4.25 (2H, t, *J* = 6.7 Hz), 4.13–4.05 (4H, m), 3.79 (3H, s), 3.65 (6H, brs), 3.58 (2H, brs), 3.54–3.47 (4H, m), 2.88 (2H, t, *J* = 6.6 Hz), 2.15 (3H, s). ^13^C NMR (CD_3_OD, 100 MHz, δ, ppm) 165.80, 156.39, 154.21, 153.85, 143.93, 140.03, 137.58, 132.99, 132.73, 130.49, 129.46, 128.64, 125.37, 124.72, 122.09, 119.13, 116.38, 116.24, 116.09, 70.87, 68.09, 67.73, 44.77, 44.68, 43.77, 43.70, 37.03, 25.00, 9.46. HRMS (ESI), found 541.2927 (C_31_H_36_N_6_O_3_), [M + H]^+^, requires 541.2927.

##### (1,5-Dimethyl-4-(2-((1-(2-(piperazin-1-yl)pyridin-4-yl)naphthalen-2-yl)oxy)ethyl)-1*H*-pyrazole-3-carbonyl)glycine (**10c**)

Following General Procedure D, **7c** (45 mg, 0.10 mmol) was reacted with 2-(4-*tert*-butoxycarbonylpiperazin-1-yl)pyridine-4-boronic acid pinacol ester (50 mg, 0.12 mmol), the crude was purified by reversed-phase chromatography (6 g C18 column; ACN in water 0–100%). The resulting residue was reacted according to General Procedure E to afford the title compound as a fluffy pale brown solid (13 mg, 61%). ^1^H NMR (CD_3_OD, 400 MHz, δ, ppm) 8.17 (1H, d, *J* = 6.4 Hz), 7.98 (1H, d, *J* = 9.1 Hz), 7.86 (1H, d, *J* = 8.0 Hz), 7.54–7.47 (2H, m), 7.43 (1H, t, *J* = 7.3 Hz), 7.41–7.35 (2H, m), 7.01 (1H, d, *J* = 6.3 Hz), 4.27 (2H, dt, *J* = 12.6, 6.2 Hz), 4.11 (4H, brs), 4.08–4.06 (2H, m), 3.81 (3H, s), 3.54 (4H, brs), 3.04 (2H, dt, *J* = 13.3, 6.3 Hz), 2.08 (3H, s). ^13^C NMR (CD_3_OD, 100 MHz, δ, ppm) 172.17, 165.13, 156.78, 154.22, 153.33, 142.04, 140.98, 137.11, 132.84, 132.74, 130.39, 129.40, 128.60, 125.30, 124.73, 121.91, 119.09, 117.37, 116.27, 70.84, 44.71, 43.67, 41.56, 37.50, 24.99, 9.49. HRMS (ESI), found 529.2563 (C_29_H_32_N_6_O_4_), [M + H]^+^, requires 529.2563.

##### *N*-(2-Amino-2-oxoethyl)-1,5-dimethyl-4-(2-((1-(2-(piperazin-1-yl)pyridin-4-yl)naphthalen-2-yl)oxy)ethyl)-1*H*-pyrazole-3-carboxamide (**10d**)

Following General Procedure D, **7d** (48 mg, 0.11 mmol) was reacted with 2-(4-*tert*-butoxycarbonylpiperazin-1-yl)pyridine-4-boronic acid pinacol ester (54 mg, 0.14 mmol), the crude was purified by reversed-phase chromatography (6 g C18 column; ACN in water 0–100%). The resulting residue was reacted according to General Procedure E to afford the title compound as a fluffy pale brown solid (16 mg, 60%). ^1^H NMR (CD_3_OD, 400 MHz, δ, ppm) 8.24 (1H, d, *J* = 5.1 Hz), 7.90 (1H, d, *J* = 9.1 Hz), 7.85–7.80 (1H, m), 7.47 (1H, d, *J* = 9.1 Hz), 7.36–7.30 (3H, m), 6.75 (1H, brs), 6.69 (1H, d, *J* = 5.1 Hz), 4.34–4.27 (1H, m), 4.18–4,13 (1H, m), 4.05 (1H, d, *J* = 17 Hz), 3.92 (d, *J* = 17.0 Hz, 1H), 3.80 (4H, brt, *J* = 5.3 Hz), 3.76 (3H, s), 3.35 (4H, brt, *J* = 5.3 Hz), 3.06–2.93 (2H, m), 1.91 (3H, s). ^13^C NMR (CD_3_OD, 100 MHz, δ, ppm) 174.41, 159.52, 154.29, 149.83, 147.57, 133.84, 131.14, 130.62, 129.15, 127.69, 125.35, 124.97, 124.93, 118.88, 116.87, 111.79, 71.57, 44.47, 43.82, 42.53, 37.25, 25.29, 9.30. HRMS (ESI), found 528.2723 (C_29_H_33_N_7_O_3_), [M + H]^+^, requires 528.2723.

##### *N*-(2-Aminoethyl)-1,5-dimethyl-4-(2-((1-(2-(piperazin-1-yl)pyridin-4-yl)naphthalen-2-yl)oxy)ethyl)-1*H*-pyrazole-3-carboxamide (**10e**)

Following General Procedure D, **7e** (45 mg, 0.08 mmol) was reacted with 2-(4-*tert*-butoxycarbonylpiperazin-1-yl)pyridine-4-boronic acid pinacol ester (42 mg, 0.11 mmol), the crude was purified by reversed-phase chromatography (6 g C18 column; ACN in water 0–100%). The resulting residue was reacted according to General Procedure E to afford the title compound as a fluffy pale brown solid (18 mg, 78%). ^1^H NMR (CD_3_OD, 400 MHz, δ, ppm) 8.18 (1H, d, *J* = 6.2 Hz), 8.02 (1H, d, *J* = 9.1 Hz), 7.88 (1H, d, *J* = 8.0 Hz), 7.56–7.50 (2H, m), 7.47–7.40 (2H, m), 7.39 (1H, t, *J* = 7.6 Hz), 7.04 (1H, d, *J* = 6.1 Hz), 4.29 (2H, t, *J* = 6.4 Hz), 4.14 (4H, brs), 3.84 (3H, s), 3.54 (4H, brs), 3.39 (2H, d, *J* = 12.1 Hz), 3.28 (2H, d, *J* = 5.1 Hz), 3.07 (2H, t, *J* = 6.2 Hz), 2.97 (2H, t, *J* = 12.1 Hz), 2.13 (3H, s), 1.96–1.87 (3H, m), 1.55–1.46 (2H, m). ^13^C NMR (CD_3_OD, 100 MHz, δ, ppm) 164.86, 156.77, 154.23, 153.46, 142.44, 137.15, 132.89, 132.80, 130.35, 129.43, 128.66, 125.29, 124.68, 121.65, 119.14, 116.99, 116.45, 115.87, 70.64, 44.86, 44.75, 44.47, 43.68, 37.28, 35.50, 27.66, 25.02, 9.40. HRMS (ESI), found 568.3403 (C_33_H_41_N_7_O_2_), [M + H]^+^, requires 568.3400.

#### Docking of Compound **8g** into the Binding Pocket of *Pv*NMT

The hybrid compound **8g** was rendered in two-dimensional (2D) images using the ChemDraw Professional (Version 19.1.1.21) software package, converted to SDF format, and then prepared for docking using the Molecular Operating Environment (MOE 2019.01) software package. After loading the SDF files, it was processed as follows: the compound was energy-minimized and partial charges were added (Amber10 force field) using QuickPrep. To prepare the enzyme, the *Pv*NMT PDB file was loaded into MOE and processed using QuickPrep. The docking simulation was set up by setting the receptor to “receptor+solvent”. The SDF file containing the processed ligands to be docked was loaded. Ligand placement and refinement were performed using the α PMI and induced fit methods, with 30 and 3 poses, respectively.

#### Cloning, Expression, and Purification of NMT Enzymes

Cloning, expressing, and purifying were carried out in accordance with established protocols as outlined by the Seattle Structural Genomics Center for Infectious Disease (SSGCID),^46,47^ as detailed in prior publications.^32,36,48,49^ A segment of the *Pv*NMT gene, encompassing residues 27–410, or the *Hs*NMT Isoform 1 gene, encompassing residues 1–496, and including an *N*-terminal 6xHis sequence along with a PreScission cleavage site, was cloned into a pET11a expression vector. The *N*-terminal sequence is MGSSHHHHHHSAALEVLFQ/GP-ORF, where cleavage occurs between the glutamine and glycine residues. *E. coli* Rosetta 2 (DE3) cells containing pRARE were transformed with the plasmid and expression tested. For large-scale preparations, 4–12 L of culture were grown using autoinduction media^50^ in shaker flasks (225 rpm) for 18–22 h at 18 °C. The expression clones were assigned the SSGCID target identifiers PlviB.18219.a.FR2.GE40922 and HosaA.18219.b.FR1.GEGE43395 for *Pv*NMT and *Hs*NMT, respectively, and are available at https://www.ssgcid.org/available-materials/ssgcid-proteins/.

NMT was purified following a 5-step procedure as previously described.^30,32^ The purification was started by passing the crude extract over a Ni^2+^-affinity chromatography (IMAC) HisTrap FF 5 mL column. The eluted protein was then purified further via anion exchange chromatography using a HiTRAP Q HP 5 mL column. Peak fractions were then selected and combined for cleavage of the 6xHis-tag and passed through a second IMAC column to remove the protease and any uncleaved NMT. Finally, peak fractions were concentrated to 5 mL and injected into a Superdex 75 10/300 column. The final buffer was composed of 0.3 M NaCl, 20 mM HEPES, 5% (v/v) glycerol, and 1 mM TCEP, pH 7.0; notably, TCEP was omitted from solutions containing NMT prepared for inhibition assays. Purity was verified via sodium dodecyl sulfate polyacrylamide gel electrophoresis (SDS-PAGE) analysis and final samples were concentrated, flash-frozen, and stored at −80 °C.

#### Crystallization and Structure Determination of *P. vivax* NMT

Purified *Pv*NMT (27–410) concentrated to 6 mg/mL was incubated with 1 mM MyrCoA (MedChem101 LLC.) and compounds **9c** or **10b** for 20 min at room temperature and then combined with solutions from the JCSG+ HT96 (Molecular Dimensions) and Morpheus HT96 (Molecular Dimensions) screens in 96-well sitting drop plates. Crystals formed within 2 weeks in JCSG+ condition D6 composed of 0.2 M magnesium chloride, 0.1 M Tris, pH 8.5, and 20% PEG 8000. Crystals were transferred to cryoprotectant solution containing the crystallant and 25% PEG 300 and then flash-frozen in liquid nitrogen. Cryocooled crystals were irradiated at the Advanced Light Source (ALS), Berkeley National Laboratory, coordinated within the ALS-ENSEMBLE Collaborative Crystallography program. X-ray diffraction images were recorded at 100 K on ALS-ENABLE beamline 8.2.2 equipped with a Quantum 315 CCD detector. The X-ray diffraction images can be retrieved from the Integrated Resource for Reproducibility in Macromolecular Crystallography at www.proteindiffraction.org.^51^ Structure factor amplitudes and space groups were determined using X-ray Detector Software (XDS) and Aimless.^52−54^ Initial phases were calculated via molecular replacement using Phaser^55^ with search models that are high-resolution structures of *Pv*NMT bound to related inhibitors (including PDB entry 5V0W). The molecular replacement solutions were refined and augmented iteratively using Phenix^56^ and Coot^57^ and then validated with Molprobity.^58^ X-ray diffraction and structural refinement statistics are provided in Supplementary Table S1.

#### NMT Activity Assay

The synthetic peptide derived from the *N*-terminal sequence (amino acids 2–16) of *P. falciparum* ADP-ribosylation factor (*Pf*ARF), Gly-Leu-Tyr-Val-Ser-Arg-Leu-Phe-Asn-Arg-Leu-Phe-Gln-Lys-Lys-NH_2_ was purchased from Innopep (San Diego, California). 7-Diethylamino-3-(4′-maleimidylphenyl)-4-methylcoumarin (CPM) was purchased from Thermo Scientific Life Technologies (Grand Island, New York), and the cosubstrate MyrCoA was purchased from Med Chem 101 LLC (Plymouth Meeting, Pennsylvania). IC_50_ calculations were calculated using Prism (GraphPad Software, Inc.).

To measure the activity of the purified *Pv*NMT an assay was adapted from Goncalves et al.^40^ as described in Harupa et al.^32^ and Rodríguez-Hernández et al.^36^ The assay buffer was prepared in a 4× stock solution consisting of 9.2 mM potassium phosphate, 69.7 mM sodium phosphate, 2 mM ethylenediamine tetraacetic acid (EDTA) and 0.04% TritonX-100 at pH 7.0. Prior to each experiment, assay buffers supplemented with 1 or 5% DMSO were prepared, and the enzyme was diluted in the former solution at a final concentration of 25 nM for *Pv*NMT or 7.8 nM for *Hs*NMT. To determine IC_50_, test compounds were prepared in a 6-dilution series at the desired concentration range in 10% (v/v) DMSO. Ten μL of the test compound or 10% (v/v) DMSO/water were dispensed into a 96-well plate (Greiner Bio-One) and 50 μL of enzyme (in assay buffer containing 1% DMSO) were added. The plate was incubated at room temperature for 30 min before combining 50 μL of reaction substrates containing 10 μM MyrCoA and the synthetic peptide (*Pf*ARF), as well as 8 μM CPM. Fluorescence measurements were recorded using a Spectra M2 plate reader (Molecular Devices) with excitation at 385 nm and emission at 485 nm. Fluorescence intensity was measured continuously over 1 min intervals for 45 min. Background fluorescence and noise were estimated using assay buffer containing all components, substituting 1% DMSO in lieu of enzyme, and values were subtracted from the experimental estimates. In lieu of compound, 10 μL of 10% DMSO/H_2_O was added to each well to determine the maximum enzyme activity. While measuring the fluorescence for 45 min, a linear reaction rate was observed over the first 30 min, and values were noted at the 30 min time-point.

## Data Availability

PDB codes for *Pv*NMT with bound compounds **9c** and **10b** are 8VKA and 8VKB respectively.

## Supporting Information Available

Unique conformational state of Aa loop observed in chain C of *Pv*NMT-**9c** and **10b** crystal structures, NMR spectra and HPLC chromatograms of all compounds, X-ray data collection, and refinement statistics (PDF)
Molecular formula strings (CSV)

## Abbreviations Used

ACN: acetonitrile
AcOH: acetic acid
BOC: *tert*-butyloxycarbonyl
CMBP: cyanomethyiene tributylphosphorane
CoA: Coenzyme A
CPM: 7-diethylamino-3-(4-maleimidyphenyl)-4-methylcoumarin
DCE: dichloroethane
DCM: dichloromethane
DIPEA: *N,N*-diisopropylethylamine
DMSO: dimethyl sulfoxide
DMF: *N,N*-dimethylformamide
EDTA: ethylenediaminetetraacetic acid
EtOAc: ethyl acetate
EtOH: ethanol
Equiv: equivalents
G6PD: glucose-6-phosphate dehydrogenase
HATU: 1-[bis(dimethylamino)methylene]-1*H*-1,2,3-triazolo-[4,5-*b*]pyridinium 3-oxid hexafluorophosphate
HCl: hydrogen chloride
HRMS: high-resolution mass spectrometry
*Hs*NMT: *Homo sapiens**N*-myristoyltransferase
IMAC: immobilized metal ion affinity chromatography
K_3_PO_4_: potassium phosphate
KOH: potassium hydroxide
MeOH: methanol
MyrCoA: myristoyl-coenzyme A
NMT: *N*-myristoyltransferase
N_2_: nitrogen
NaBH_4_: sodium borohydride
NBS: *N*-bromosuccinimide
Pd(PPh_3_)_4_: tetrakis(triphenylphosphine) palladium(0)
PDB: protein data bank
*Pf*NMT: *Plasmodium falciparum**N*-myristoyltransferase
*Pf*ARF: *Plasmodium falciparum* ADP-ribosylation factor
*Pv*NMT: *Plasmodium vivax**N*-myristoyltransferase
RMSD: root-mean-square deviation
SI: selectivity index
TFA: trifluoroacetic acid
