# Supplementary material for: Exploring Subsite Selectivity within Plasmodium vivaxN-Myristoyltransferase Using Pyrazole-Derived Inhibitors
Source: J Med Chem. 2024 Apr 29;67(9):7312–29. doi: 10.1021/acs.jmedchem.4c00168 (PMC11089503; doi:10.1021/acs.jmedchem.4c00168)
Supplement: Supplementary file 1 — jm4c00168_si_001.pdf [file jm4c00168_si_001.pdf]

Supporting information

Exploring subsite selectivity within *Plasmodium vivax N*-myristoyltransferase using pyrazole-derived inhibitors

Diego Rodríguez-Hernández[†,‡,#], Michael K. Fenwick[||,§,#], Rachael Zigweid[||,§], Banumathi Sankaran[⊥], Peter J. Myler[||,§,¶], Per Sunnerhagen[†], Alexis Kaushansky[§,¶], Bart L. Staker[||,§,*], Morten Grøtli[†,*]

[†]Department of Chemistry and Molecular Biology, University of Gothenburg; S-405 30 Gothenburg, Sweden.

[‡]Department of Structural and Functional Biology, Synthetic Biology Laboratory, Institute of Biology, University of Campinas, Campinas, SP, 13083-862, Brazil.

[||]Seattle Structural Genomics Center for Infectious Disease, Seattle, Washington 98109, United States.

[§]Center for Global Infectious Disease Research, Seattle Children's Research Institute, Seattle, Washington 98109, United States.

[⊥]Molecular Biophysics and Integrated Bioimaging, Berkeley Center for Structural Biology, Advanced Light Source, Berkeley National Laboratory, Berkeley, California 94720, United States.

[¶]Department of Pediatrics, University of Washington, Seattle, Washington 98195, United States.

[#] These authors contributed equally to this work

*Corresponding author. Email: grotli@chem.gu.se, Bart.Staker@seattlechildrens.org

Table of contents:

Supplementary Figures 1-36……………………………………………………….………S2-S22

Supplementary Table 1..…………………………………………………………………...S23

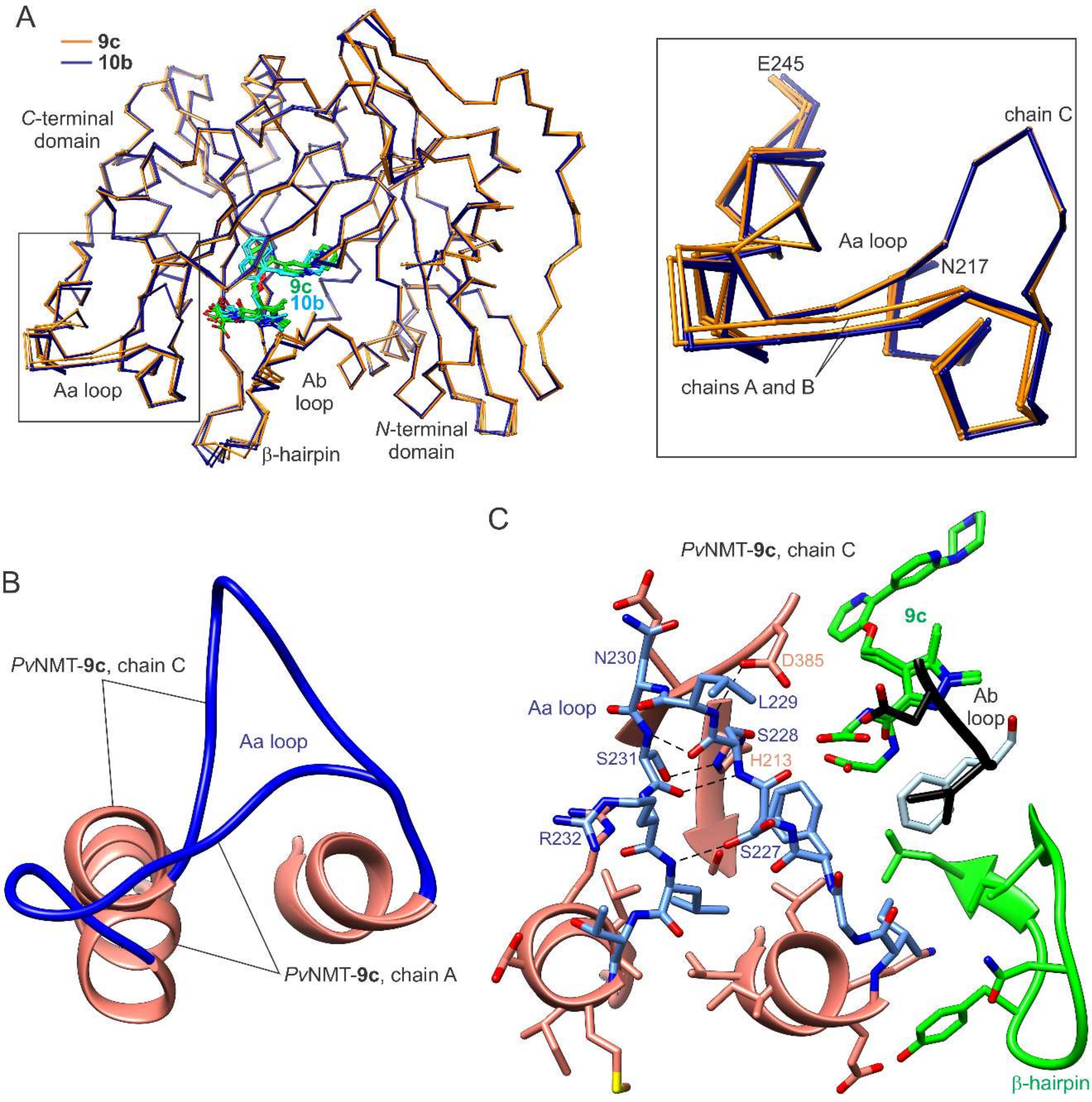

Figure S1. Unique conformational state of Aa loop observed in chain C of *Pv*NMT-**9c** and **10b** crystal structures. (A) Superimposed *Cα* traces. Inset provides close-up view of Aa loops. (B) Helix flanking Aa loop on *C*-terminal side exhibits conformational change. (C) Potential hydrogen bonds, indicated with dashed lines.

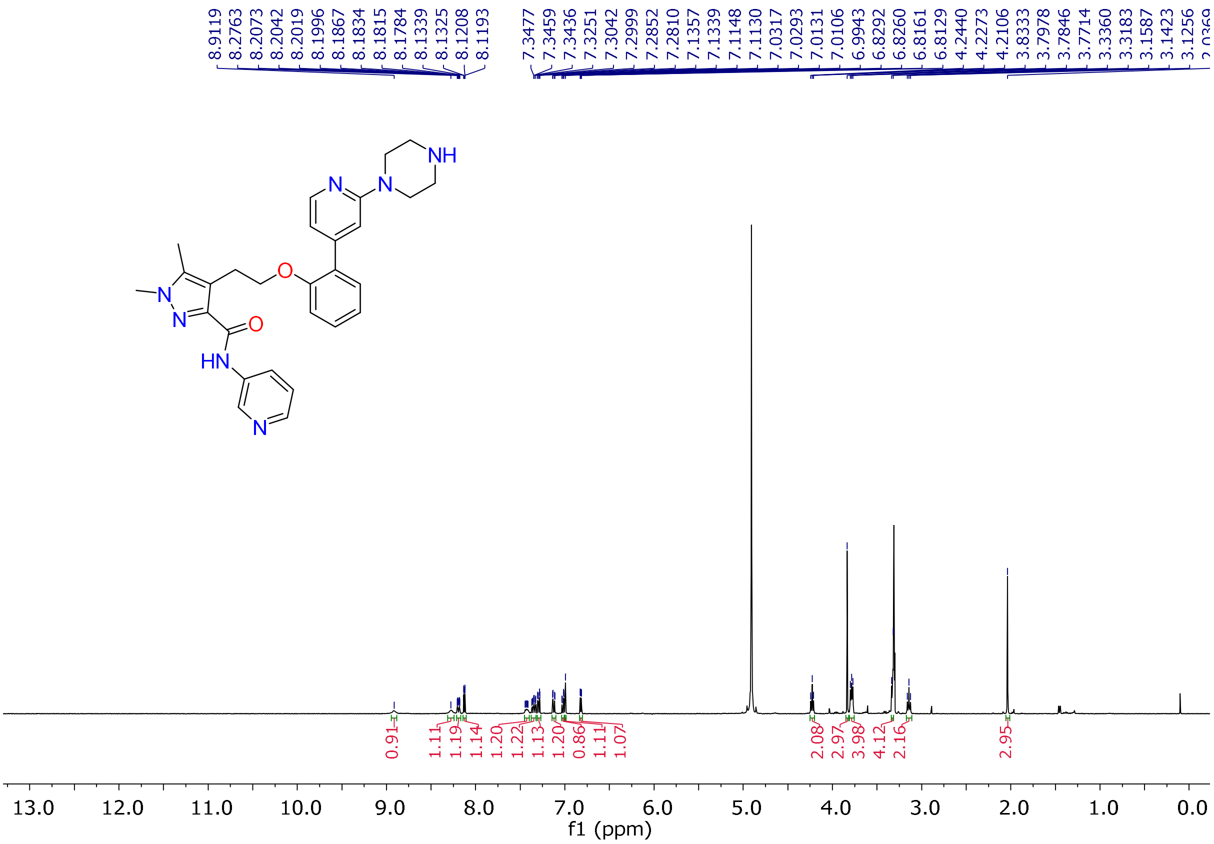

Figure S2. ^{1}H-NMR spectrum of compound **8a** (CD_3OD, 400 MHz).

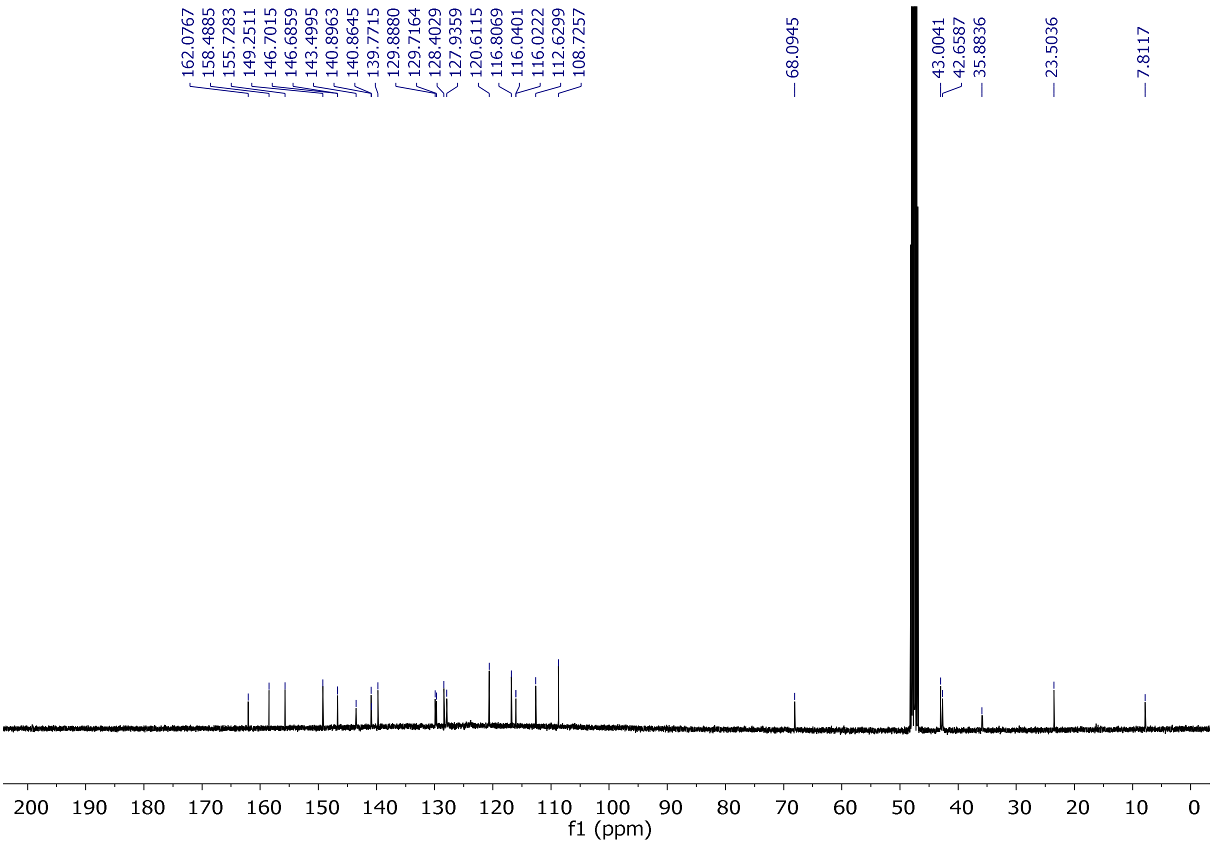

Figure S3. ^{13}C-NMR spectrum of compound **8a** (CD_3OD, 100 MHz).

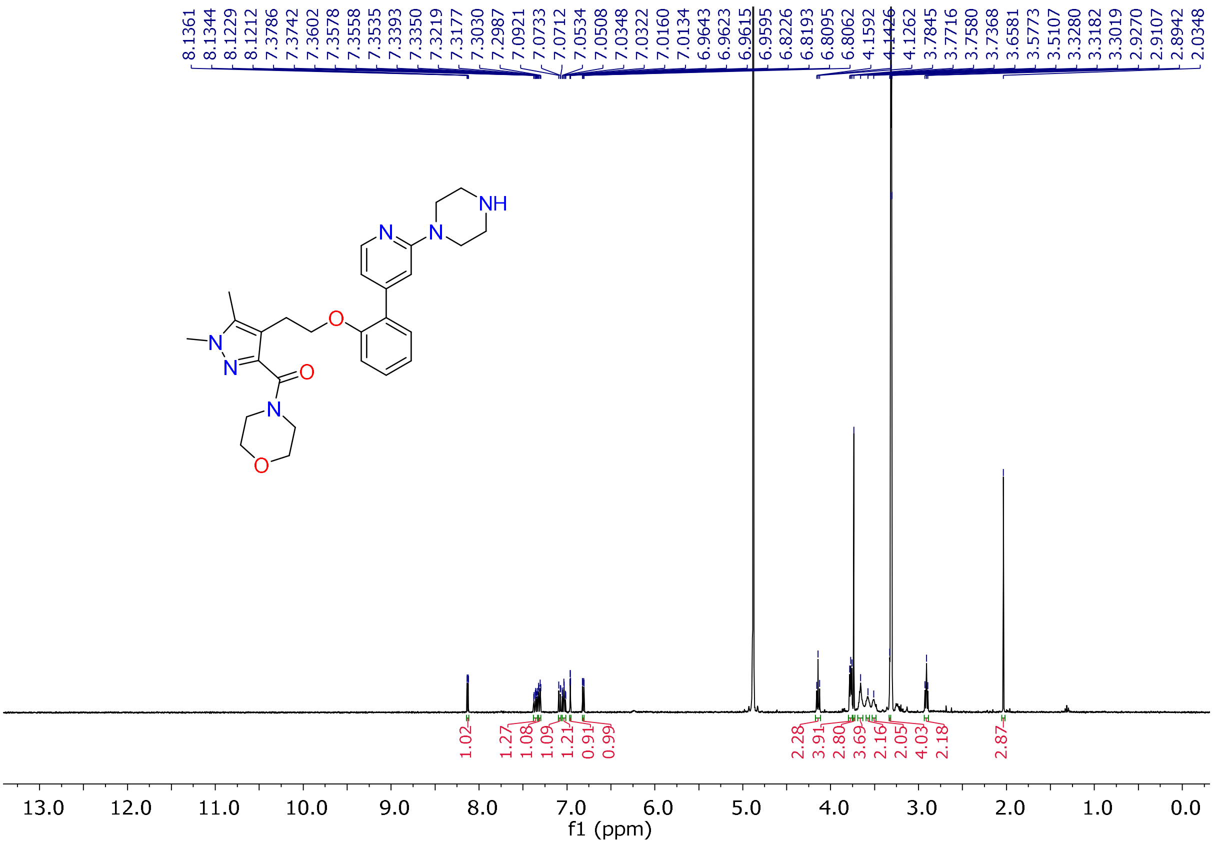

Figure S4. ^{1}H-NMR spectrum of compound **8b** (CD_3OD, 400 MHz).

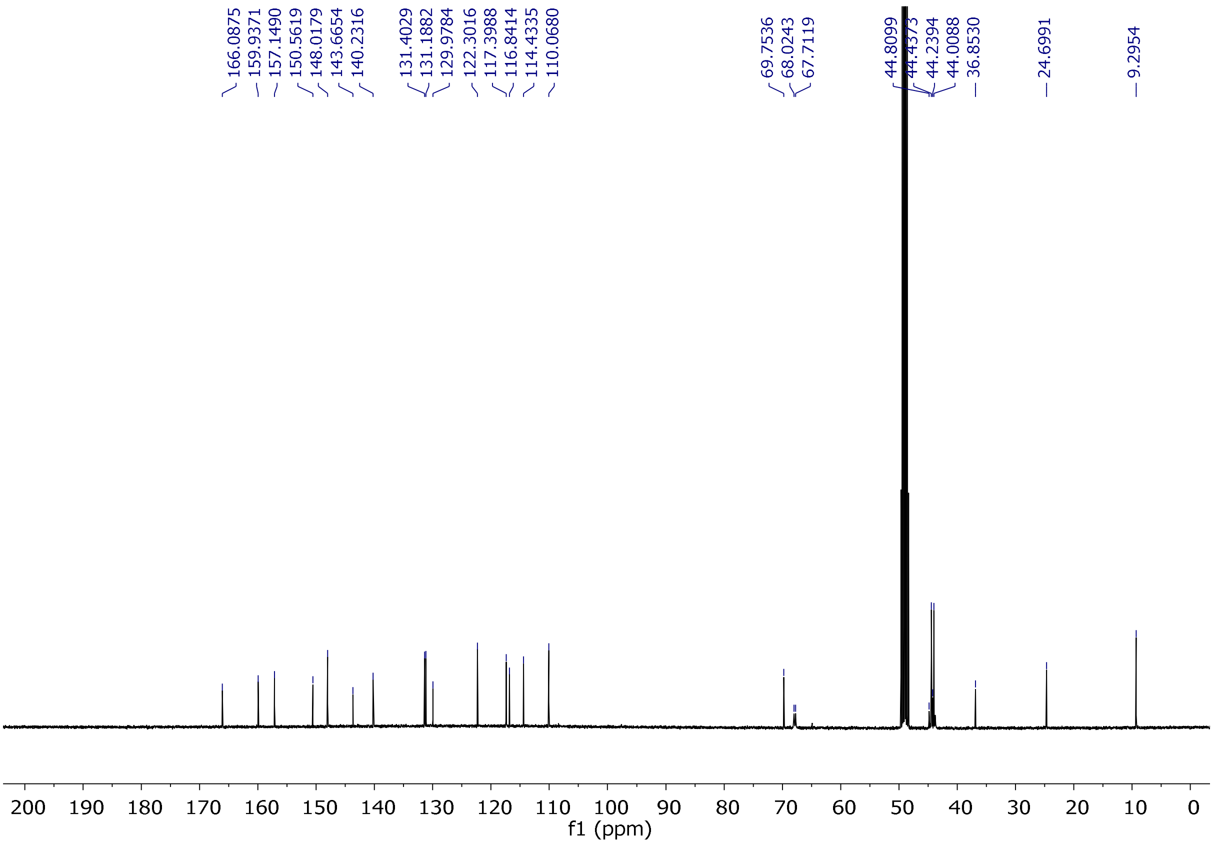

Figure S5. ^{13}C-NMR spectrum of compound **8b** (CD_3OD, 100 MHz).

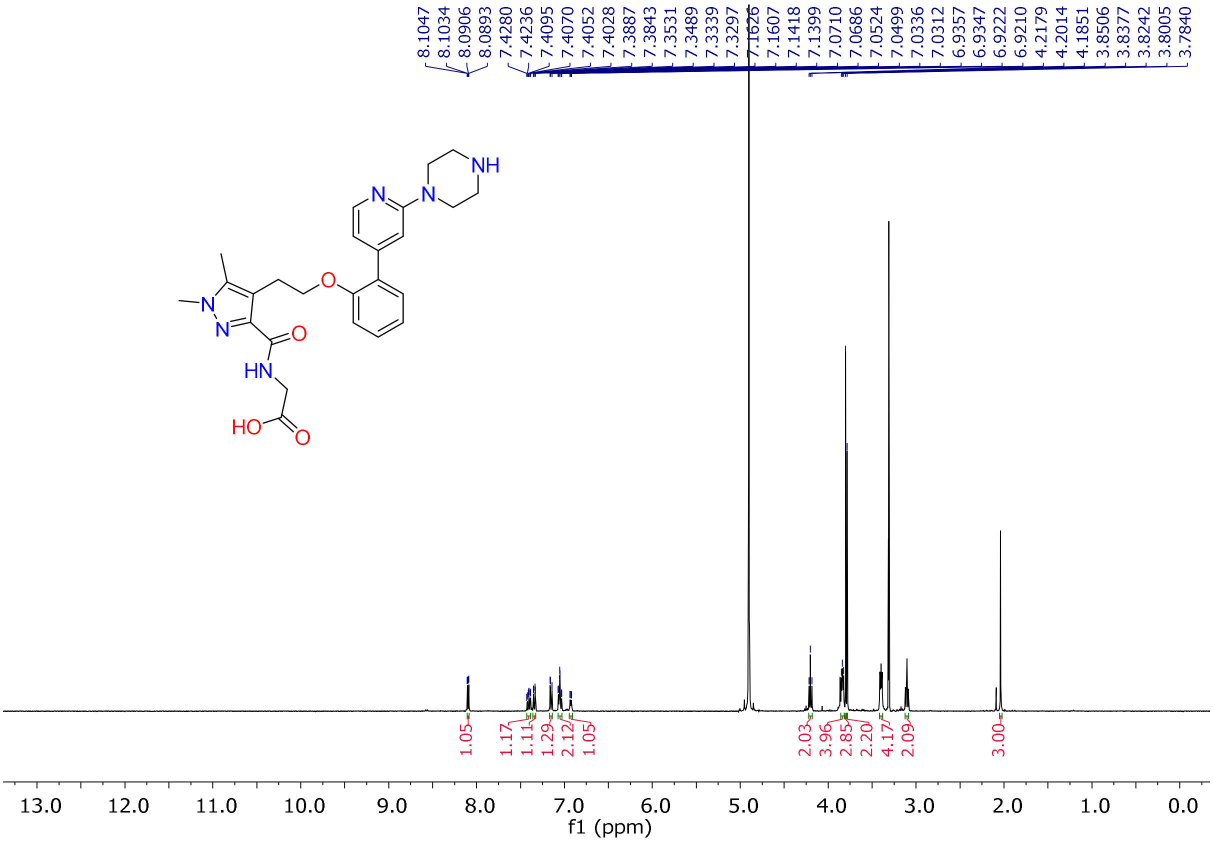

Figure S6. ^{1}H-NMR spectrum of compound **8c** (CD_3OD, 400 MHz).

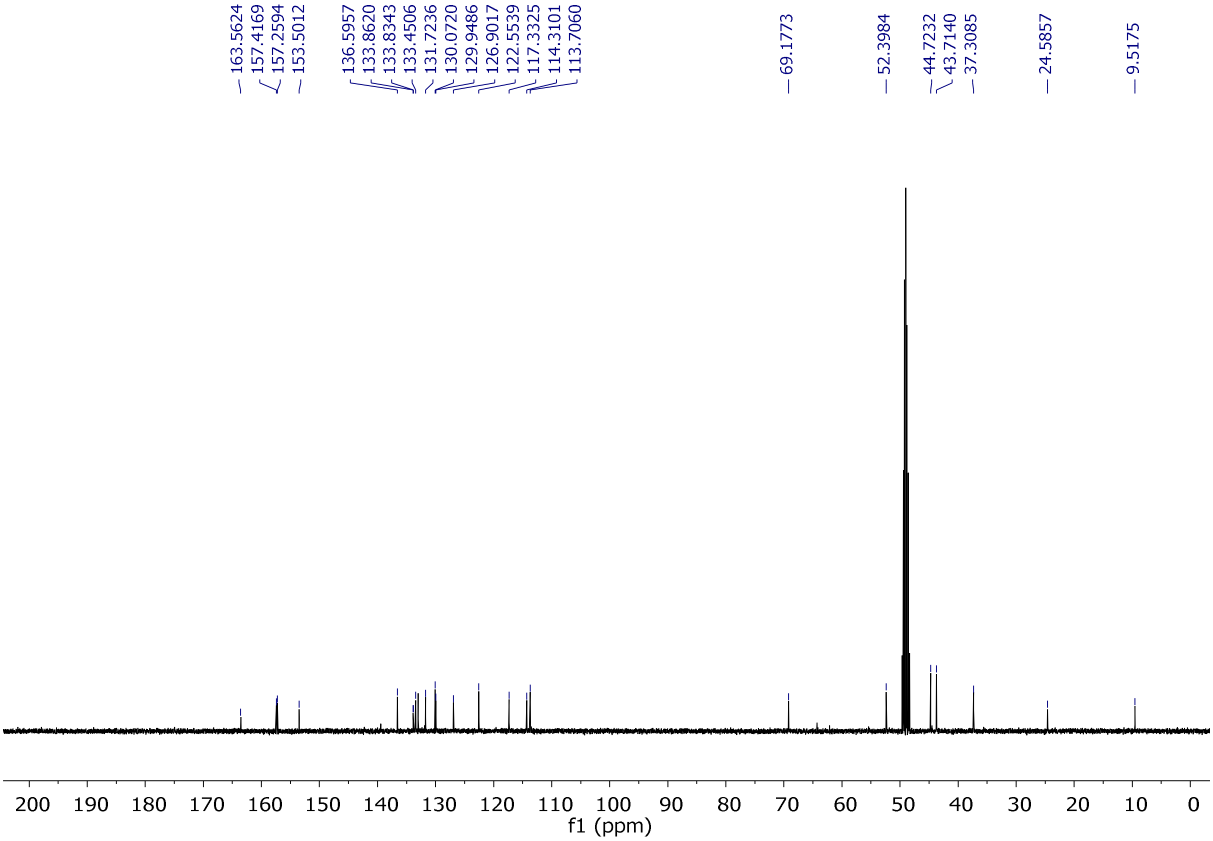

Figure S7. ^{13}C-NMR spectrum of compound **8c** (CD_3OD, 100 MHz).

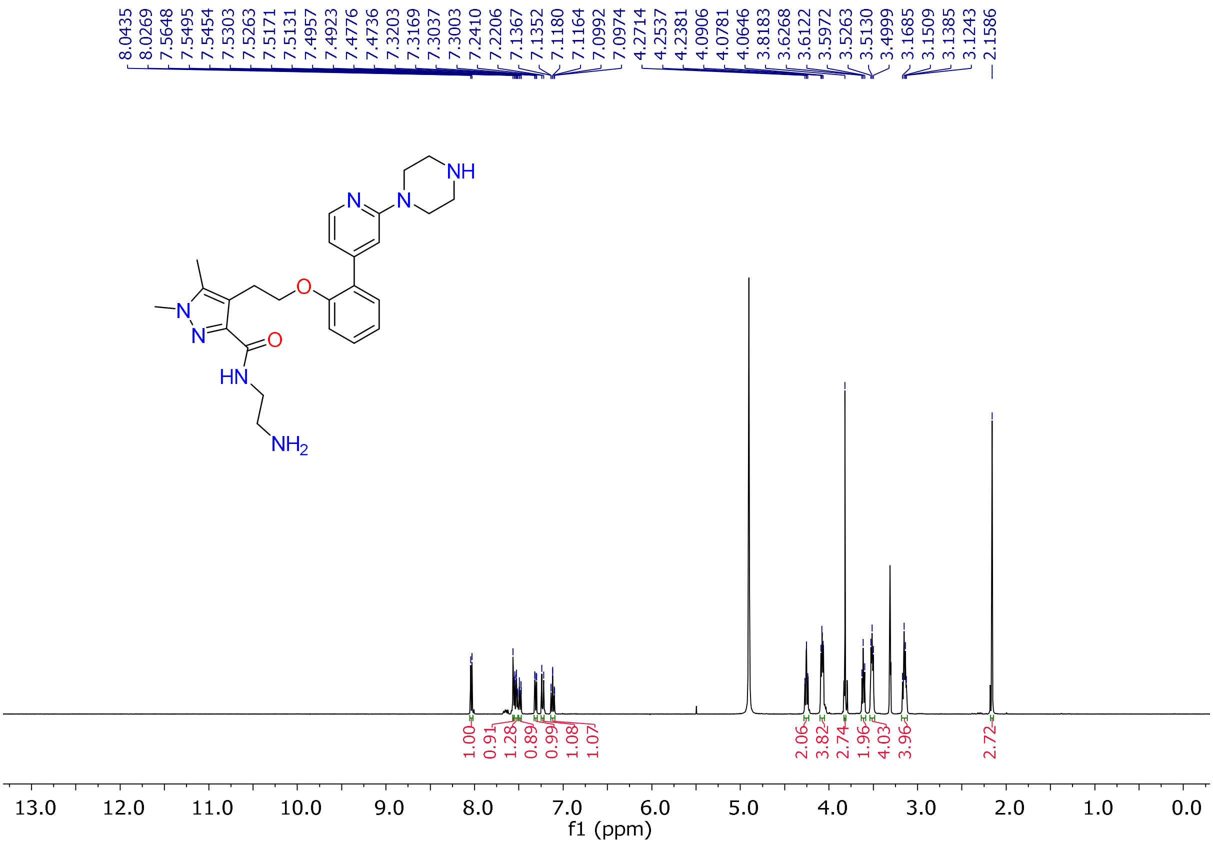

Figure S8. ^{1}H-NMR spectrum of compound **8f** (CD_3OD, 400 MHz).

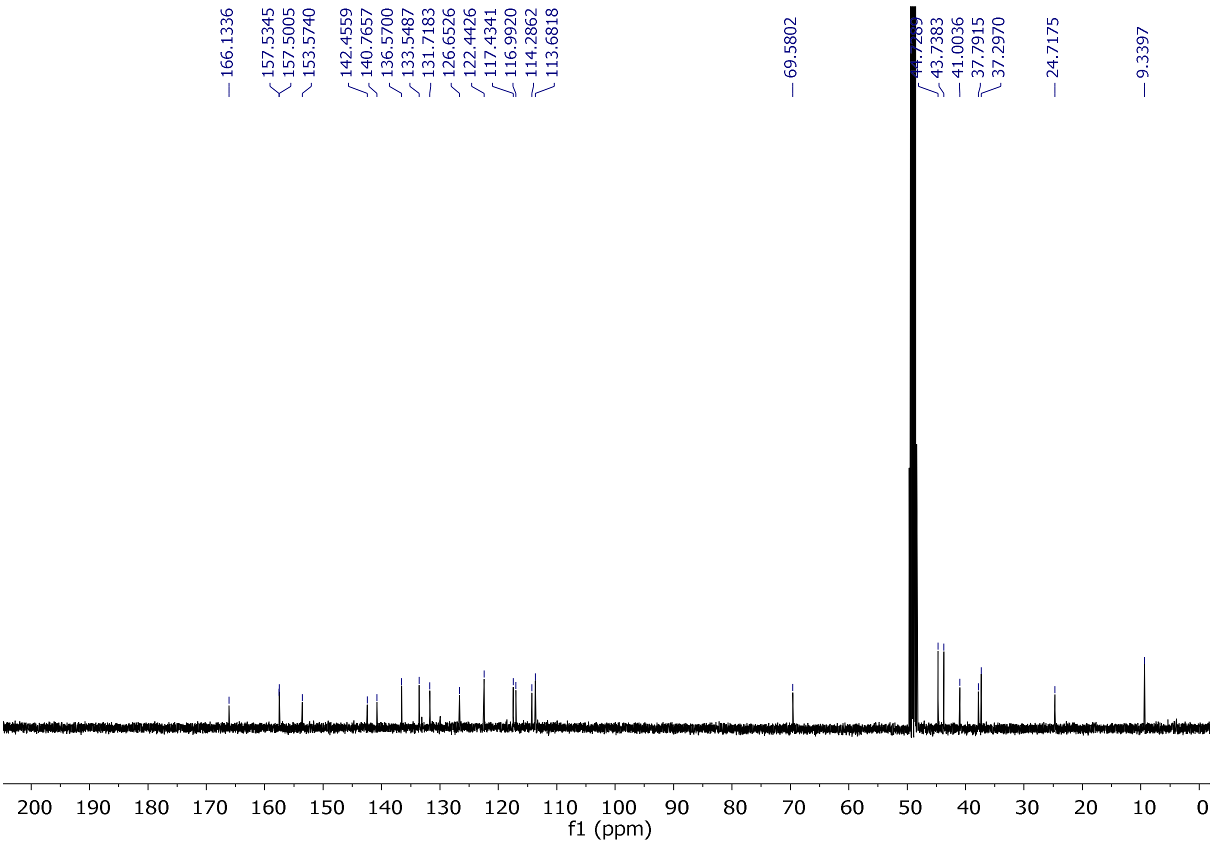

Figure S9. ^{13}C-NMR spectrum of compound **8f** (CD_3OD, 100 MHz).

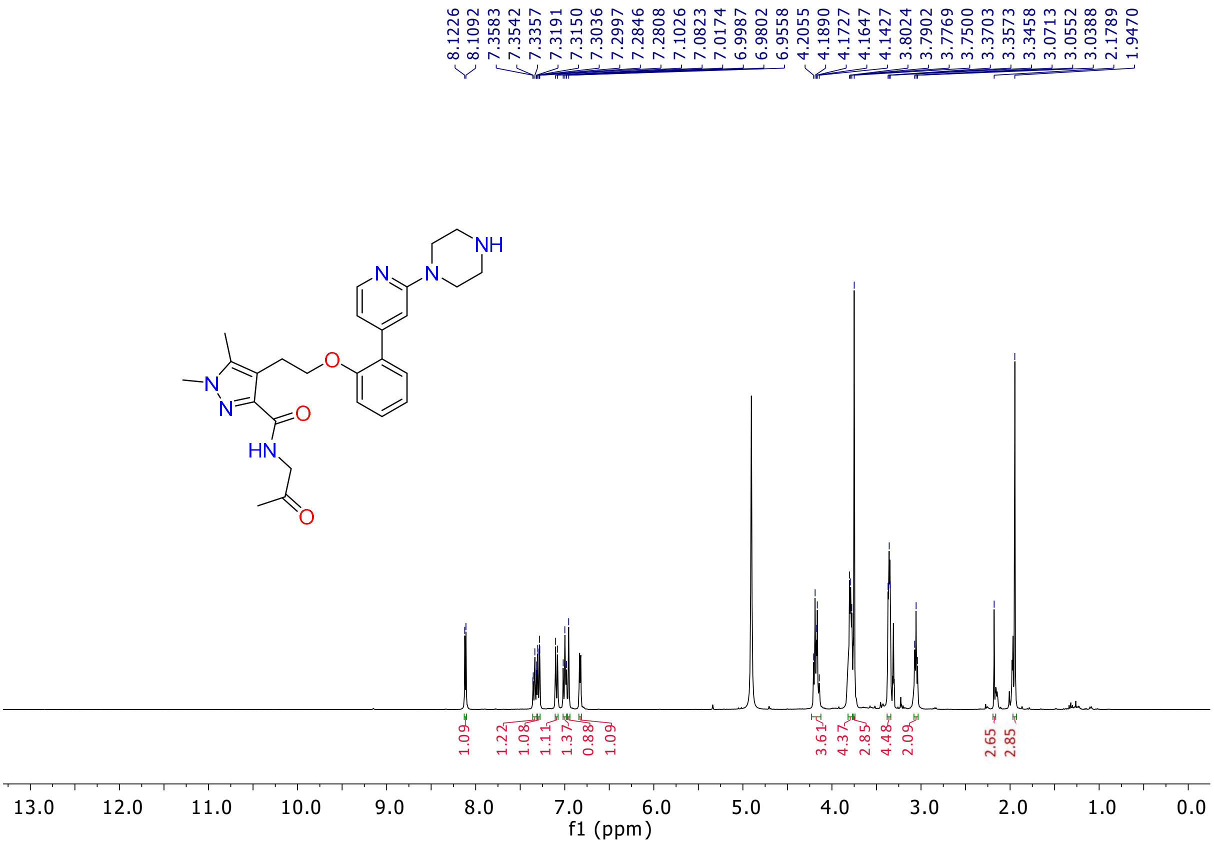

Figure S10. ^{1}H-NMR spectrum of compound **8g** (CD_3OD, 400 MHz).

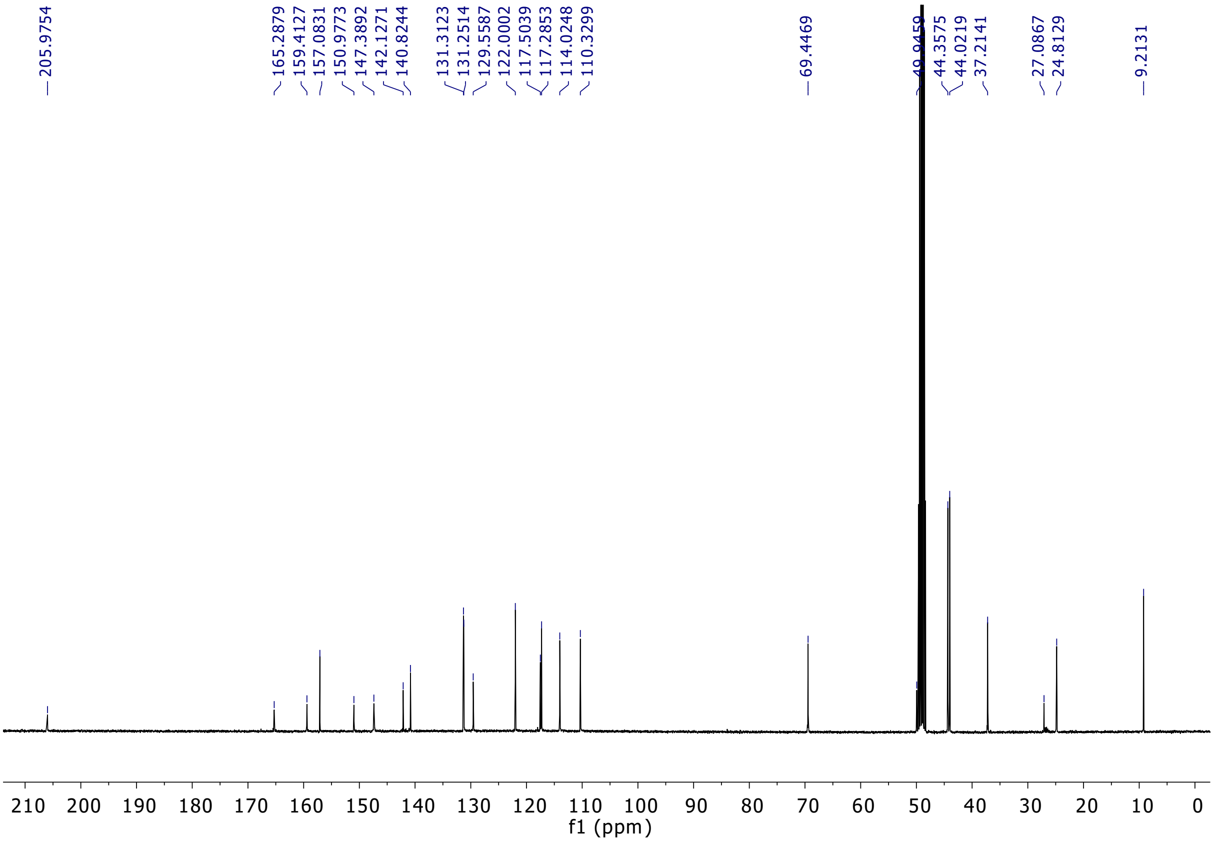

Figure S11. ^{13}C-NMR spectrum of compound **8g** (CD_3OD, 100 MHz).

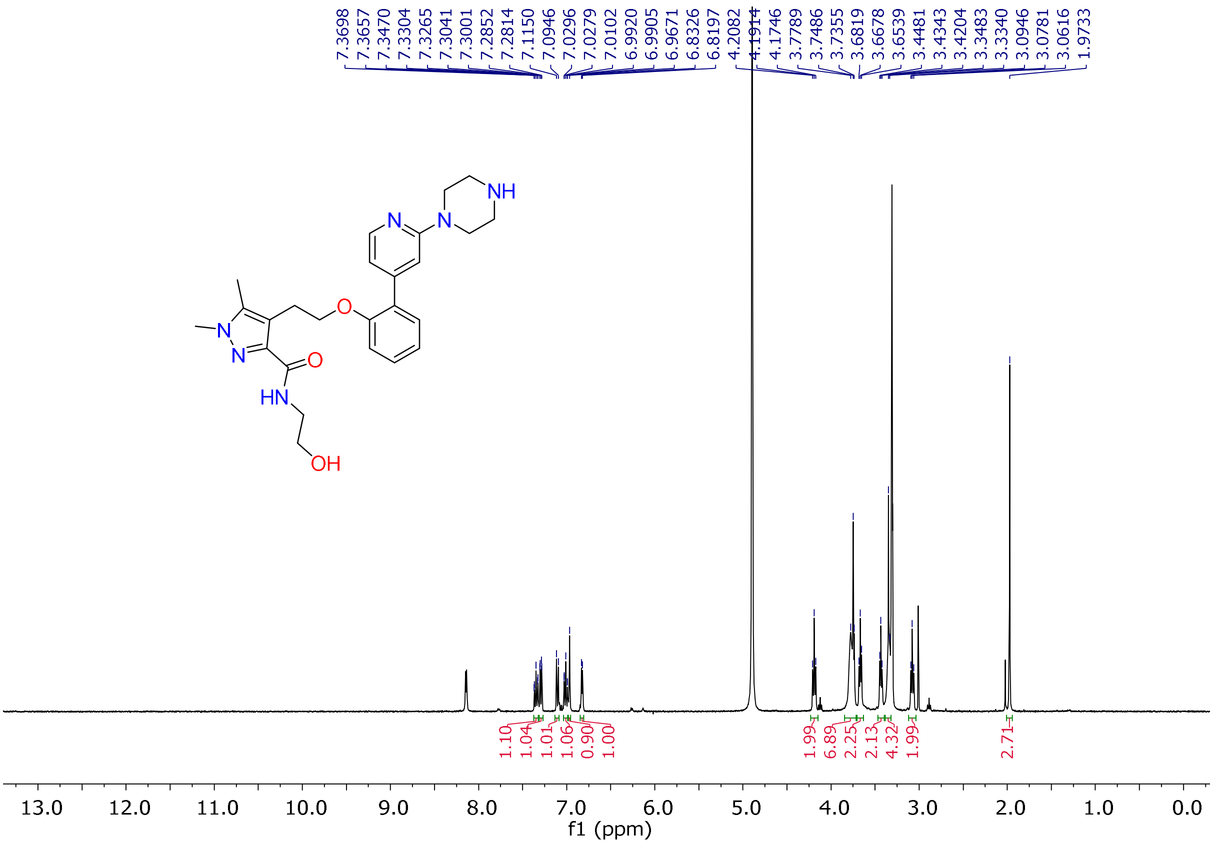

Figure S12. ^{1}H-NMR spectrum of compound **8h** (CD_3OD, 400 MHz).

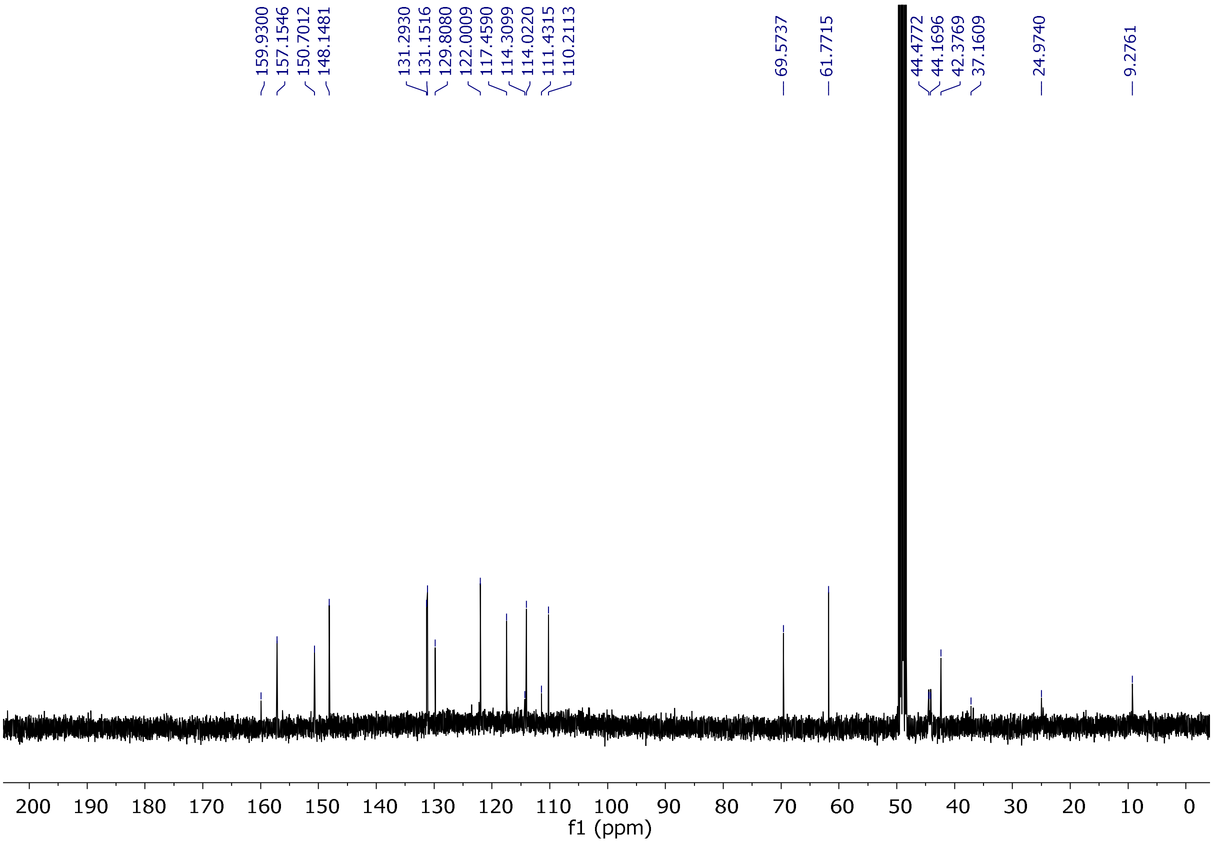

Figure S13. ^{13}C-NMR spectrum of compound **8h** (CD_3OD, 100 MHz).

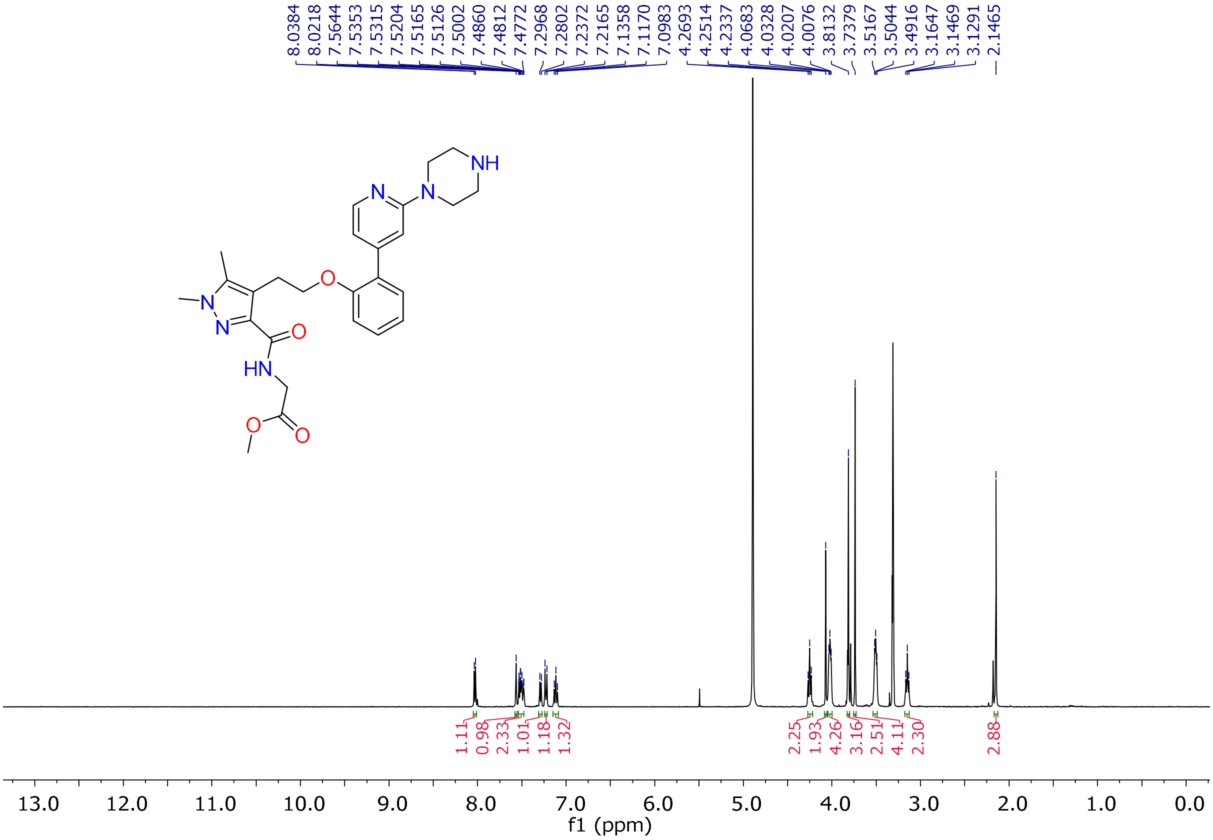

Figure S14. ^{1}H-NMR spectrum of compound **8i** (CD_3OD, 400 MHz).

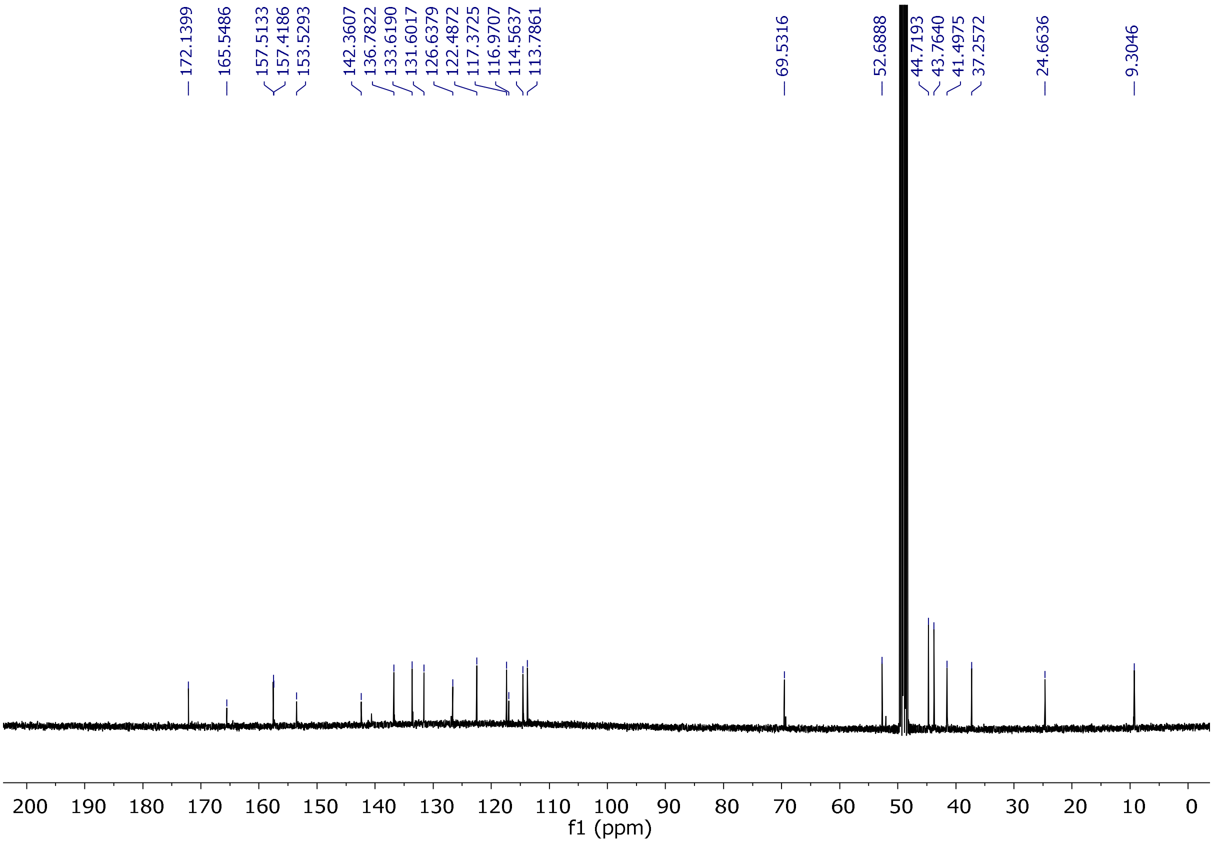

Figure S15. ^{13}C-NMR spectrum of compound **8i** (CD_3OD, 100 MHz).

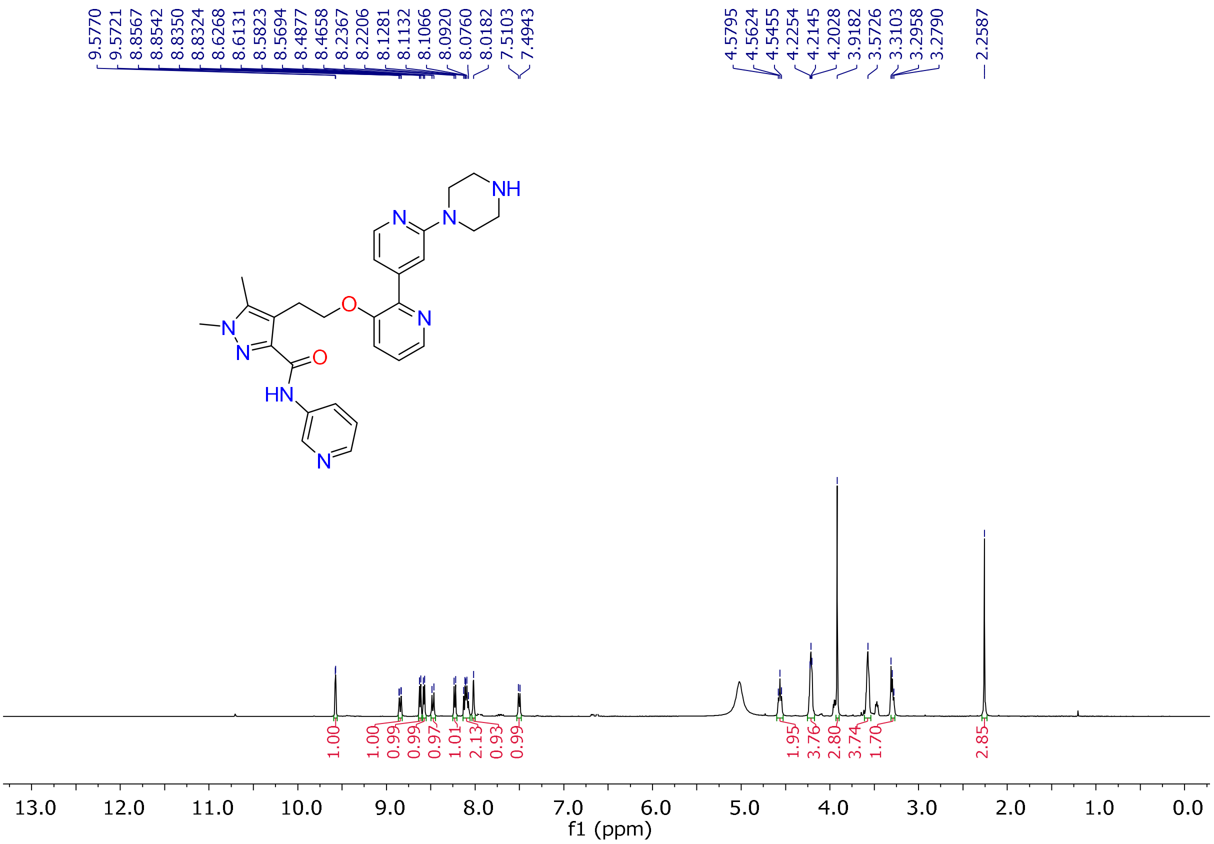

Figure S16. ^{1}H-NMR spectrum of compound **9a** (CD_3OD, 400 MHz).

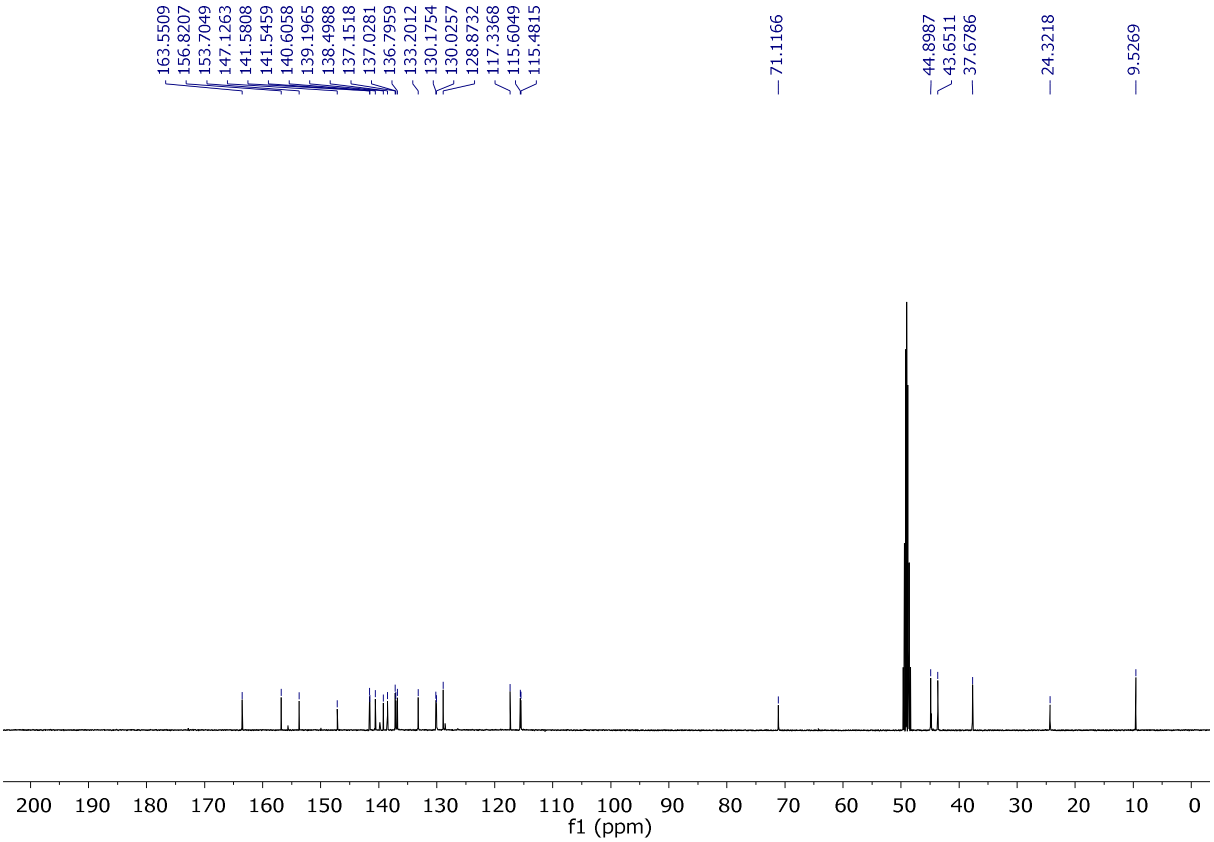

Figure S17. ^{13}C-NMR spectrum of compound **9a** (CD_3OD, 100 MHz).

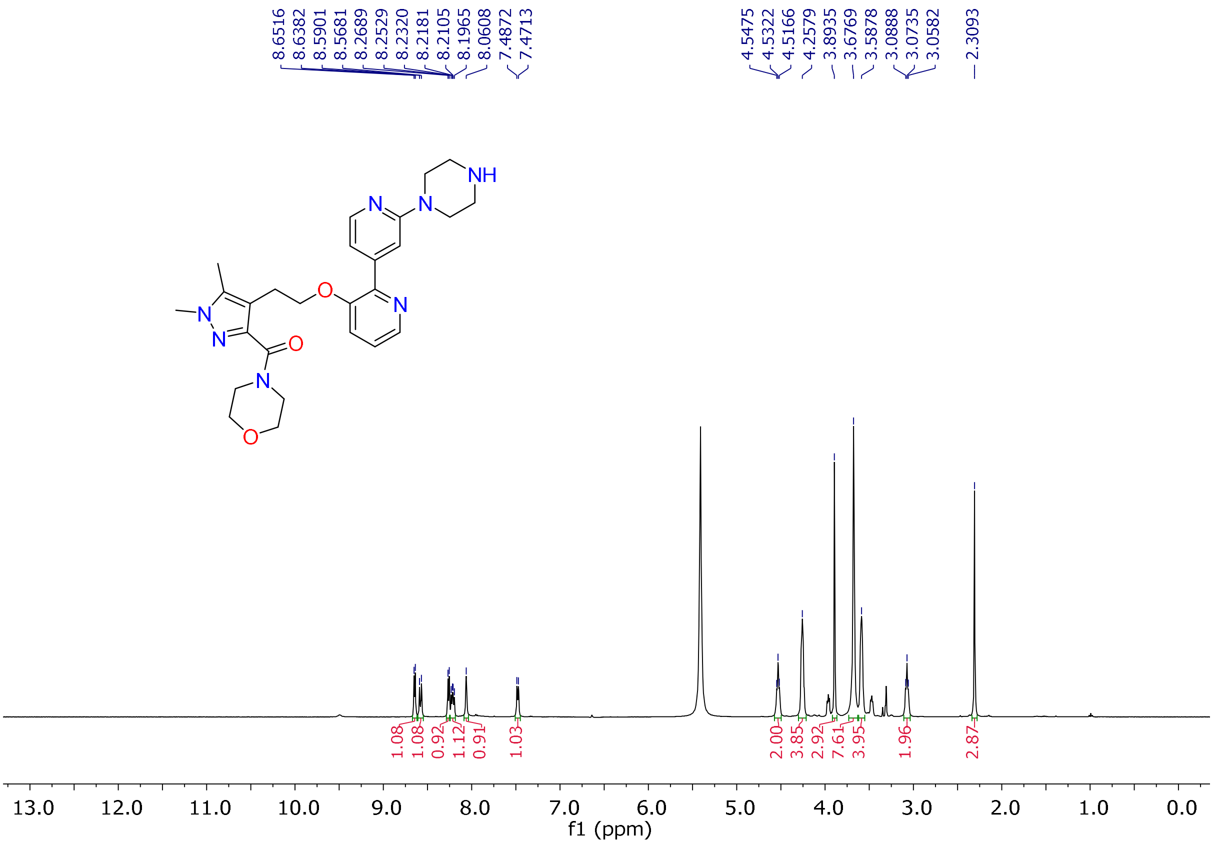

Figure S18. ¹H-NMR spectrum of compound **9b** (CD_3OD, 400 MHz).

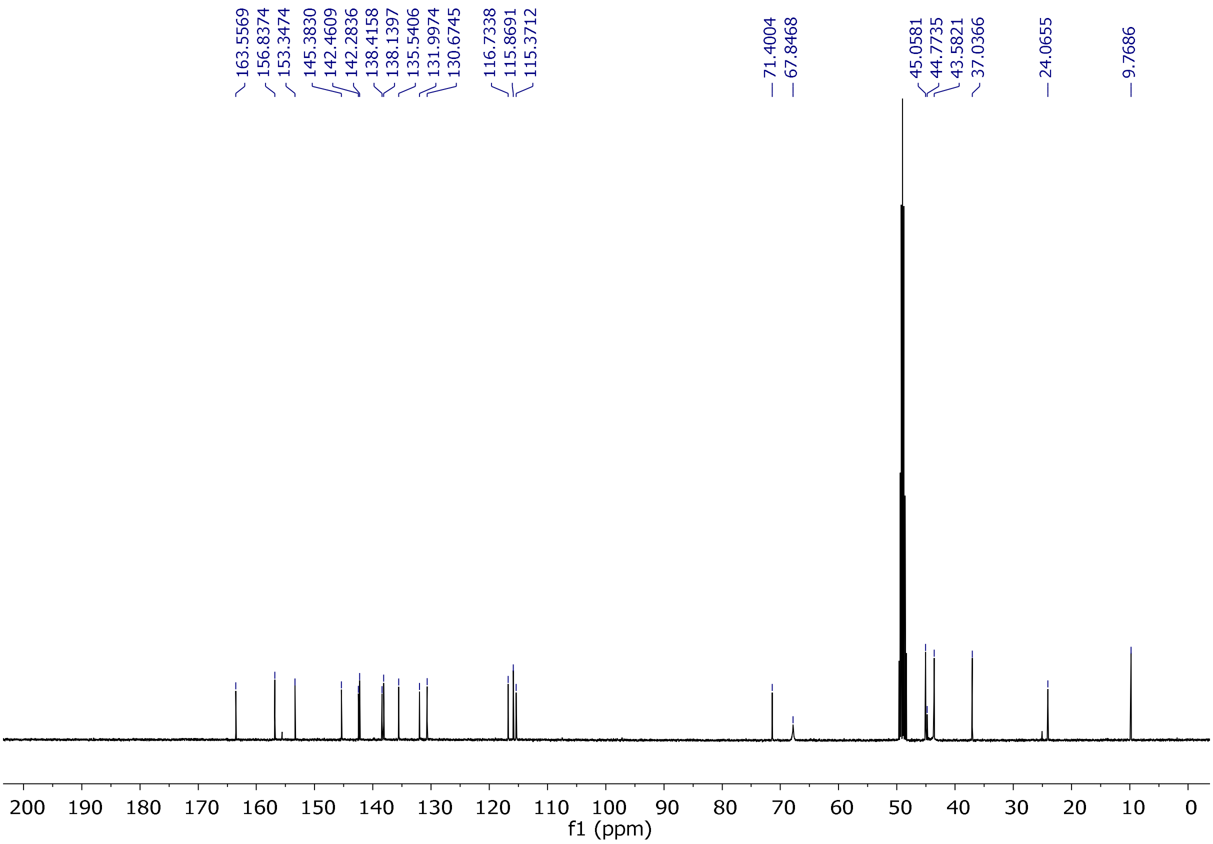

Figure S19. ¹³C-NMR spectrum of compound **9b** (CD_3OD, 100 MHz).

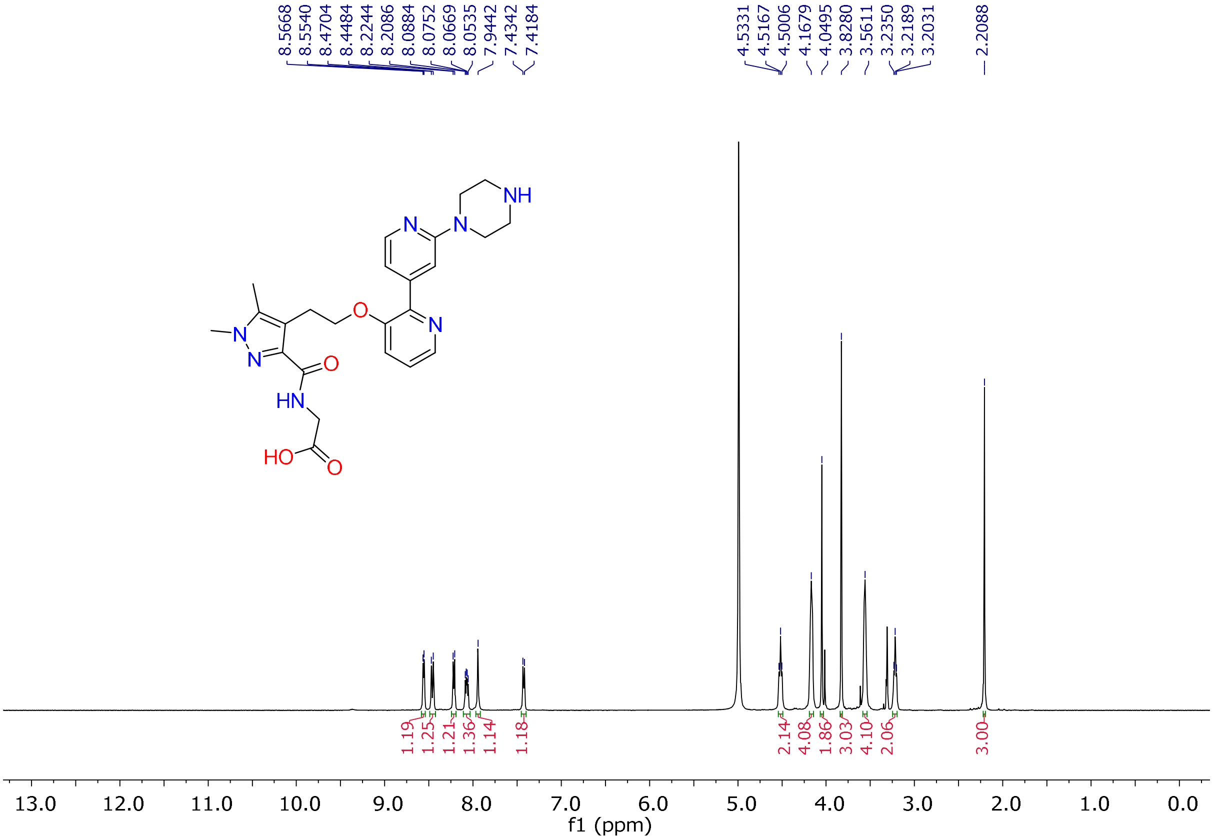

Figure S20. ^{1}H-NMR spectrum of compound **9c** (CD_3OD, 400 MHz).

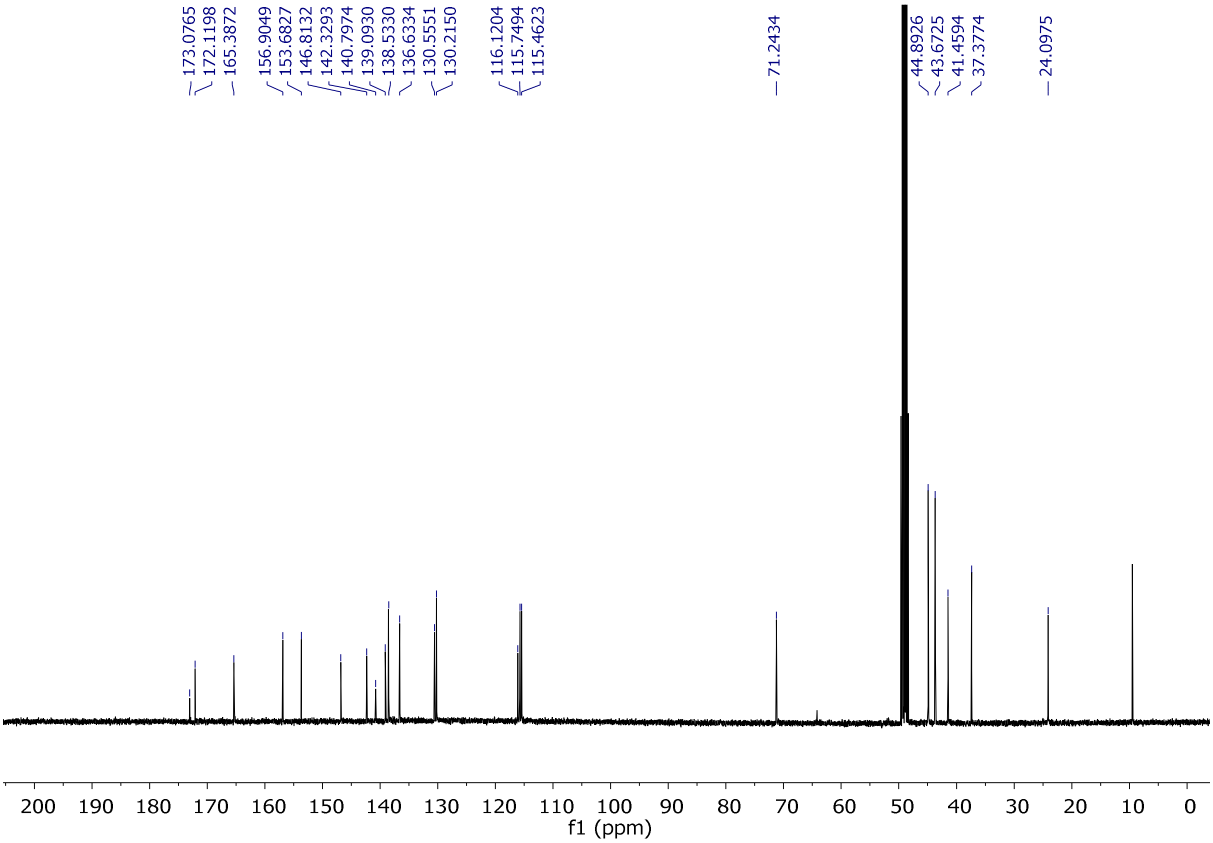

Figure S21. ^{13}C-NMR spectrum of compound **9c** (CD_3OD, 100 MHz).

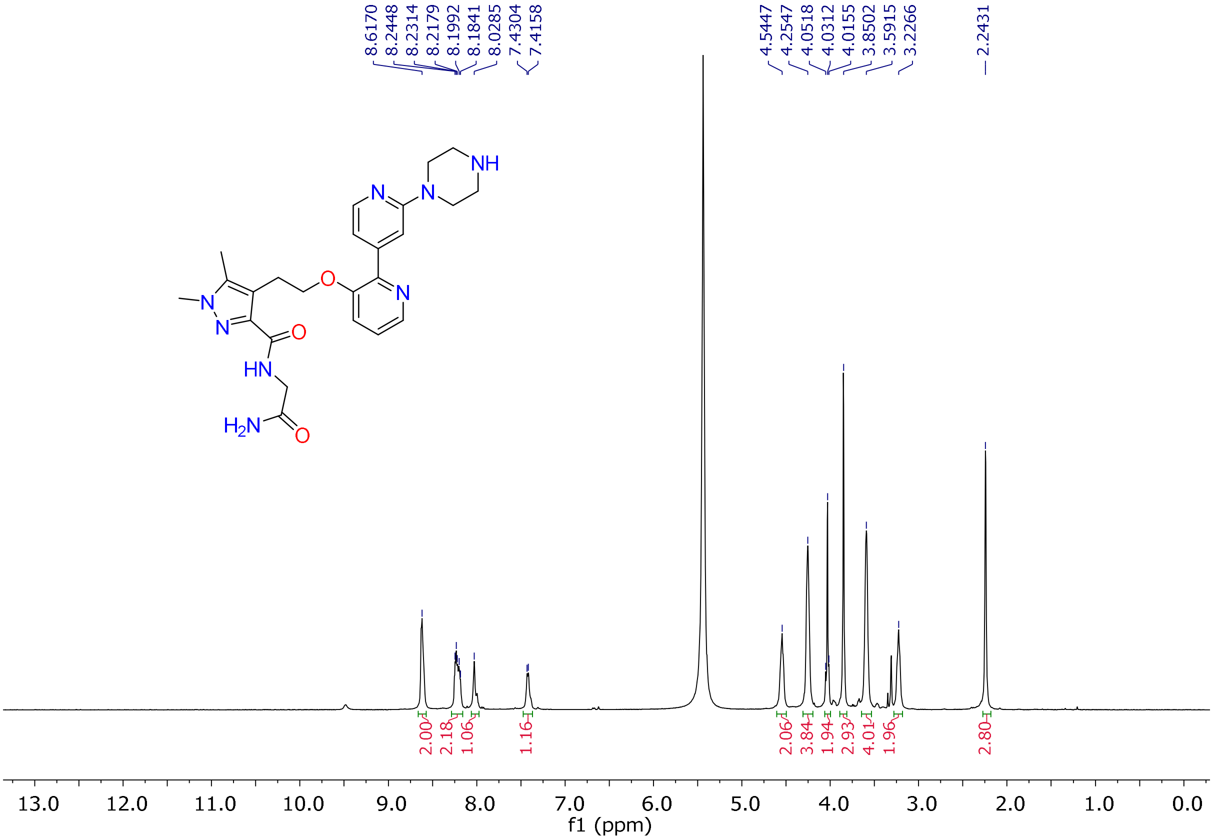

Figure S22. ^{1}H-NMR spectrum of compound **9d** (CD_3OD, 400 MHz).

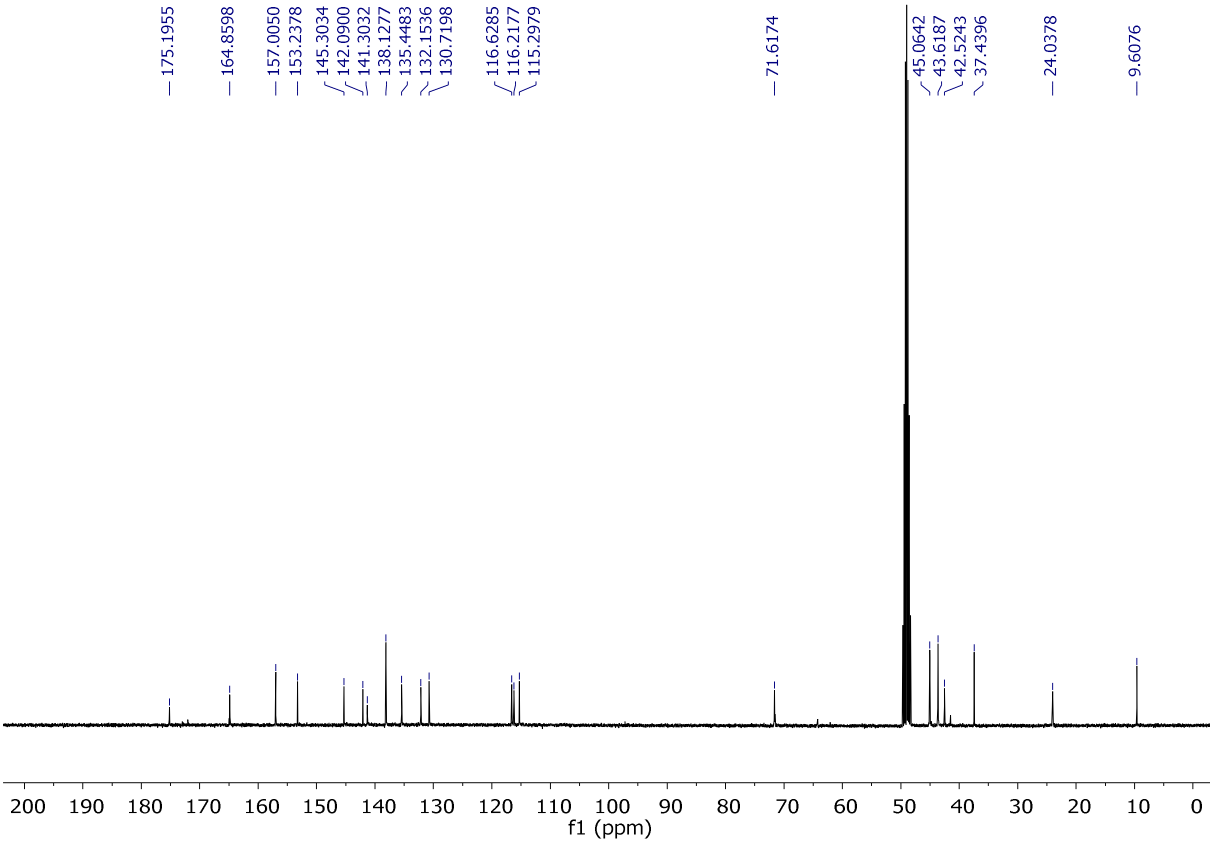

Figure S23. ^{13}C-NMR spectrum of compound **9d** (CD_3OD, 100 MHz).

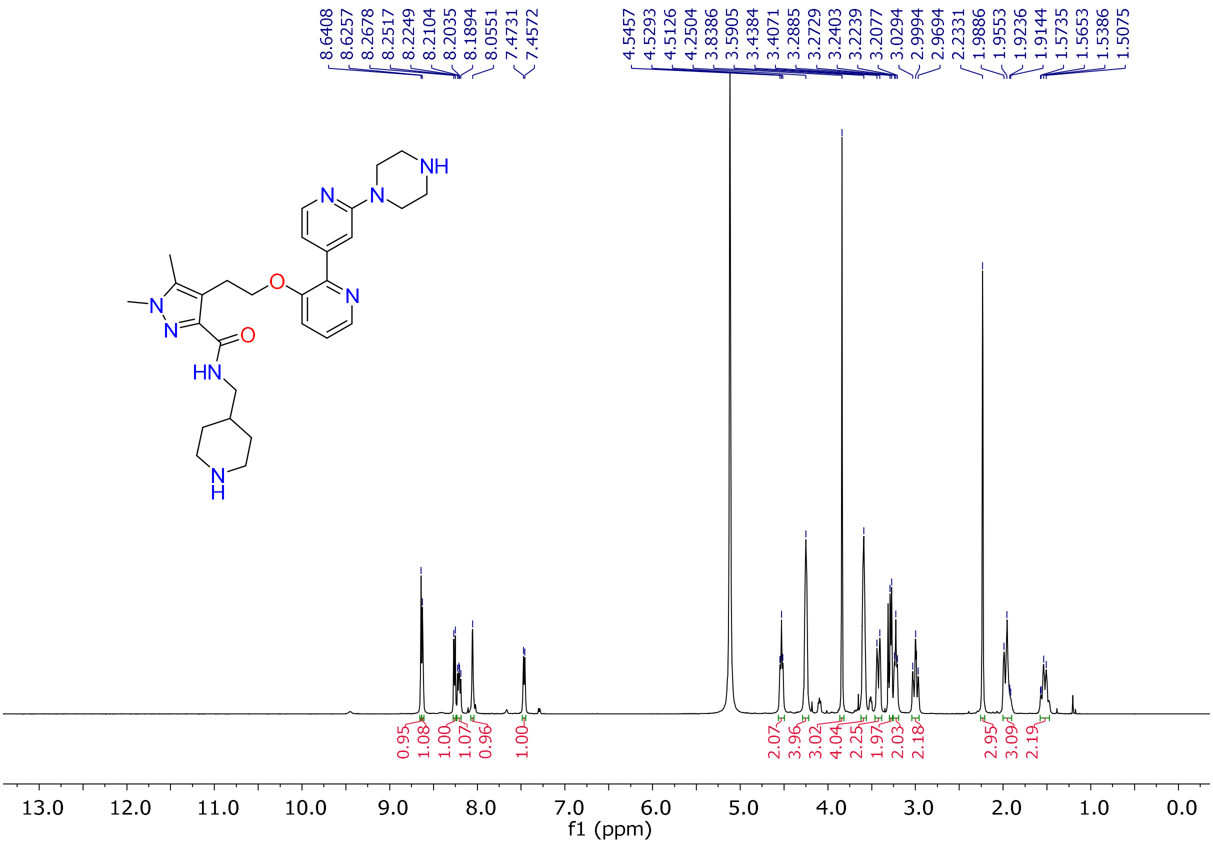

Figure S24. ^{1}H-NMR spectrum of compound **9e** (CD_3OD, 400 MHz).

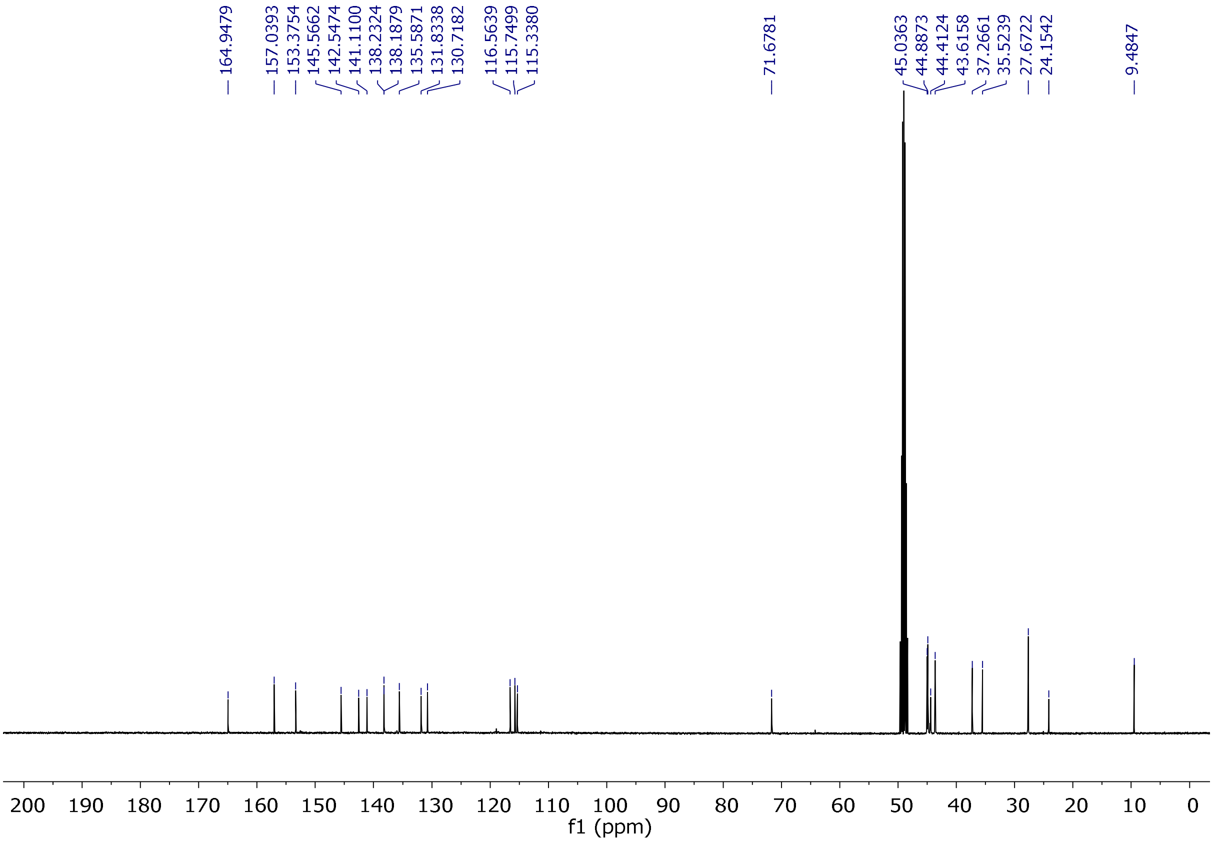

Figure S25. ^{13}C-NMR spectrum of compound **9e** (CD_3OD, 100 MHz).

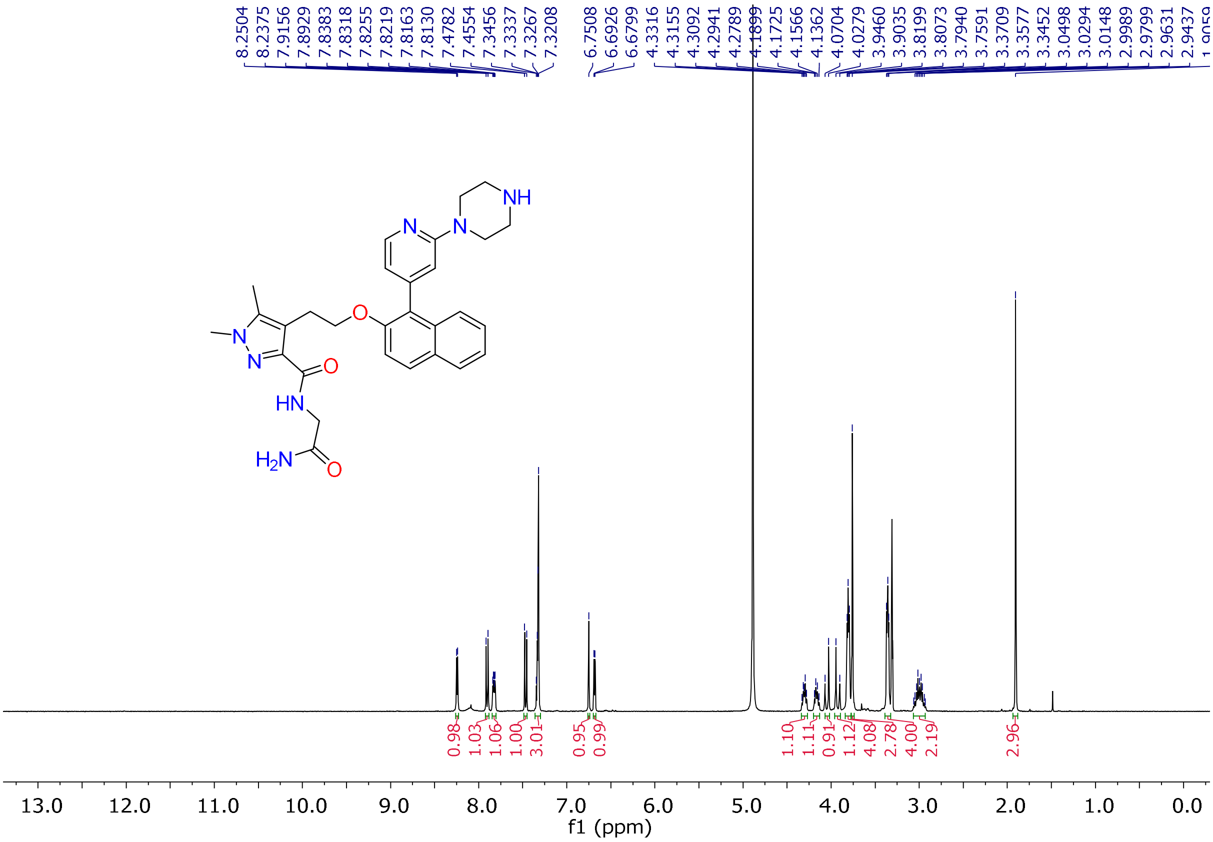

Figure S26. ^{1}H-NMR spectrum of compound **10a** (CD_3OD, 400 MHz).

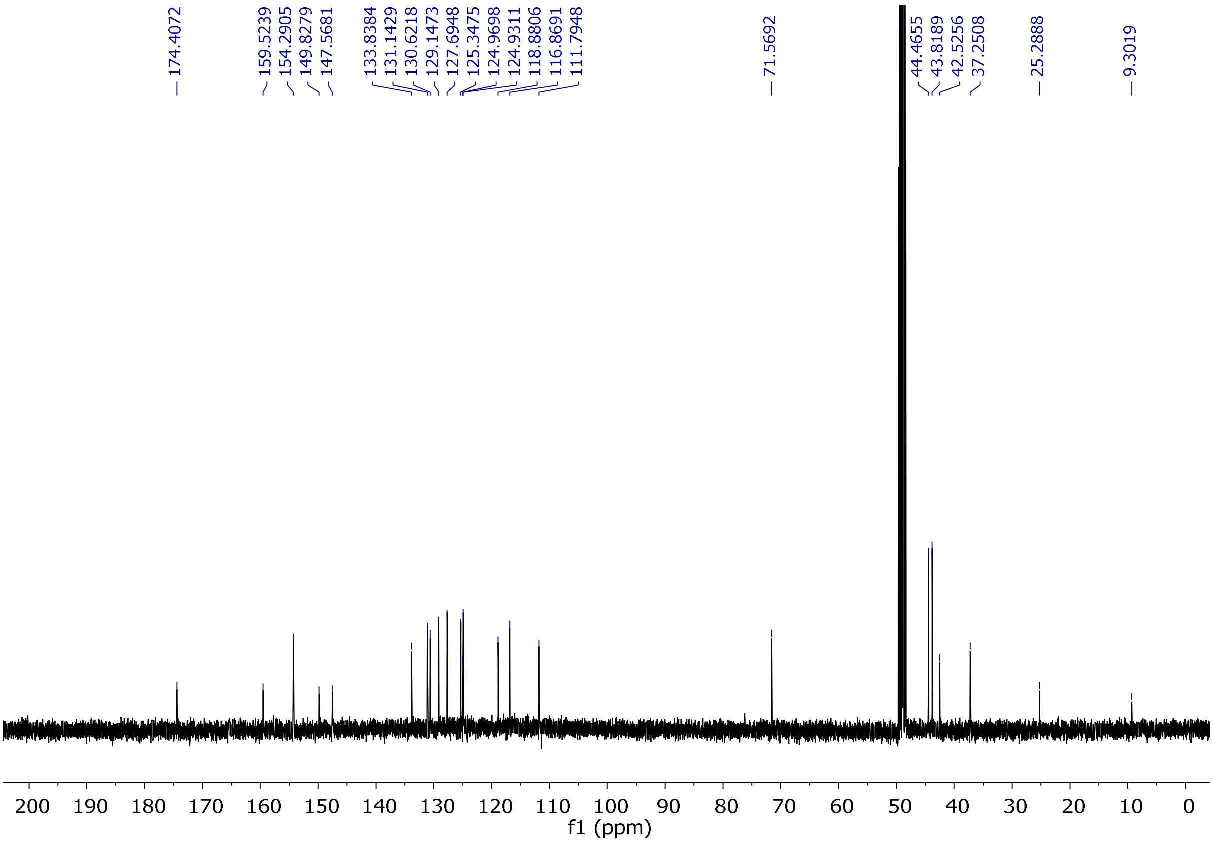

Figure S27. ^{13}C-NMR spectrum of compound **10a** (CD_3OD, 100 MHz).

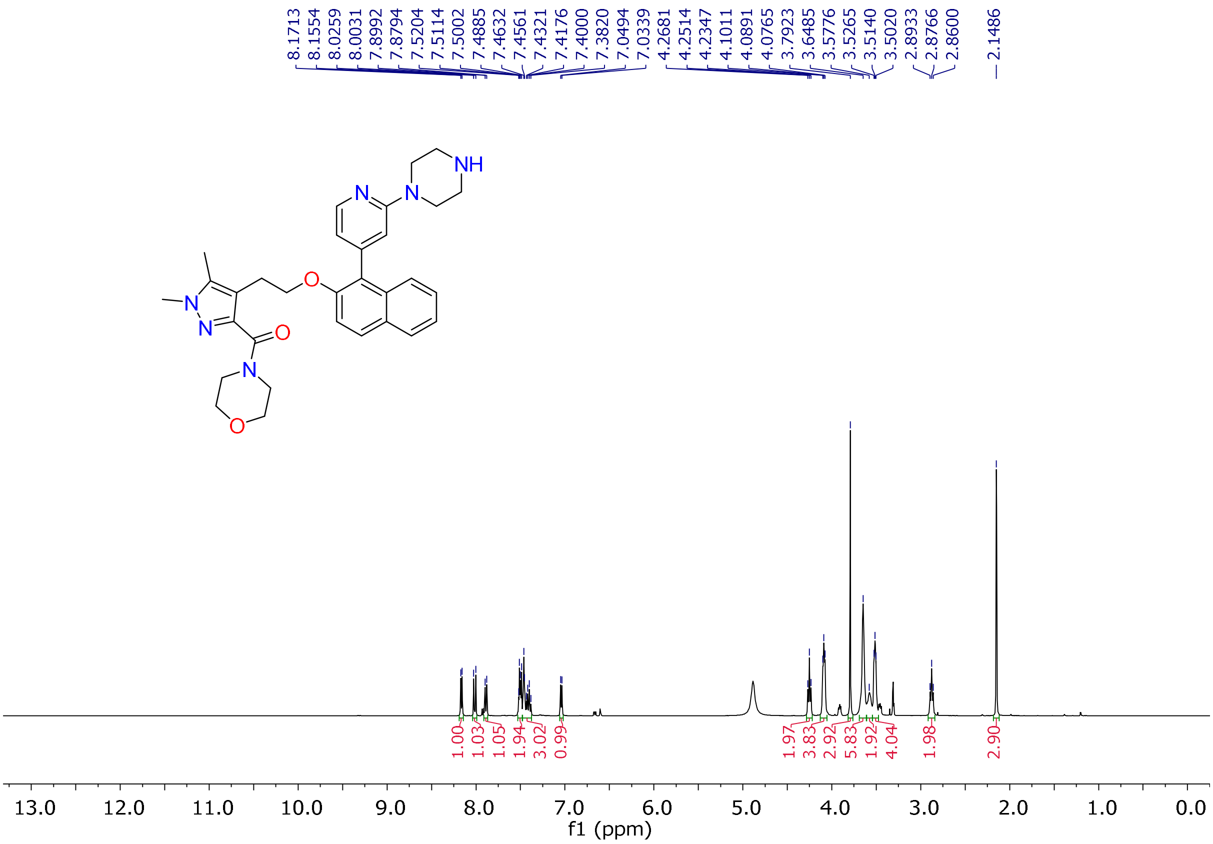

Figure S28. ^{1}H-NMR spectrum of compound **10b** (CD_3OD, 400 MHz).

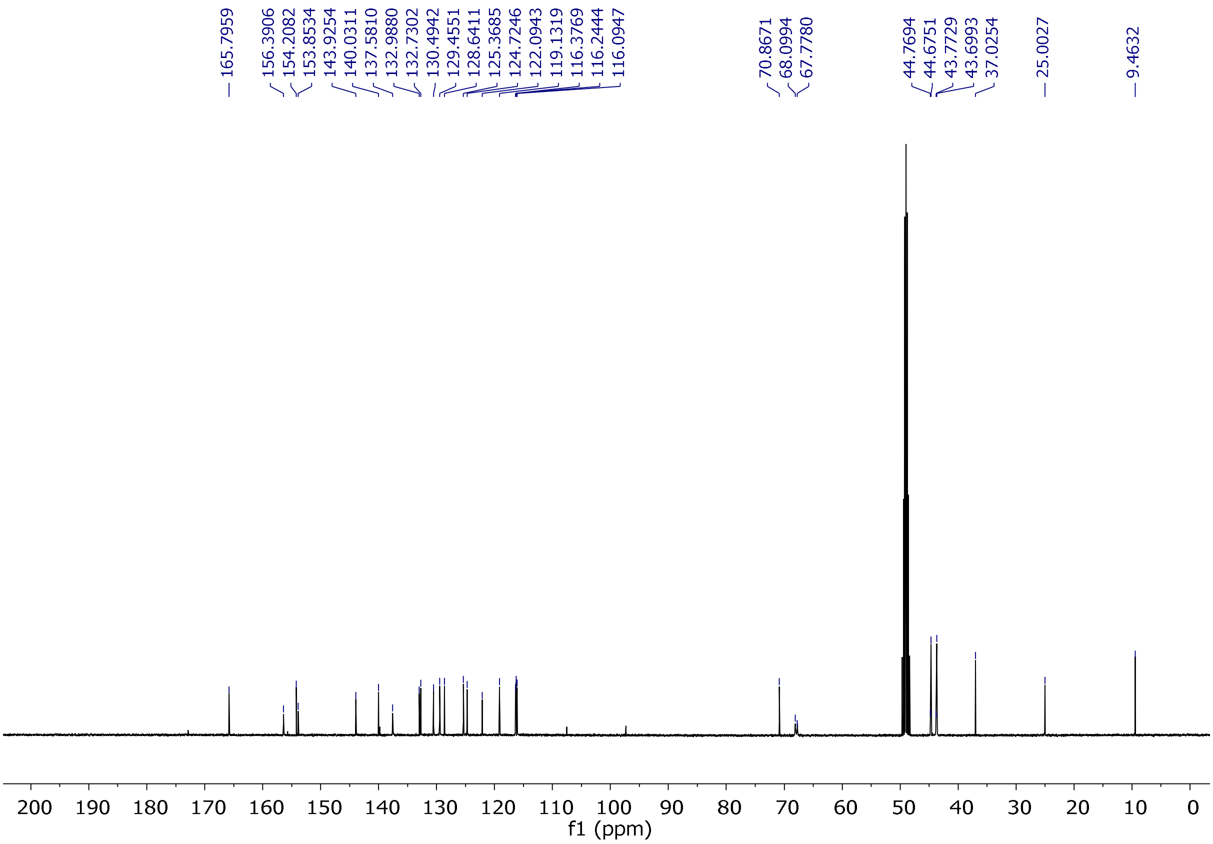

Figure S29. ^{13}C-NMR spectrum of compound **10b** (CD_3OD, 100 MHz).

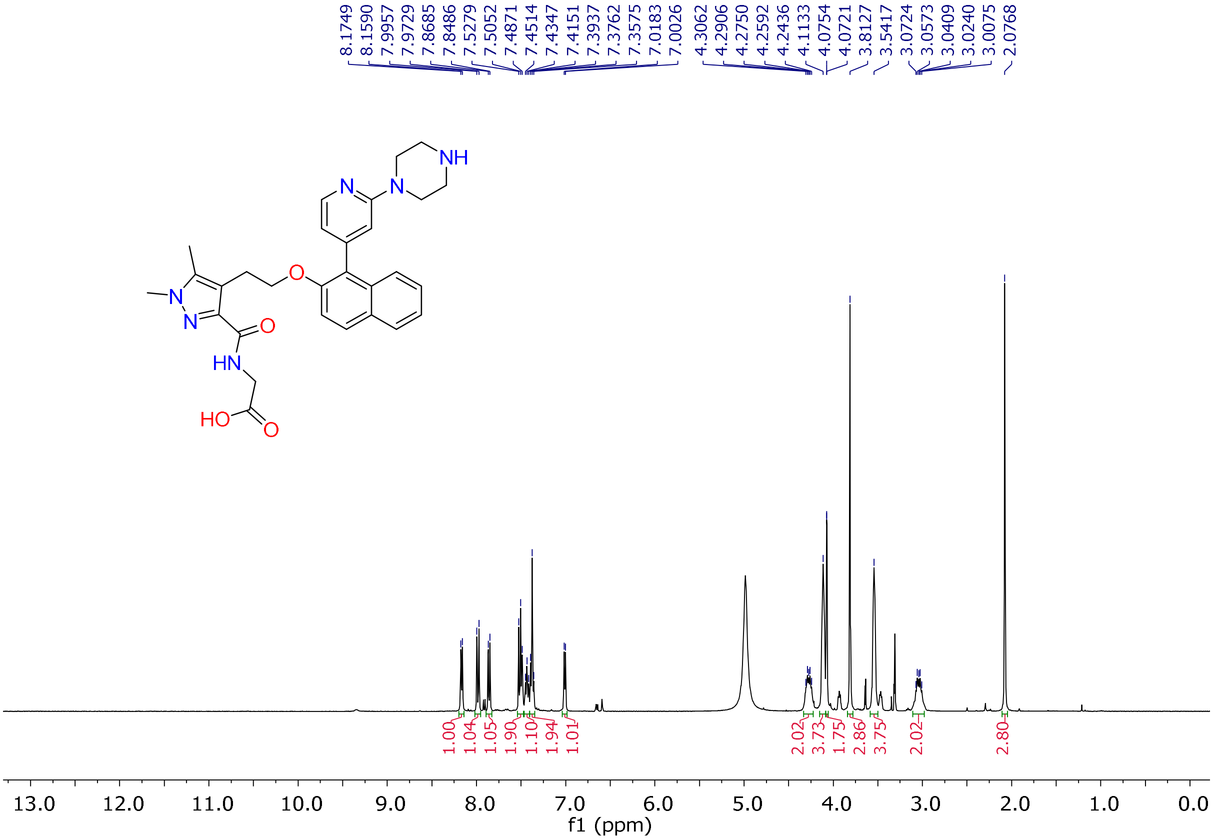

Figure S30. ^{1}H-NMR spectrum of compound **10c** (CD_3OD, 400 MHz).

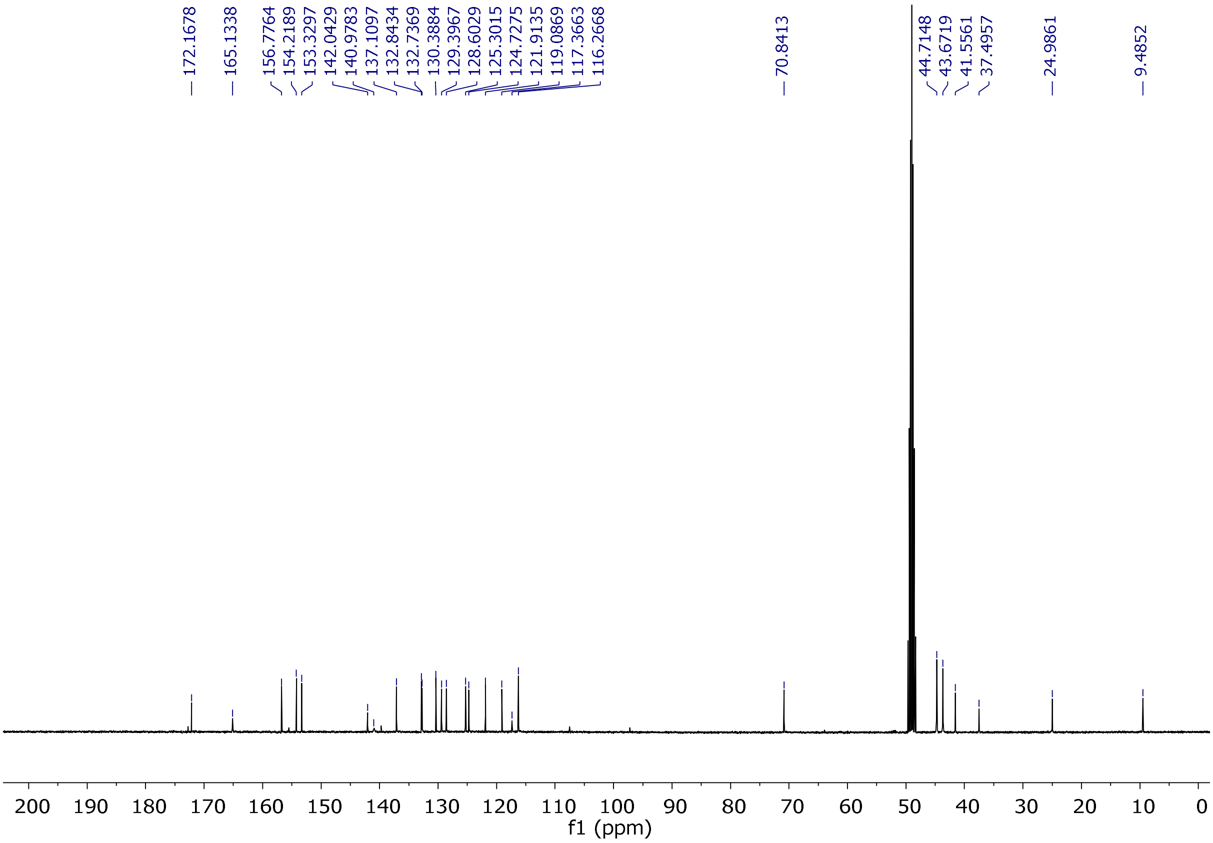

Figure S31. ^{13}C-NMR spectrum of compound **10c** (CD_3OD, 100 MHz).

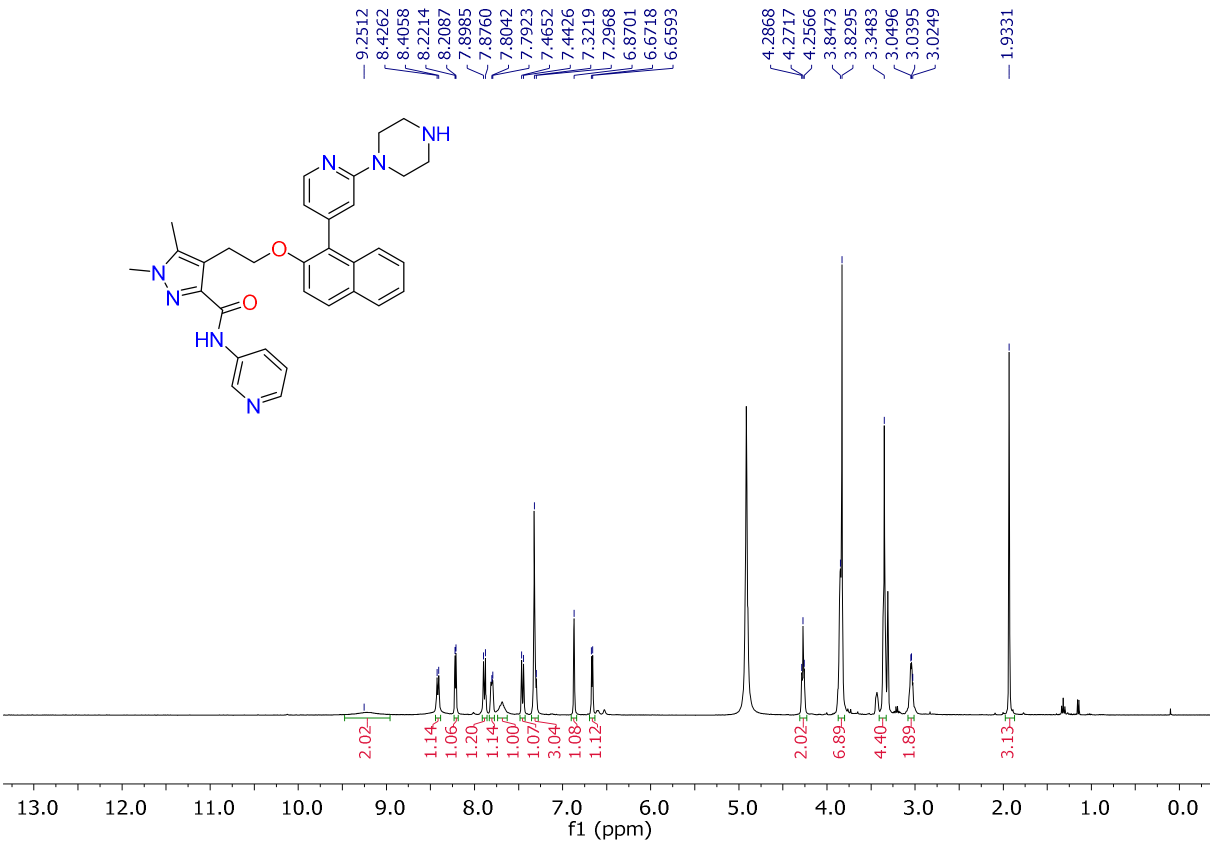

Figure S32. ^{1}H-NMR spectrum of compound **10d** (CD_3OD, 400 MHz).

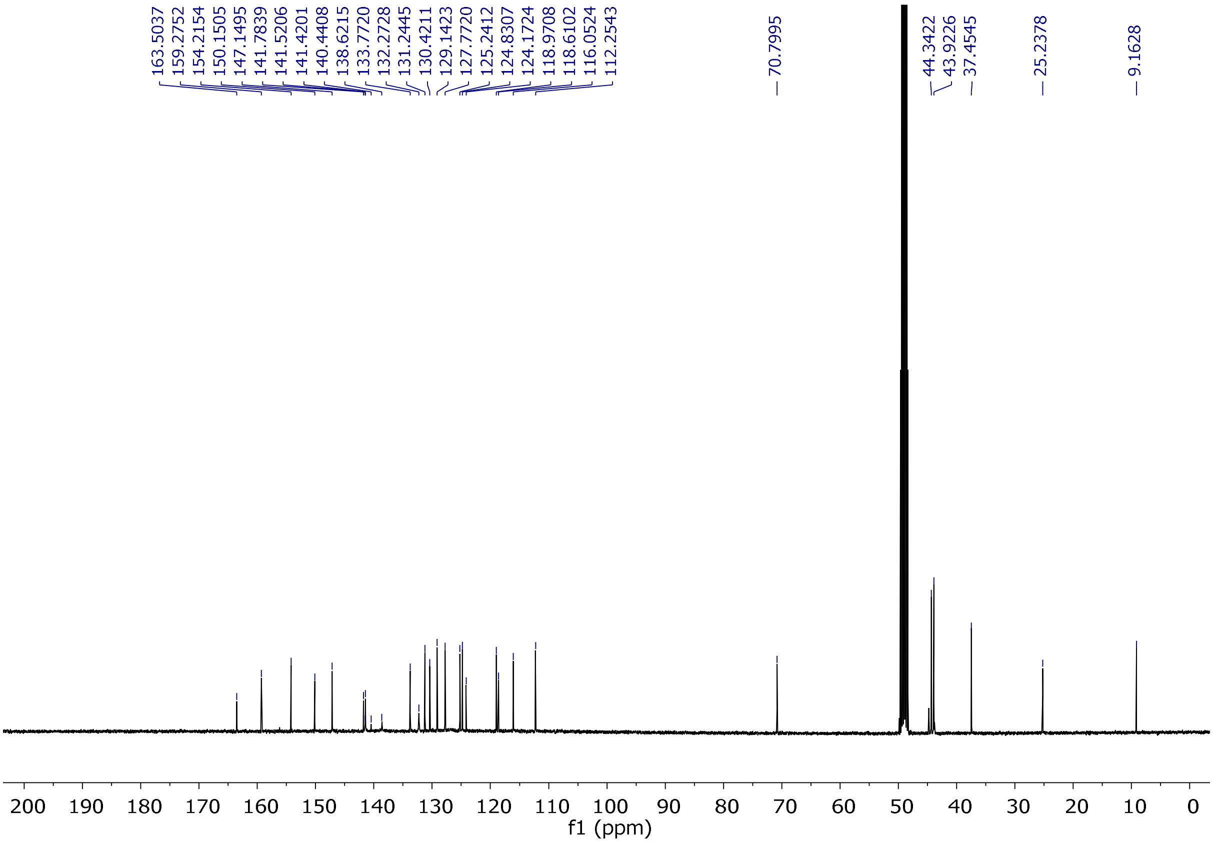

Figure S33. ^{13}C-NMR spectrum of compound **10d** (CD_3OD, 100 MHz).

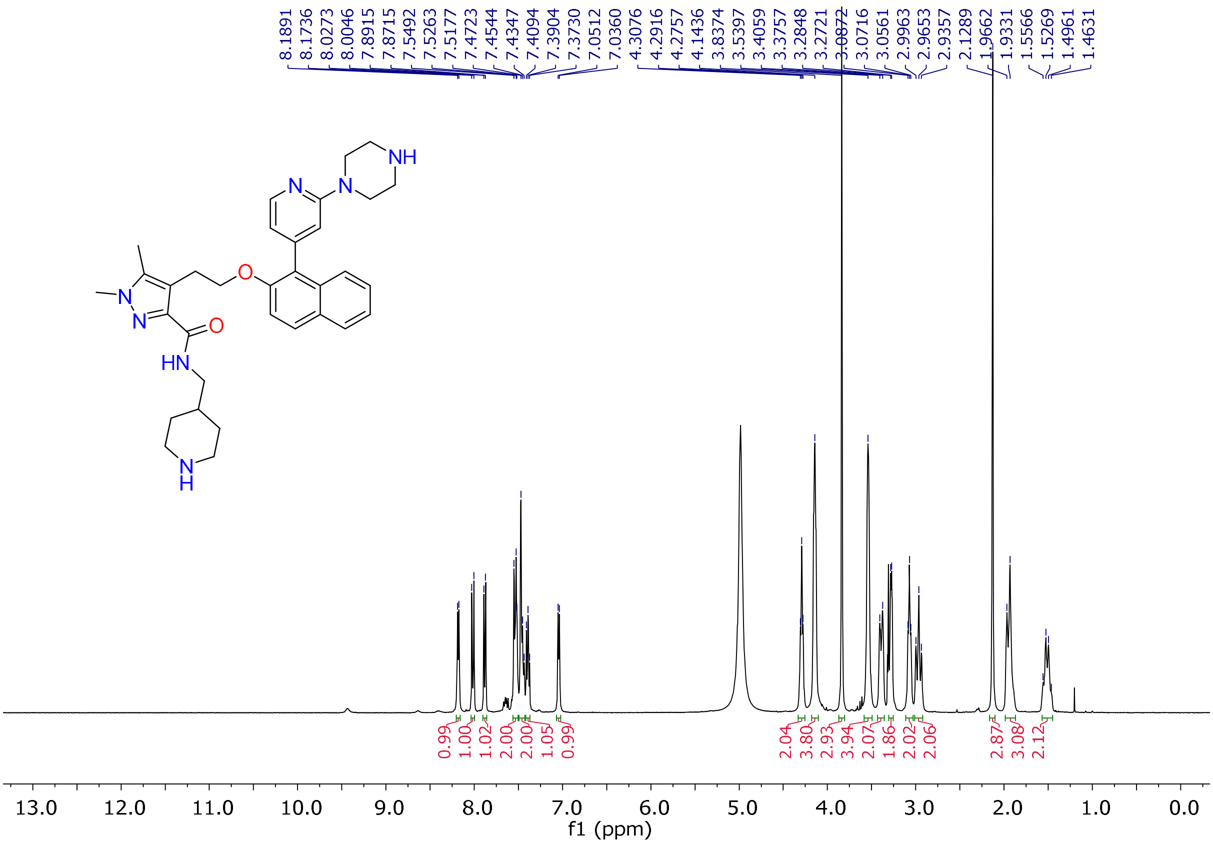

Figure S34. ^{1}H-NMR spectrum of compound **10e** (CD_3OD, 400 MHz).

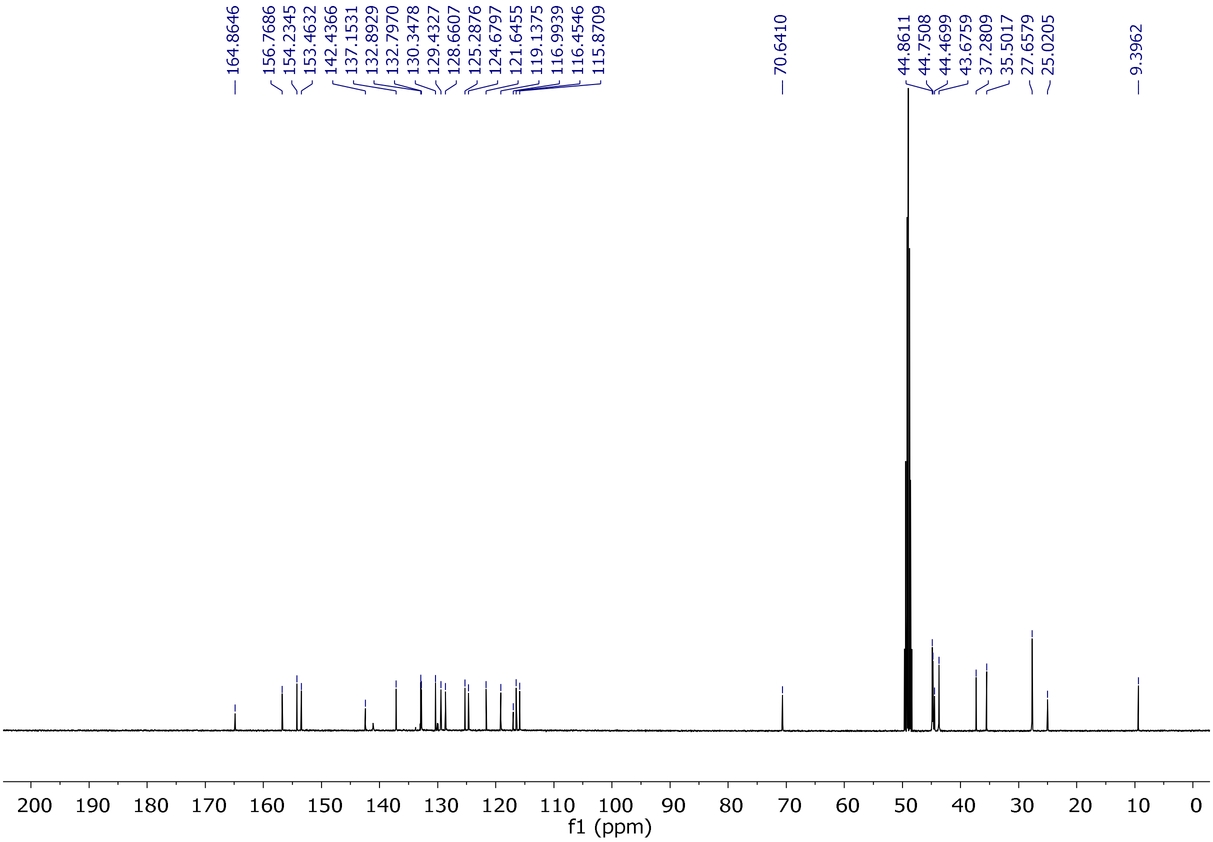

Figure S35. ^{13}C-NMR spectrum of compound **10e** (CD_3OD, 100 MHz).

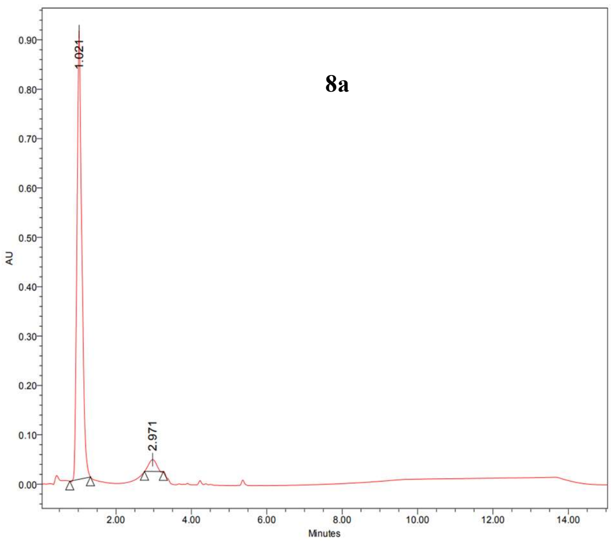
8a
1.021
2.971
AU
Minutes

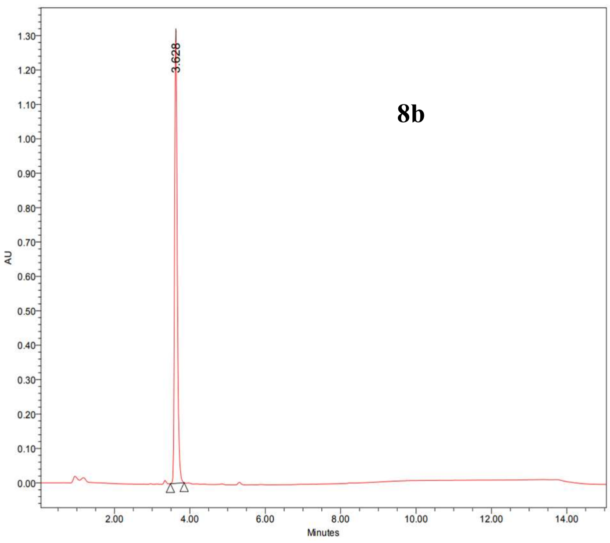
8b
3.628
AU
Minutes

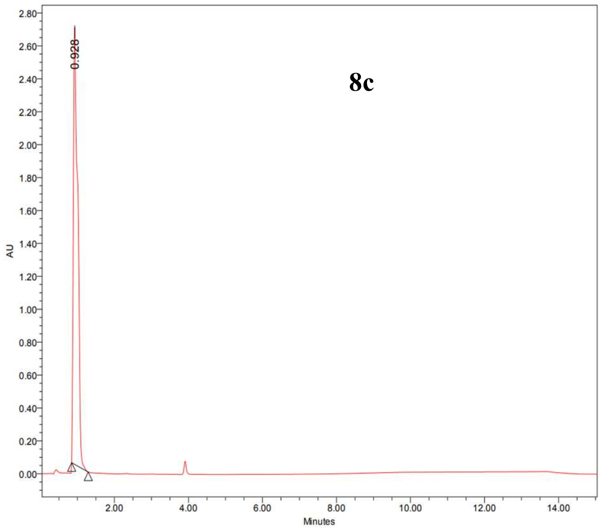
8c
0.928
AU
Minutes

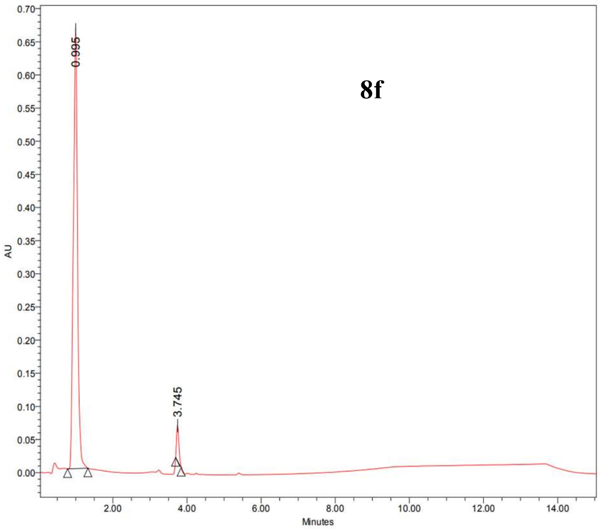
8f
0.995
3.745
AU
Minutes

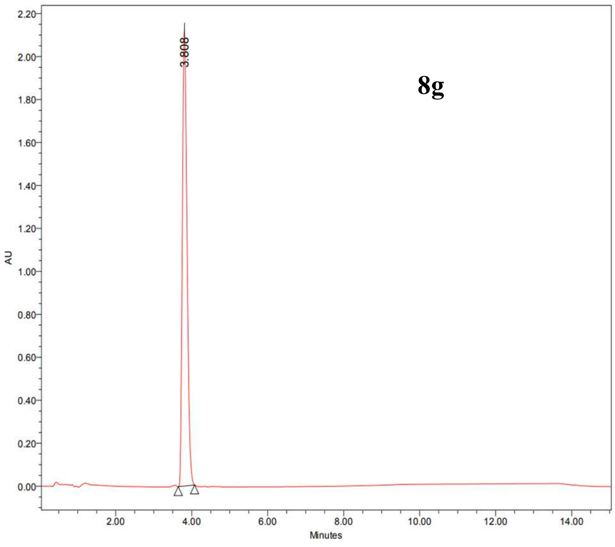
8g
3.808
AU
Minutes

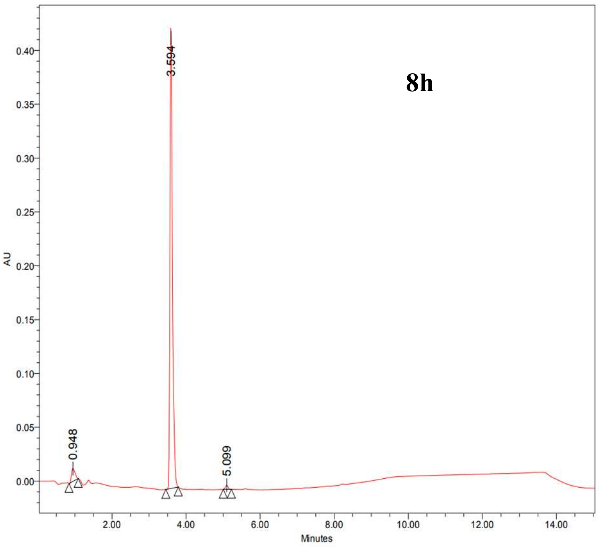
8h
3.594
0.948
5.099
AU
Minutes

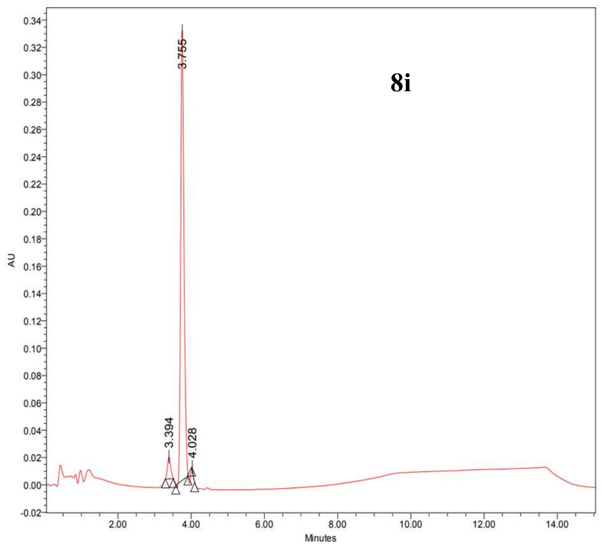
8i
3.755
3.394
4.028
AU
Minutes

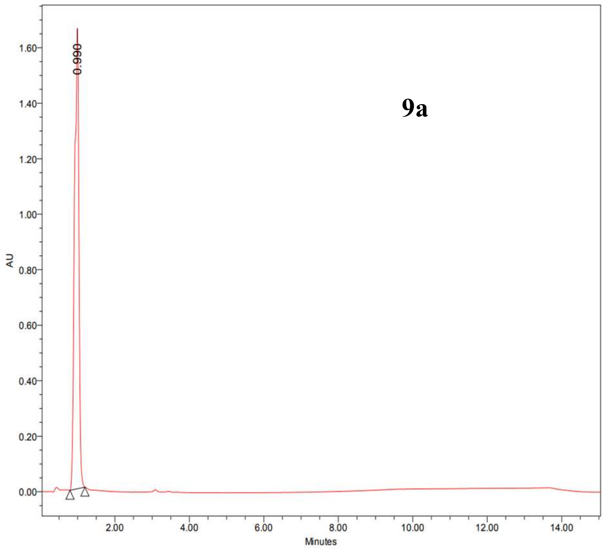
9a
0.990
AU
Minutes

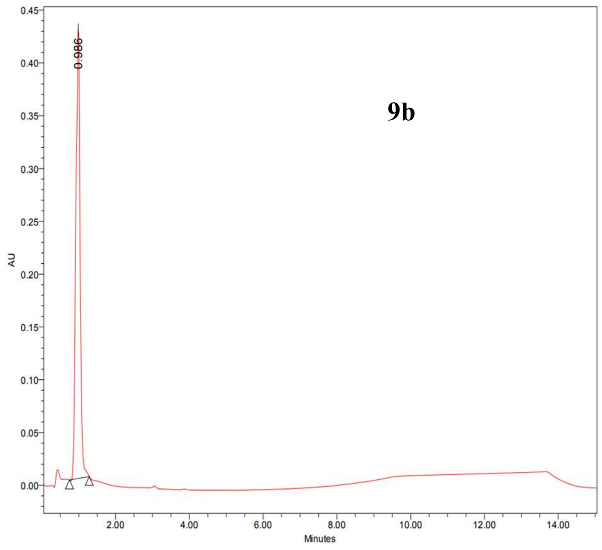
9b
0.986
AU
Minutes

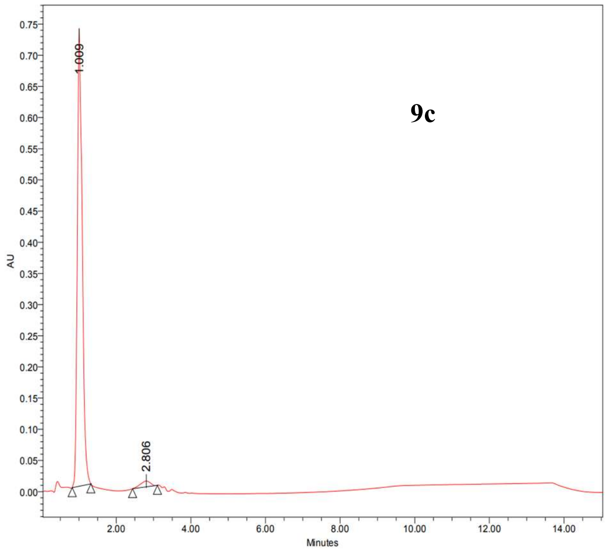
9c
1.009
2.806
AU
Minutes

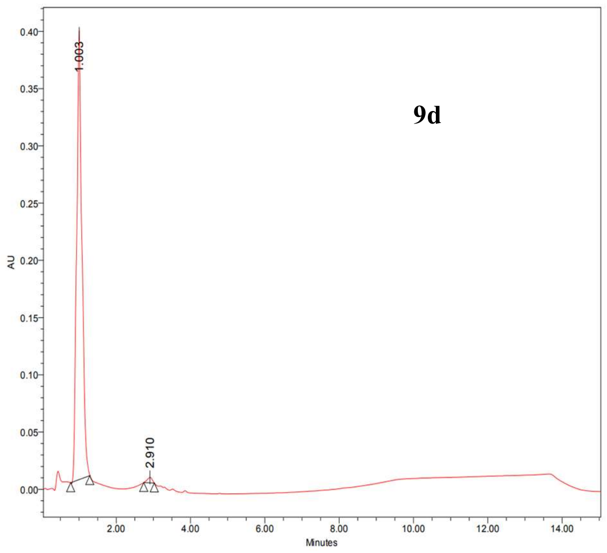
9d
1.003
2.910
AU
Minutes

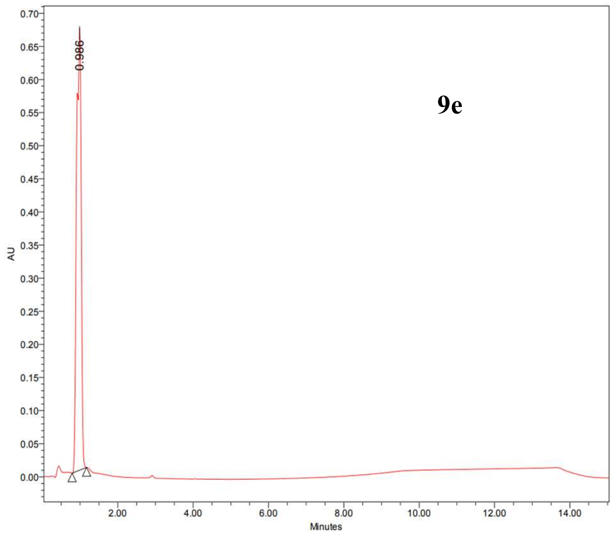
9e
0.986
AU
Minutes

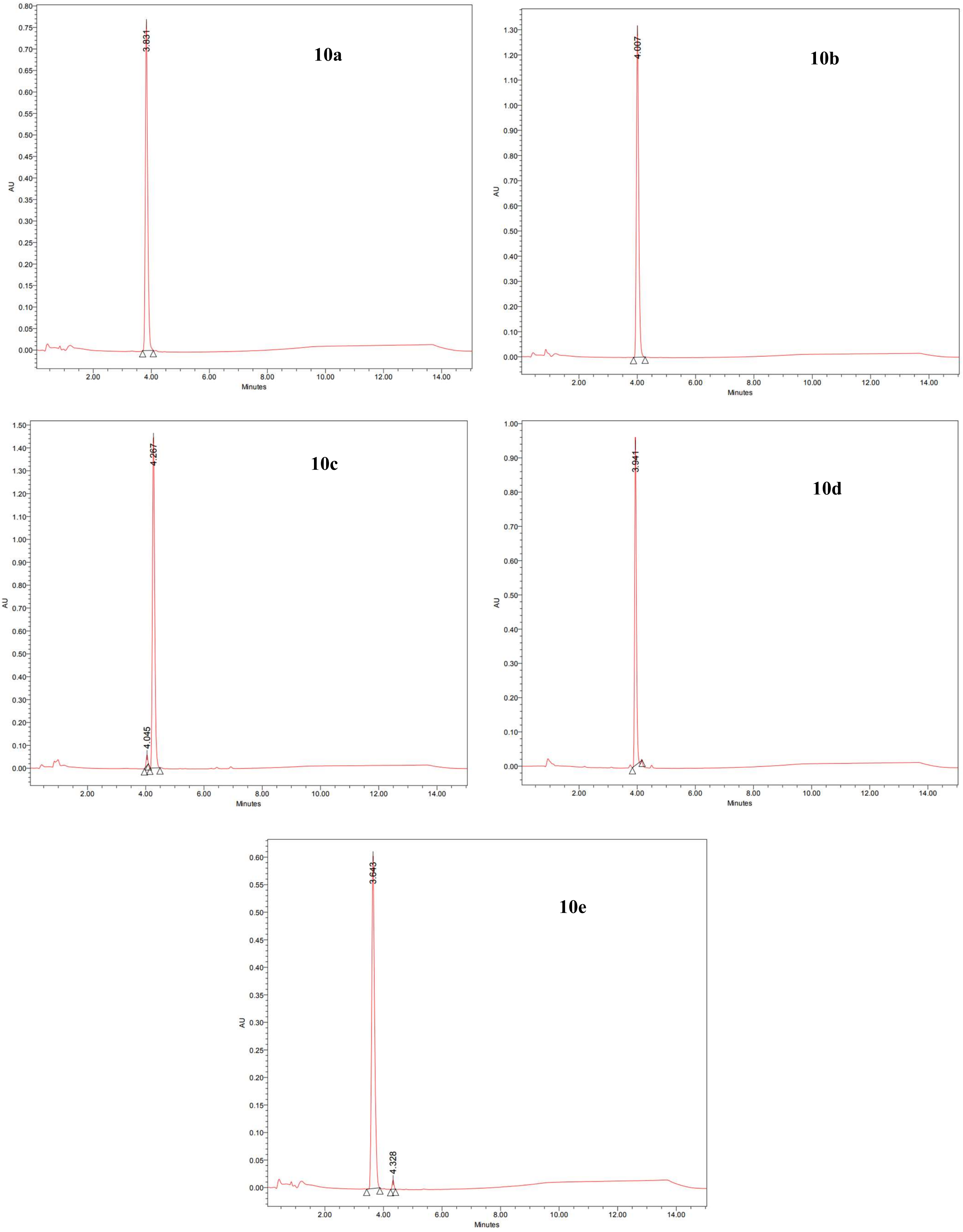

Figure S36. HPLC Chromatogram of NMT inhibitors. (AU = absorbance units).

Table S1. Crystal data collection and refinement statistics.

	*Pv*NMT- myrCoA-**9c**	*Pv*NMT- myrCoA-**10b**
**PDB ID**	8VKA	8VKB
**Data Collection**		
Space group	$P2_12_12_1$	$P2_12_12_1$
Cell dimensions		
a, b, c (Å)	58.340, 119.279, 173.755	57.366, 119.781, 176.883
Resolution (Å)	48.43 – 1.97	48.13 – 2.43
R_{pim}	0.066 (0.469)	0.096 (0.424)
avg $I/\sigma I$	8.0 (1.8)	5.3 (1.6)
Completeness (%)	94.9 (97.4)	99.1 (99.8)
Redundancy	4.6 (4.6)	3.6 (3.7)
**Refinement**		
Resolution (Å)	44.87 – 1.97	48.13 – 2.43
No. of reflections	81,614	46,141
No. of reflections, test set	4,044	2,347
R_{work}/R_{free}	0.157/0.198	0.185/0.233
No. atoms		
Protein	20,289	19,329
Ligand/ion	942	737
Water	973	627
avg *B*-factors (Å^2)		
Protein	30.52	34.62
Ligand/ion	36.32	34.46
Water	35.11	32.00
R.M.S. deviations		
Bond lengths (Å)	0.010	0.002
Bond angles (°)	0.819	0.471
